# Triarylborane-Catalyzed Homogeneous Molecular Transformations Using Organic Reductants

**DOI:** 10.1021/acs.chemrev.6c00439

**Published:** 2026-08-13

**Authors:** Oluwaseyi Aderemi Ajala, Anil Chauhan, Masakazu Tanigawa, Martin Oestreich, Yoichi Hoshimoto

**Affiliations:** † Department of Applied Chemistry, Graduate School of Engineering, 13013 The University of Osaka, Suita, Osaka 565-0871, Japan; ‡ Center for Future Innovation (CFi), Graduate School of Engineering, The University of Osaka, Suita, Osaka 565-0871, Japan; § Institut für Chemie, 26524 Technische Universität Berlin, Straße des 17. Juni 115, 10623 Berlin, Germany

## Abstract

Triarylboranes have emerged as powerful main-group catalysts for the reduction of organic molecules using a variety of reductants, including molecular hydrogen (H_2_), hydrosilanes, Hantzsch esters, and ammonia borane. This development has been largely enabled by the extensive structural diversification of triarylboranes, which allows for systematic tuning of the Lewis acidity and steric environment at the boron center. Since the seminal review of this area more than a decade ago, the field has undergone remarkable growth, prompting this comprehensive account covering three key directions: (1) triarylboranes that, unlike conventional B­(C_6_F_5_)_3_, remain catalytically active in the presence of Lewis bases such as H_2_O, CO_2_, and CO, enabling the use of pure or crude H_2_; (2) diverse organic reductants, including ammonia borane, Hantzsch esters, cyclohexadienes, and other hydride donors; and (3) mechanistic insights into reductant activation and stereocontrol. Covering advances from 2016 to 2025, this review discusses innovations in borane design, substrate scope, functional-group tolerance, and mechanism across each reductant class. By unifying these related yet independent developments, it aims to provide synthetic chemists and theoreticians with timely insights into triarylborane-catalyzed homogeneous reductions, highlighting main-group catalysts as sustainable, practical alternatives to precious-transition-metal systems.

## Graphical abstract

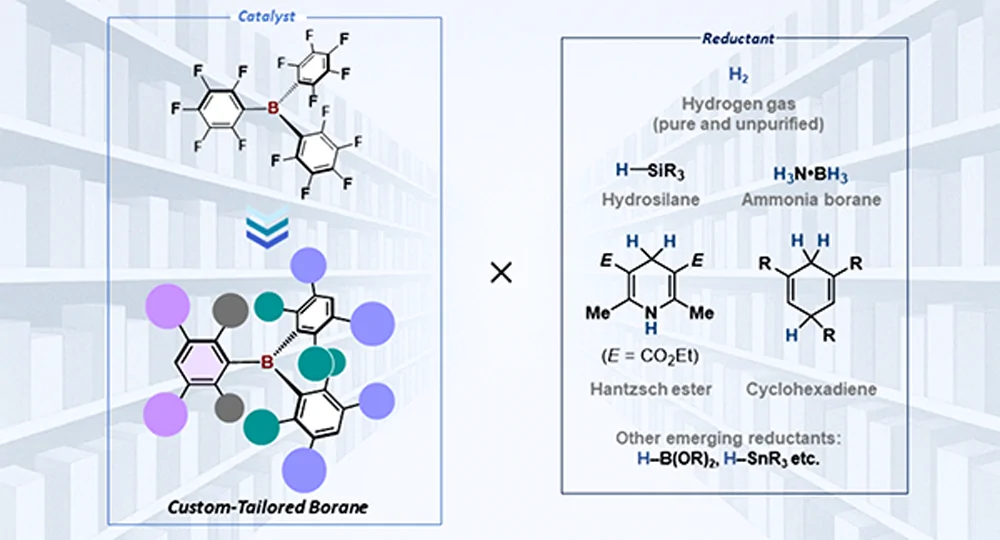

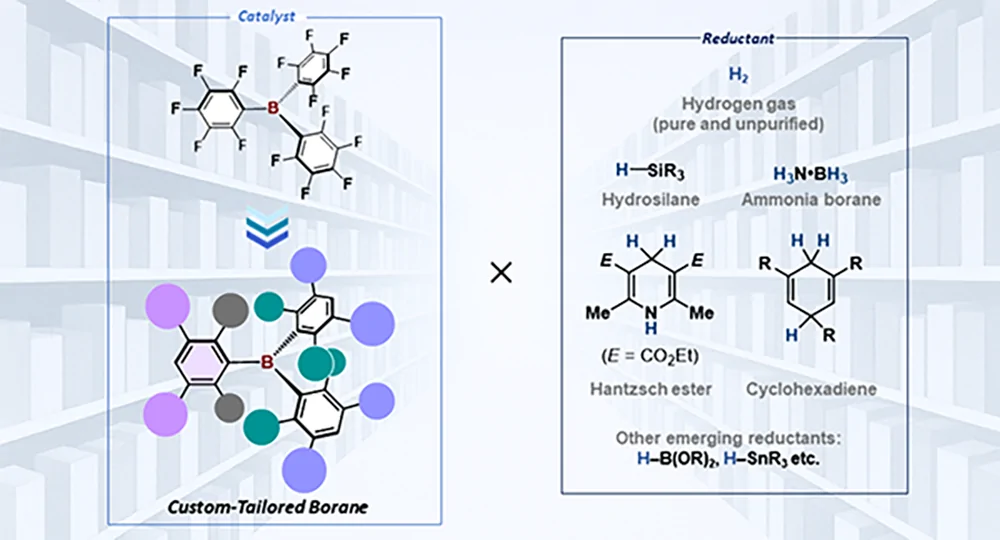

## 1 Introduction

Reduction reactions occupy a central role in organic synthesis, and the development of efficient, selective, and sustainable catalytic systems for their execution has long been a driving force in this field. Transition-metal catalysts have long served as the workhorses for such transformations, offering high activity and selectivity across a broad range of substrate/reductant combinations. However, growing concerns over cost, toxicity, and environmental impact have prompted increasing interest in the use of main-group elements as alternative catalysts.^1–3^ While main-group catalysts have yet to match the breadth and maturity of transition-metal systems, noteworthy fundamental research in this area has flourished, uncovering reaction modes and selectivity profiles that are distinct from—and in some cases inaccessible to—transition-metal-catalyst systems. As a representative example, triarylboranes (BAr_3_) are commonly used as not only electron-deficient components in photo- and electroactive materials but also Lewis acidic catalysts in synthetic chemistry, due to substantial electron-accepting properties arising from the empty p orbital on the boron atom, together with tunable steric and electronic features on the *B*-aryl substituents (Figure 1).^4–14^

**Figure 1:**
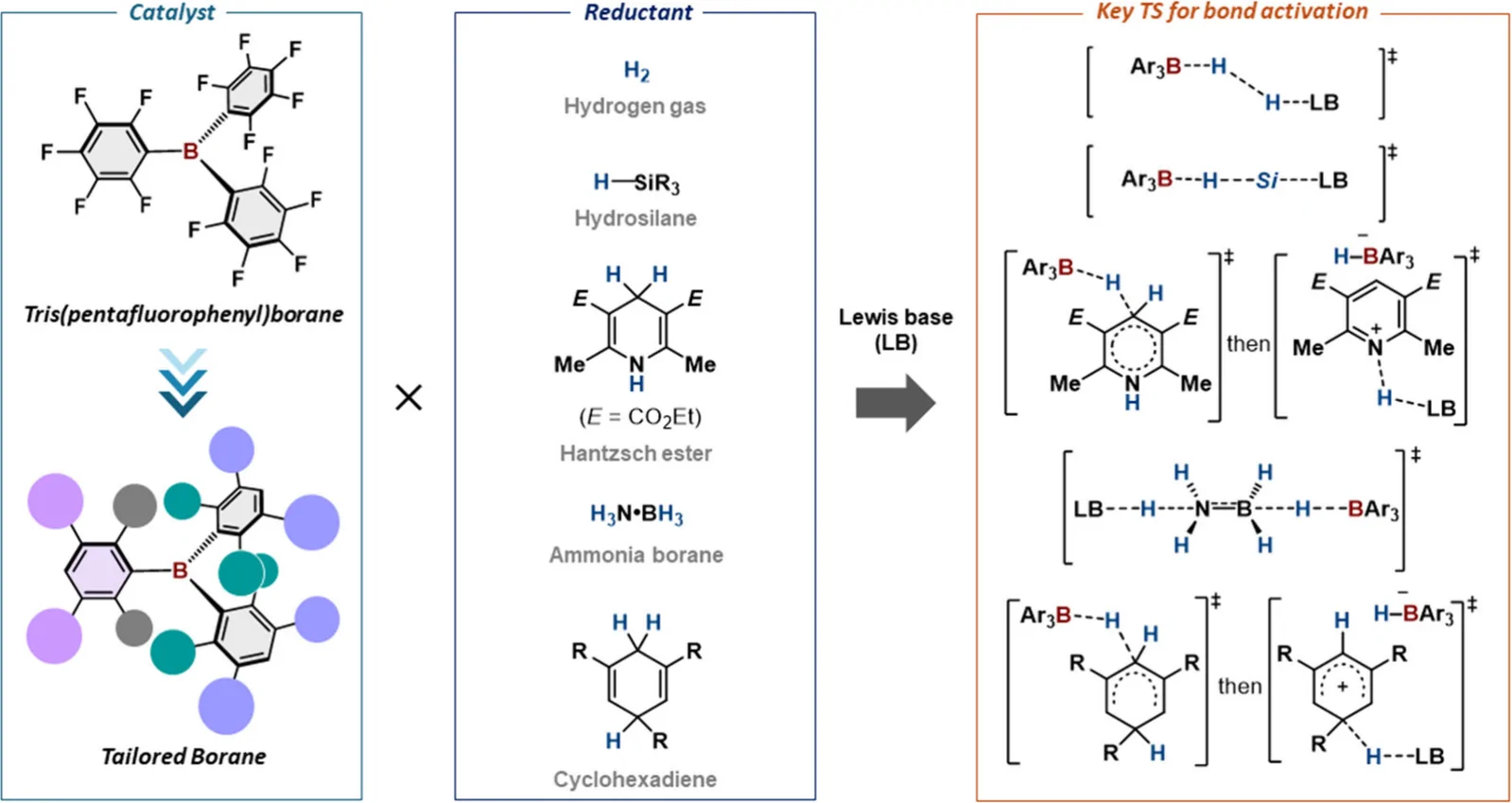
Reductants and their activation mechanisms proposed in BAr_3_-catalyzed reductive transformations.

The structural diversity of BAr_3_ catalysts arises from systematic modification of aryl substituents, enabling precise modulation of so-called Lewis acidity, steric profile, and stability. Strongly electron-withdrawing groups (e.g., perfluoroaryl) enhance boron electrophilicity, while steric bulk governs substrate access and mitigates catalyst deactivation. These parameters collectively dictate reactivity, selectivity, and functional group tolerance.^4,9,12,13^ Additionally, incorporation of heteroatom-substituted or π-extended frameworks allows fine-tuning of electronic properties and improves catalytic robustness, establishing BAr_3_ as a versatile platform for metal-free reduction catalysis.

A pivotal advance came with the recognition that pairing BAr_3_ with an appropriate Lewis base enables the cooperative heterolytic cleavage of hydrosilanes and H_2_ (Figure 1), a reactivity mode widely acknowledged as Piers-type and frustrated Lewis pair (FLP)-type activation of such H–E bonds (E = Si and H), respectively.^8,15–25^ In this context, in 2015, one of the authors (M.O.) systematically summarized in a comprehensive review the early developments in BAr_3_-catalyzed reductions employing B­(C_6_F_5_)_3_ (and a few derivatives) as a catalyst, together with hydrosilanes and H_2_ as reductants.^5^ Subsequent studies demonstrated that BAr_3_ catalysis is likewise effective with crude H_2_, Hantzsch esters, ammonia borane, and other organic reductant systems, underscoring the ability of these strongly Lewis acidic catalysts to activate hydride donors and promote efficient metal-free reduction processes. Since then, the field has expanded significantly; however, a unified or comparative review of these reductant systems, including pure/crude H_2_, hydrosilanes, ammonia borane, Hantzsch esters, hydroboranes, and related hydride sources, remains relatively underdeveloped. Encouragingly, recent advances have begun to demonstrate that BAr_3_ catalysis is not merely a metal-free surrogate for transition-metal systems but harbors the potential to enable transformations that are genuinely distinct, and in some cases only accessible via main-group catalysis.

In this review, we update previous key reviews to provide a comprehensive overview of recent advances in BAr_3_-catalyzed homogeneous molecular transformations employing a diverse array of reductants, including pure and crude H_2_, hydrosilanes, Hantzsch esters, H_3_N·BH_3_, cyclohexadienes, and borane-based hydride donors such as pinacolborane, covering the literature from 2016 through 2025; heterogeneous systems are outside the scope of this review. This review also covers the development of FLP-catalyzed asymmetric reduction reactions, although some of these examples do not employ BAr_3_ but rather mono- or diarylboranes, because the fundamental reaction mechanisms for activating reductants are similar.^26–30^ Mechanistic investigations of BAr_3_-catalyzed systems with these hydrogen donors have revealed distinct activation modes, enabling a range of synthetically useful transformations across broad substrate classes (Figure 1). A key driver of this expansion has been the deliberate design of BAr_3_ catalysts beyond the prototypical B­(C_6_F_5_)_3_. Focus has increasingly shifted from using B­(C_6_F_5_)_3_ as a convenient off-the-shelf reagent toward the rational design of tailored BAr_3_, yielding catalysts with improved shelf stability and enhanced turnover numbers (TONs) as well as reactivity profiles inaccessible to B­(C_6_F_5_)_3_ itself. This review therefore also covers the synthetic approaches to such BAr_3_ alongside their catalytic applications, providing a unified account of catalyst design and reactivity. Note that, unless otherwise noted, reagents are used in 1 equivalent relative to the respective reference compound in each scheme throughout this review; equivalents are specified only when the amount differs. The structures of the triarylboranes discussed in this review are shown in Figure 2

**Figure 2:**
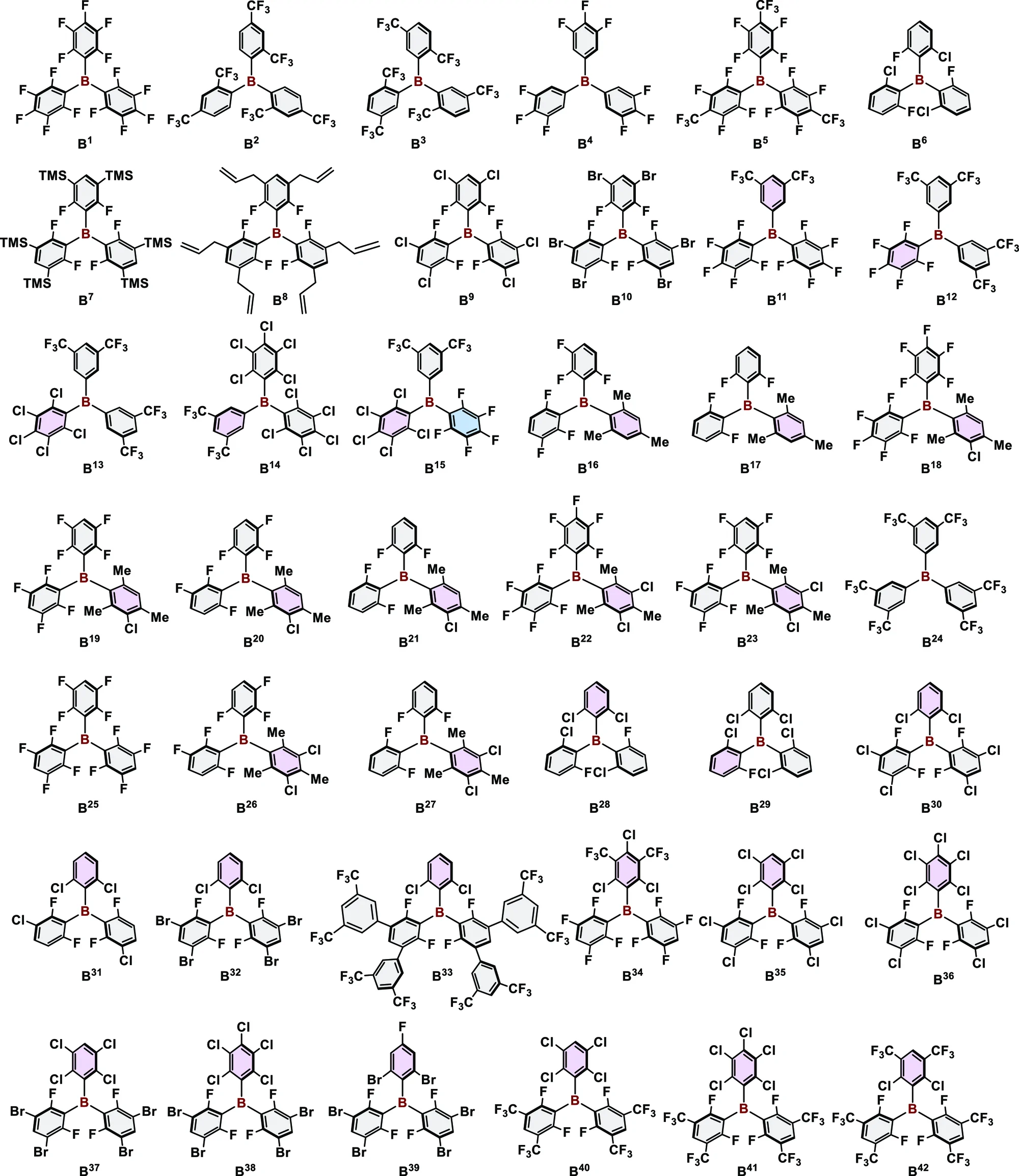

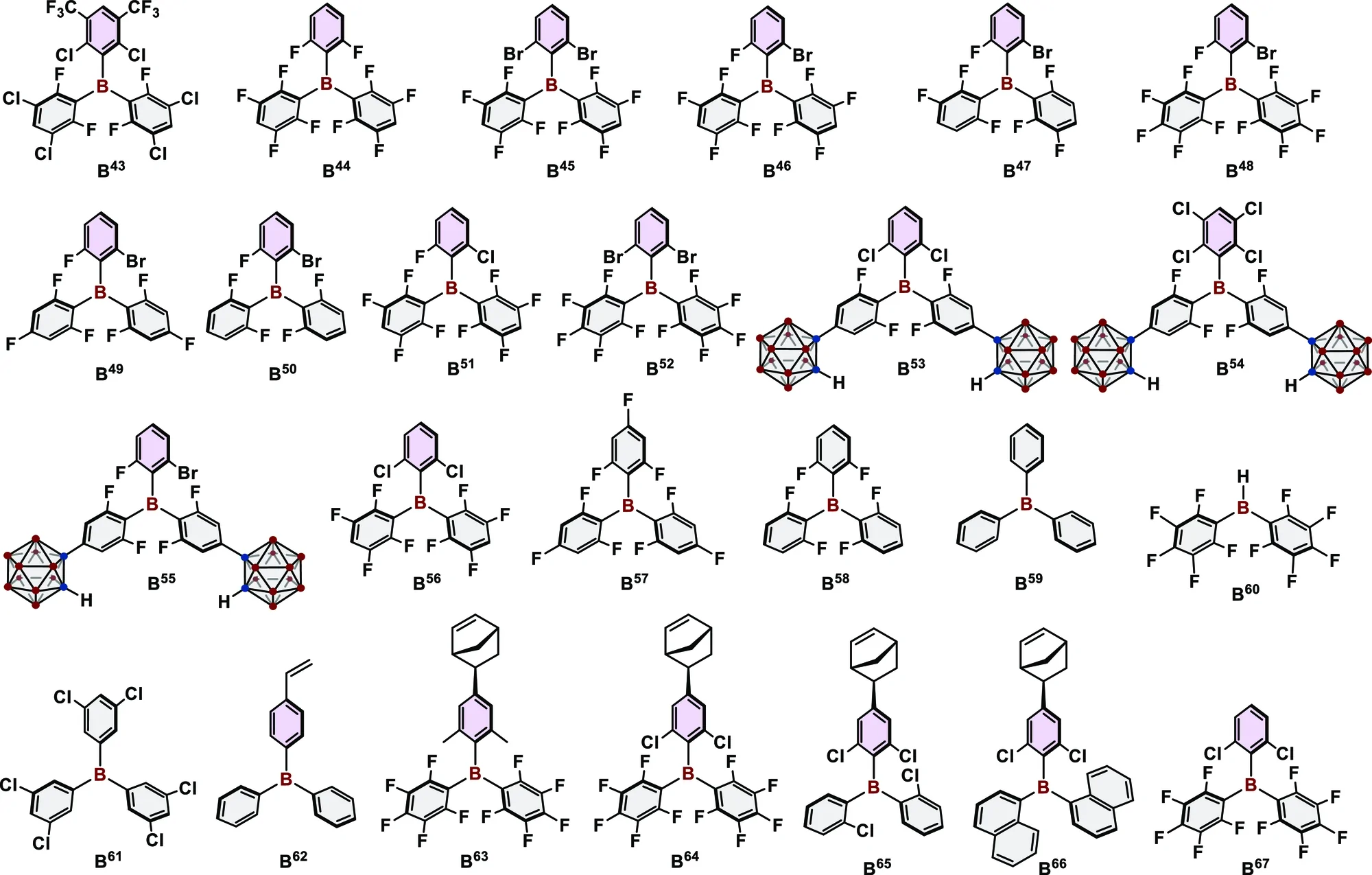
Structures of the triarylboranes discussed in this review.

## 2 Structural Diversity and Design Principles of Triarylborane Catalysts

### 2.1 Design and Preparation of Homoleptic Halogenated Triarylboranes

Following the seminal synthesis of B­(C_6_F_5_)_3_ (**B** ^**1**^),^31–33^ the preparation of halogenated BAr_3_ has expanded significantly, driven by their exceptional Lewis acidity and broad catalytic utility.^34^ Recent developments (2016–2026) mark a transition from empirical synthetic approaches toward rational, structure–reactivity-guided design. Although such BAr_3_ are still predominantly based on polyfluoroaryl groups, relatively little is known about their environmental persistence. On one hand, **B** ^**1**^ itself does not meet the current regulatory definition of per- and polyfluoroalkyl substances (PFAS; OECD 2021), a class that includes well-known “forever chemicals”, because the definition requires at least one fully fluorinated aliphatic −CF_3_ or −CF_2_– moiety, whereas the fluorines in **B** ^**1**^ reside exclusively on aromatic rings.^35^ On the other hand, the polyfluoroarene fragments associated with these boranes, such as the pentafluorobenzene used in the synthesis of **B** ^**1**^ or released upon its protodeboronation, contain aryl C–F bonds that are among the strongest single bonds in organic molecules and are correspondingly resistant to (bio)­degradation; their long-term environmental fate thus remains largely uncharacterized.^36^

To achieve precise control over the catalytic performance of boranes, recent catalyst design has increasingly focused on systematically refining the steric and electronic environment around the boron center. One principal strategy involves modulation of substituent identity and position, allowing Lewis acidity and substrate accessibility to be tuned independently. For example, lithium–halogen exchange followed by trapping with BCl_3_ enables access to homoleptic boranes with controlled CF_3_ substitution patterns (Scheme 1).^37^ Variations in substituent position reveal that *o*-CF_3_ groups induce intramolecular B···F interactions and steric shielding, reducing effective Lewis acidity and suppressing FLP reactivity, whereas *meta*-substituted analogues retain higher electrophilicity due to the absence of such interactions.

**Scheme 1:**
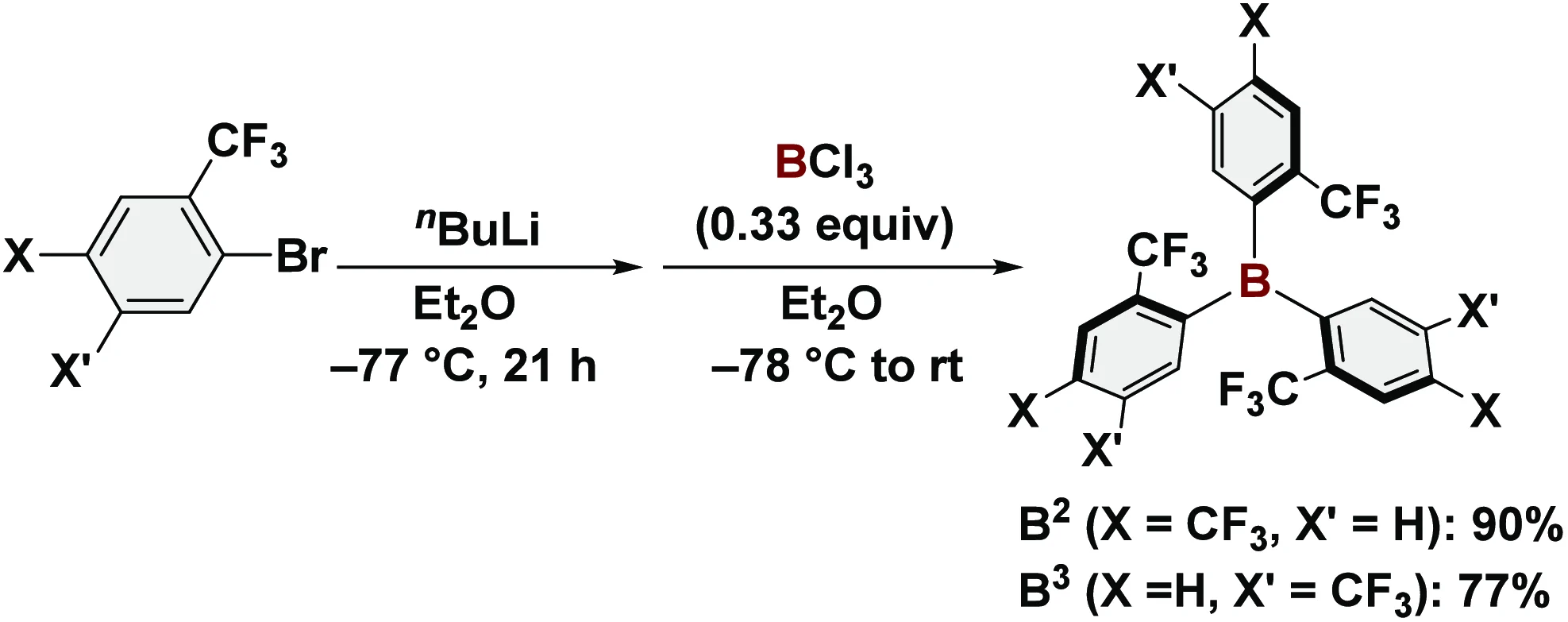
Synthesis of **B** ^**2**^ and **B** ^**3**^

Decoupling steric effects from intrinsic Lewis acidity has also been achieved through the removal of *o*-fluorine substituents (Scheme 2).^38^ Electron-deficient boranes lacking *ortho*-substitution maintain strong electrophilicity while enhancing accessibility at the boron center, facilitating substrate coordination and downstream reactivity. This highlights that catalytic performance is governed not only by intrinsic Lewis acidity but also by the steric accessibility of boron.

**Scheme 2:**
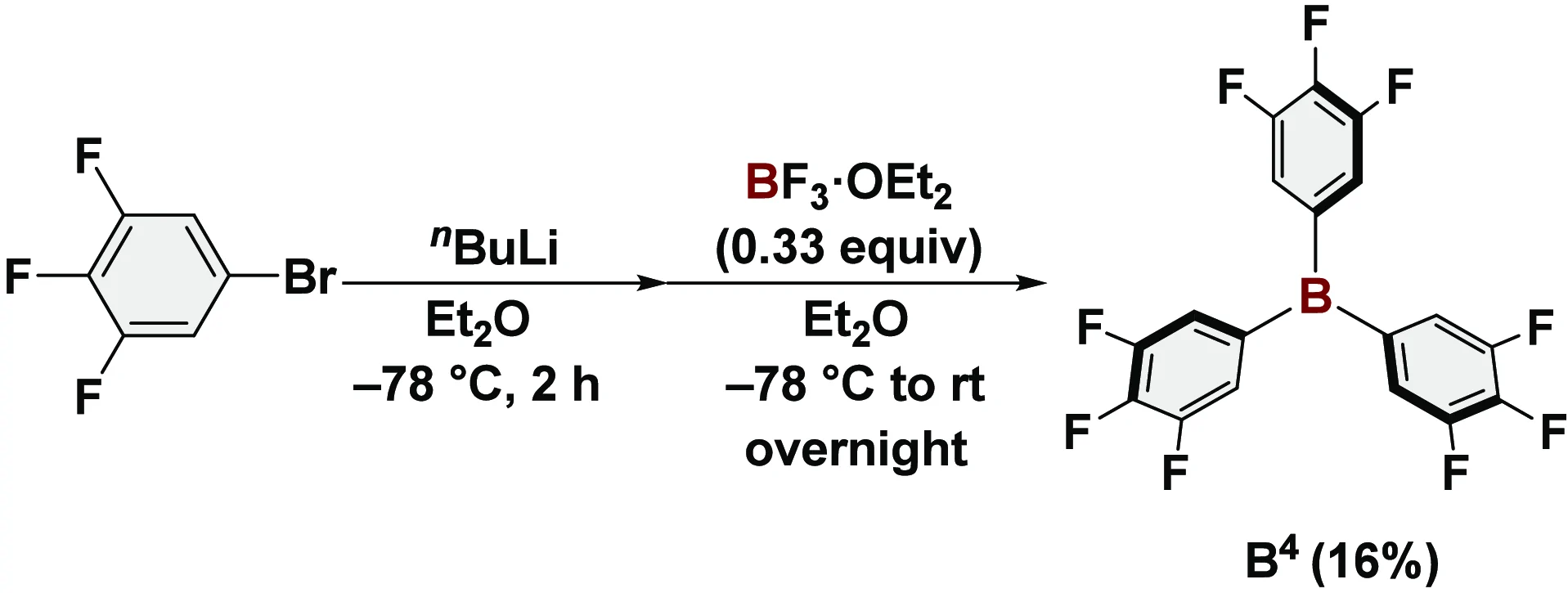
Synthesis of **B** ^**4**^

While lithium–halogen exchange remains a powerful method for accessing BAr_3_, the practical considerations associated with fluoroaryllithium species should also be recognized. Fluoroaryllithium species generated *in situ* from organolithium reagents are highly moisture-sensitive and can exhibit limited thermal stability, requiring careful temperature control and handling under an inert atmosphere.^39–41^ Alternative approaches employing fluoroaryl Grignard reagents may offer improved operational robustness in other synthetic routes.^42,47–53^

Further advances have targeted the development of highly electron-deficient boranes.^42^ Incorporation of strongly electron-withdrawing perfluorotolyl substituents yields systems with enhanced electrophilicity and propeller-like geometries that influence both electronic structure and torsional behavior (Scheme 3).^43^ Such boranes exhibit Lewis superacidity relative to that of **B** ^**1**^, underscoring the importance of substituent electronics and conformational effects.

**Scheme 3:**
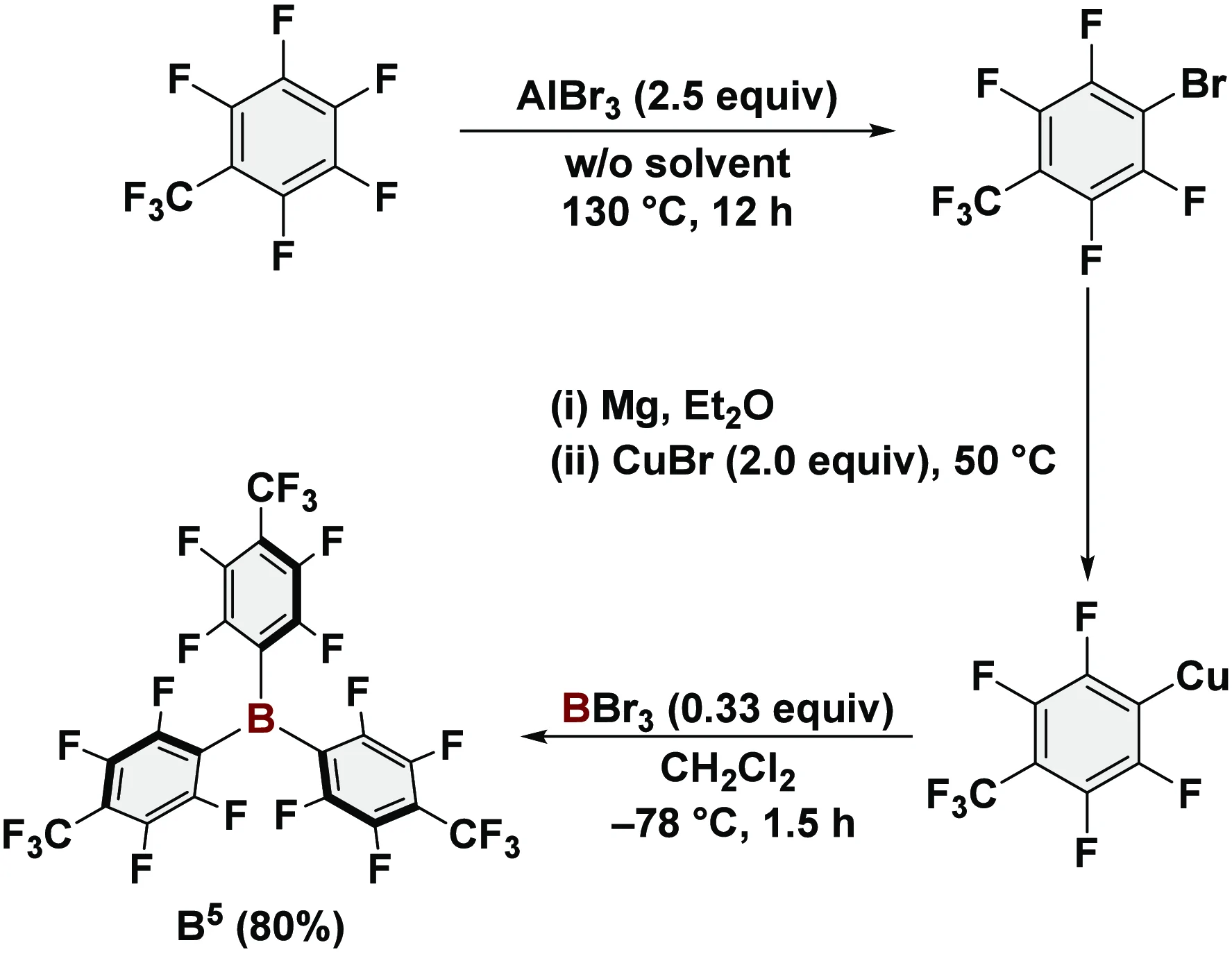
Synthesis of **B** ^**5**^

Beyond purely electronic tuning, steric strain has emerged as a key design parameter. The effective Lewis acidity of BAr_3_ reflects a balance between front strain (hindering the Lewis base approach) and back strain (destabilizing the tetrahedral adduct formed upon coordination) (Figure 3). Larger halogen substituents introduce increased steric congestion, enhancing reversible binding by destabilizing the coordinated state.^4,13,44–47^ This interplay between steric repulsion and electronic effects governs both the reactivity and selectivity.

**Figure 3:**
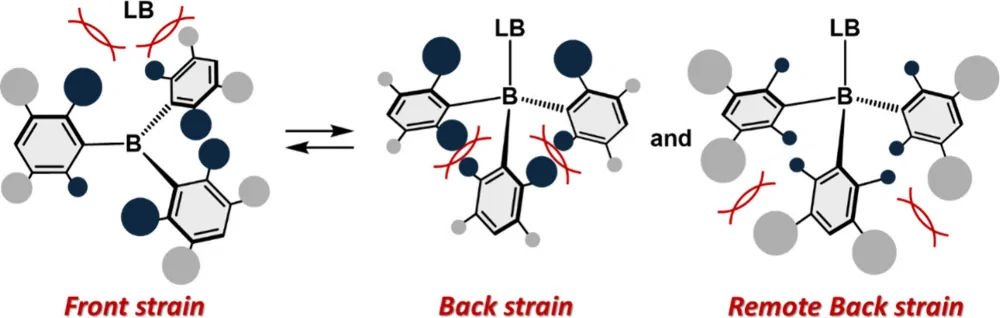
Schematic illustration of front strain, generated between BAr_3_ and Lewis bases (LBs), and (proximal and remote) back strain, generated among aryl groups in the four-coordinate adducts.

In addition to electronic and steric approaches, geometric distortion has emerged as an alternative strategy for enhancing the Lewis acidity of triarylboranes. Berionni and co-workers demonstrated that constraining the boron center within a triptycene-derived framework affords pyramidalized triarylboranes exhibiting exceptionally high Lewis acidity, despite the absence of extensive fluorination (Scheme 4).^48–50^ The enhanced Lewis acidity originates from the preorganization of the boron center and reduced structural reorganization upon Lewis base coordination, highlighting geometric distortion as a complementary design principle for the development of highly electrophilic boranes and Lewis superacids. However, their catalytic applications are outside the scope of this review.^51^

**Scheme 4:**
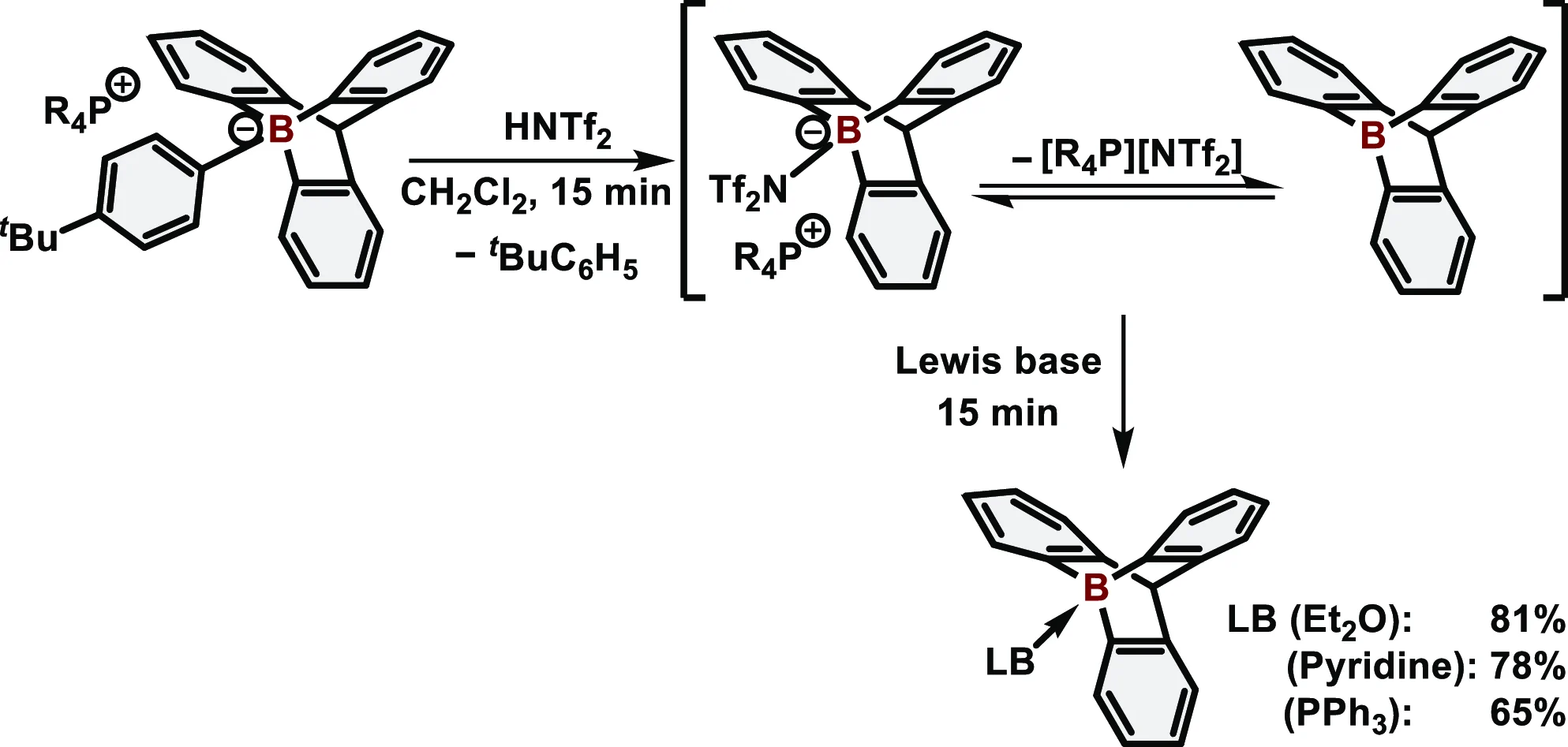
Synthesis of Pyramidal Boratriptycene Lewis Acids and Representative Lewis Adducts (R = ^*t*^Bu, Ph, etc.)

Complementary to these catalyst design strategies, systematic halogen substitution has been used to fine-tune steric environments. Progressive fluorine-to-chlorine substitution enables controlled modulation of front and back strain (Scheme 10), providing a platform to investigate their influence on Lewis acidity, water tolerance, and FLP reactivity.^52^

A more refined strategy involves remote back-strain engineering, in which substituents located distal to the boron center modulate deformation energy and noncovalent interactions without significantly altering intrinsic electronic properties (Figure 3). This approach enables independent tuning of effective Lewis acidity and catalytic reactivity, as demonstrated by boranes bearing *meta* substituents (Schemes 5 and 6) that enhance steric repulsion and improve performance in hydrogenation reactions, including under impurity-containing H_2_ streams.^47,53^

**Scheme 5:**
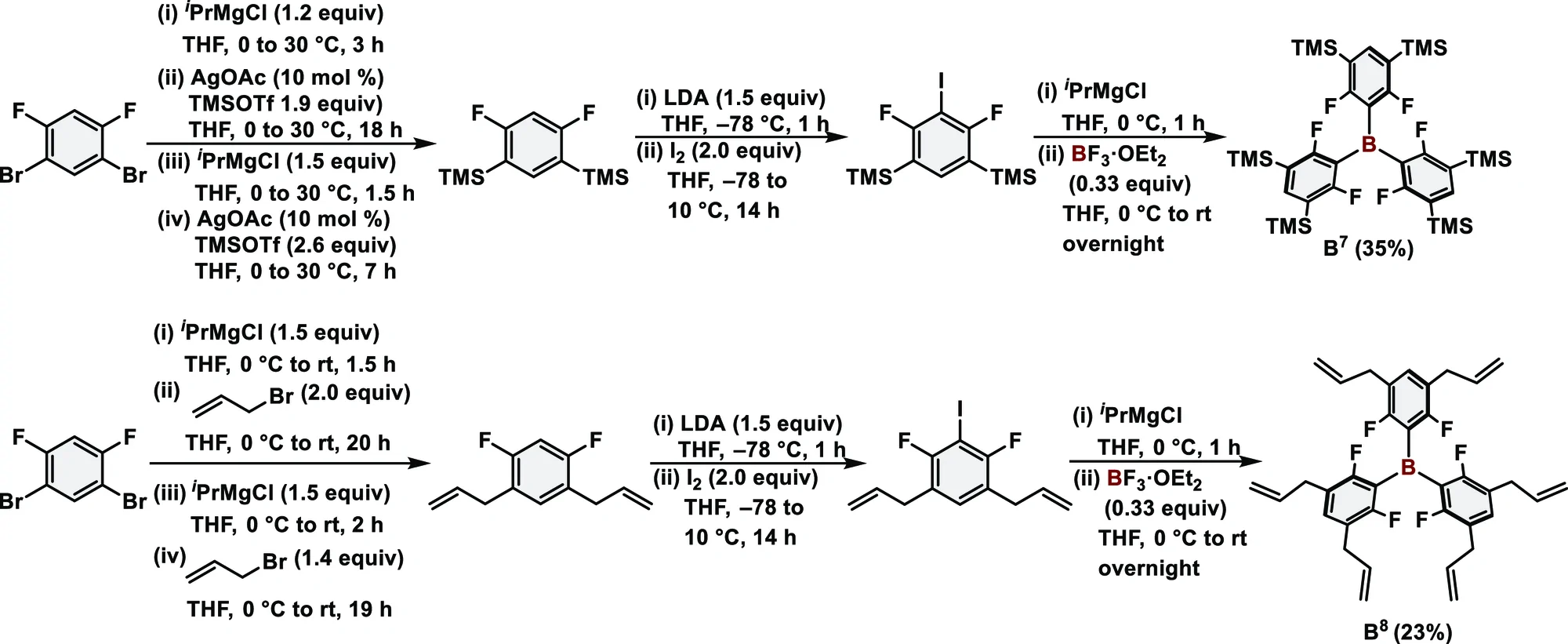
Synthesis of **B** ^**7**^ and **B** ^**8**^ (LDA, Lithium Diisopropylamide)

**Scheme 6:**
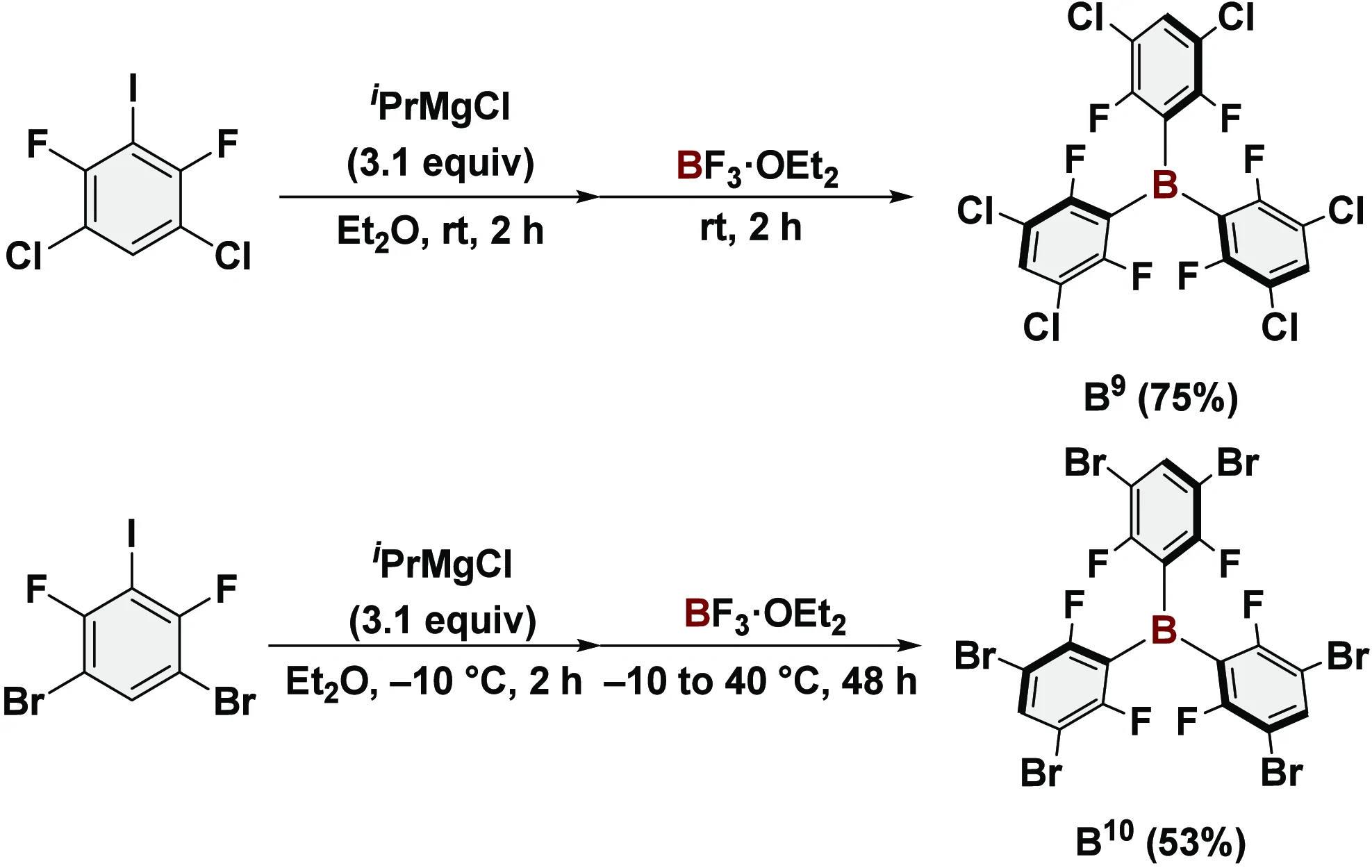
Synthesis of **B** ^**9**^ and **B** ^**10**^

Despite these advances, optimal catalyst performance remains confined to a narrow multidimensional window defined by Lewis acidity, steric accessibility, and reversible binding.^54^ This complexity has motivated the development of predictive design strategies. Recent computational approaches, such as virtual borane models, enable independent tuning of steric and electronic parameters, allowing for rapid prediction of reaction profiles and catalyst performance.^55^ These methods represent a shift toward data-driven catalyst design, moving beyond traditional trial and error approaches.

### 2.2 Design and Preparation of Heteroleptic Halogenated Triarylboranes

The Lewis acidity and catalytic performance of halogenated BAr_3_ can be tuned not only by varying the substituent identity and position but also through heteroleptic architectures comprising electronically and sterically differentiated aryl groups. Such designs enable independent modulation of intrinsic and effective Lewis acidity within a single borane scaffold, thereby expanding their versatility in catalysis.

A foundational approach to heteroleptic borane design involves the stepwise substitution of aryl groups to systematically vary electronic and steric environments. Incorporation of pentafluorophenyl, bis­(trifluoromethyl)­phenyl, and pentachlorophenyl substituents enables controlled comparison of inductive and steric effects within a common trigonal-planar framework (Schemes 7 and 8).^56,57^ While electronically similar substituents primarily influence electrophilicity, incorporation of bulkier aryl groups introduces significant steric effects, including increased aryl-ring twist and through-space interactions that attenuate effective Lewis acidity despite a strong electron-withdrawing character. These studies demonstrate that Lewis acidity arises from a balance between inductive withdrawal and steric distortion imposed by *ortho*-substitution.

**Scheme 7:**
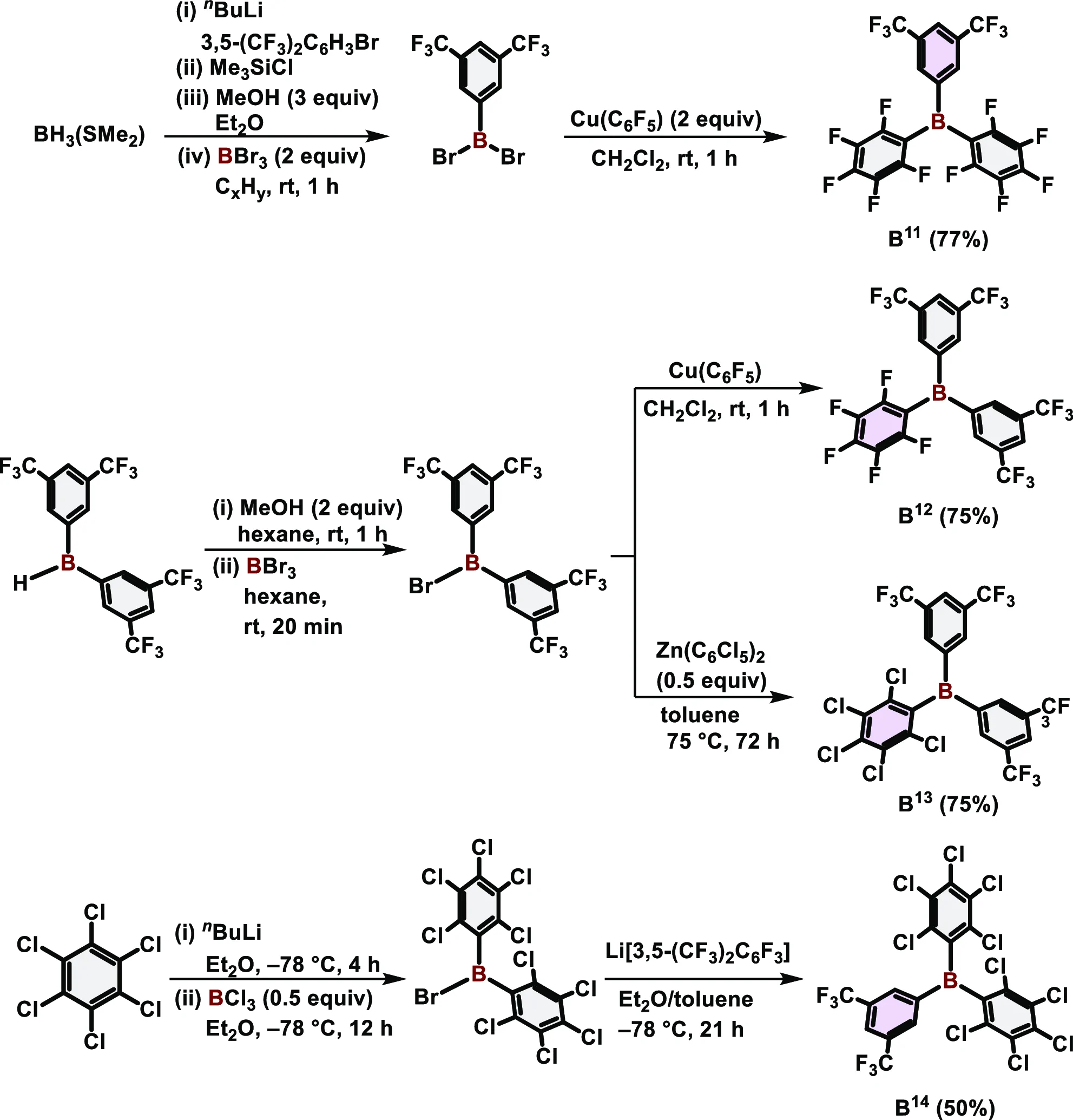
Synthesis of **B** ^**11**^, **B** ^**12**^, **B** ^**13**^, and **B** ^**14**^

**Scheme 8:**
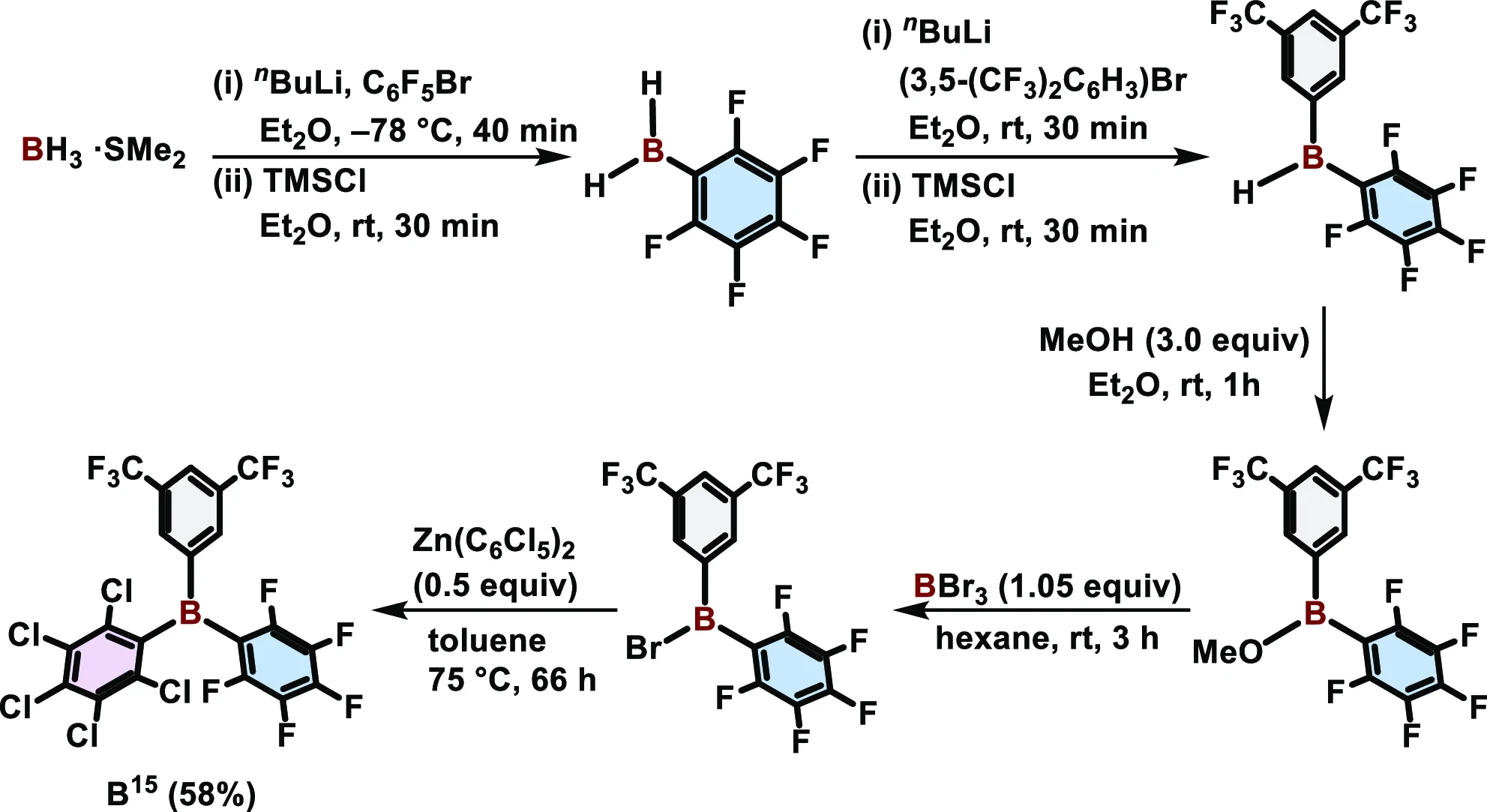
Synthesis of **B** ^**15**^

Systematic tuning of electronic properties while maintaining steric consistency was also achieved through modular synthetic strategies. Mesityl-based boranes incorporating fluorinated aryl substituents allow precise control of electrophilicity via *meta* and *para* substitution, with *meta*-fluorination significantly enhancing Lewis acidity and catalytic performance (Scheme 9).^58^ In contrast, substitution on sterically demanding aryl frameworks can exert a minimal electronic influence, highlighting the decoupling of steric and electronic effects in heteroleptic systems.

**Scheme 9:**
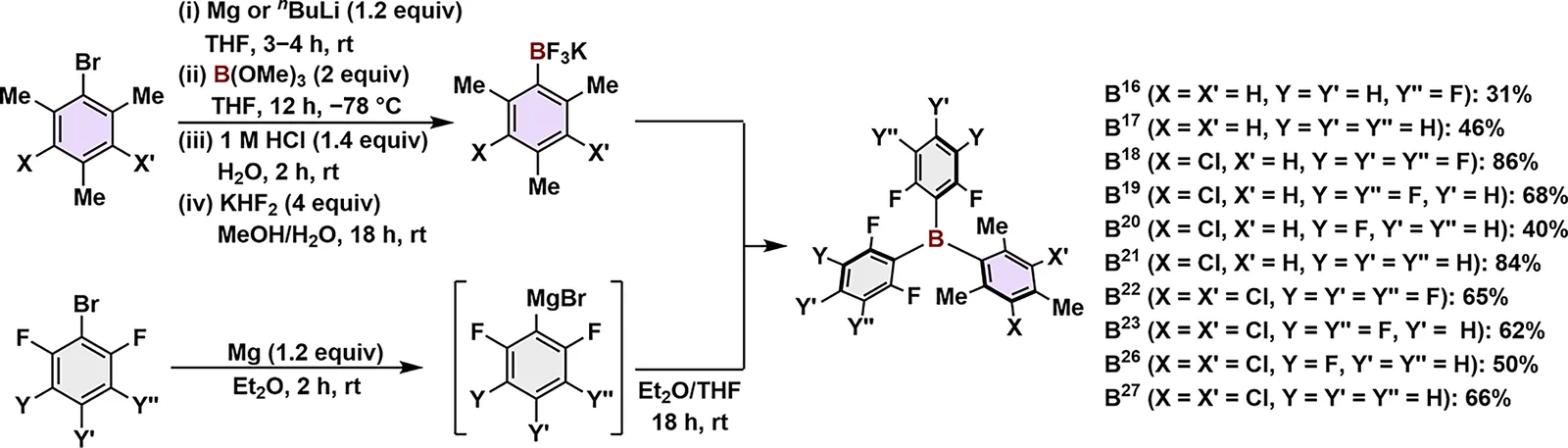
Synthesis of **B** ^**16**^–**B** ^**27**^

Building on these principles, progressive halogen-exchange strategies have been employed to modulate both front and back strain at the boron center. Incremental fluorine-to-chlorine substitution increases steric congestion and deformation energy associated with boron coordination, thereby tuning effective Lewis acidity and enabling reversible substrate binding (Scheme 10).^52,59^ These effects are further refined through remote back-strain engineering, where distal substituents modulate intramolecular repulsion and noncovalent interactions without significantly altering intrinsic electronic properties.

**Scheme 10:**
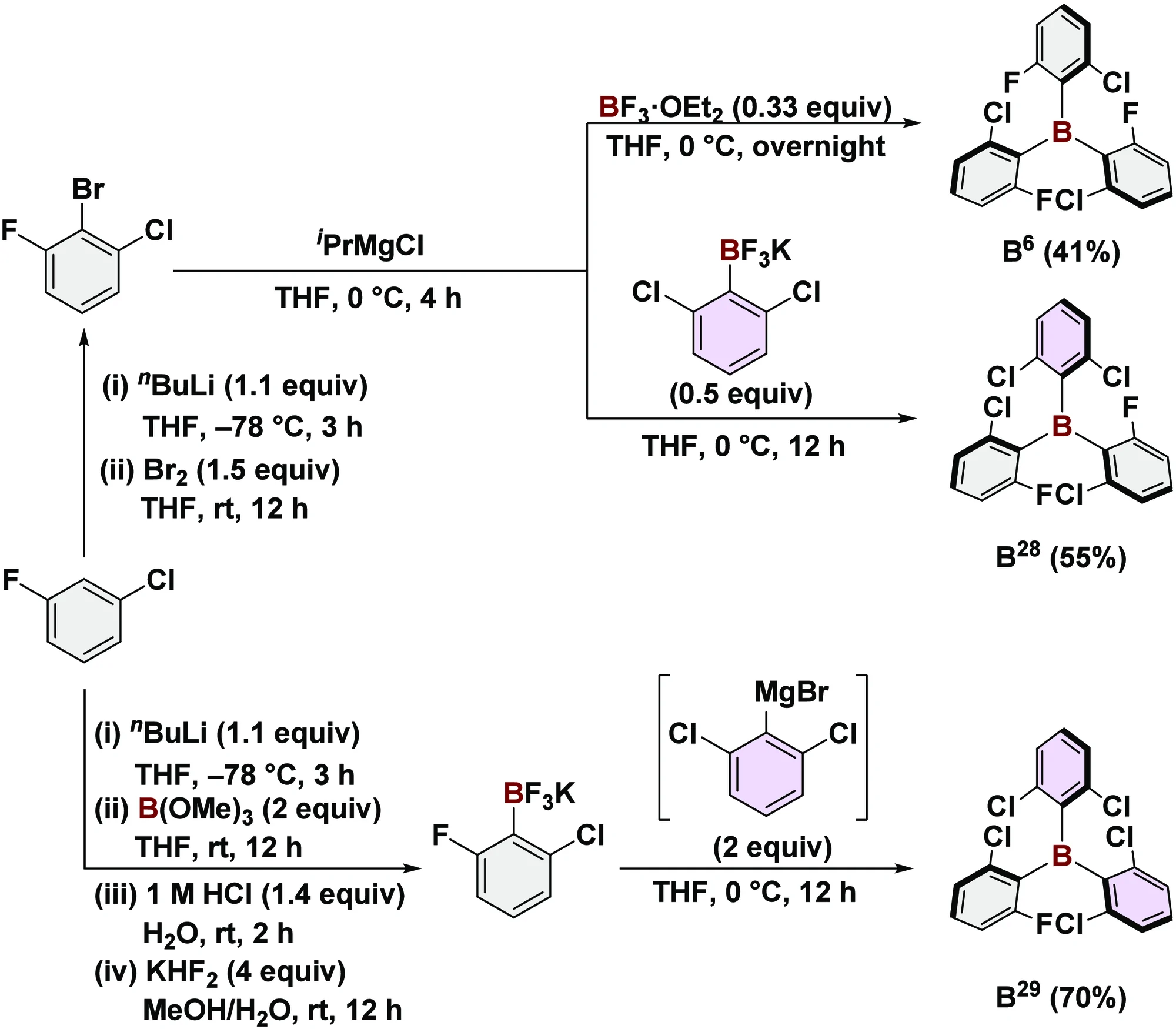
Synthesis of **B** ^**6**^, **B** ^**28**^, and **B** ^**29**^

Recent advances have demonstrated the power of combining steric and electronic tuning within heteroleptic frameworks. Rational design of boranes incorporating *m*-halogen and 2,6-dichloroaryl substituents (Scheme 11) enables suppression of catalyst deactivation by CO, CO_2_, and H_2_O while preserving efficient H_2_ activation.^60^ Extension to modular libraries of heteroleptic boranes (Scheme 12) further highlights the utility of remote back-strain concepts, enabling enhanced catalytic performance under impurity-rich conditions through destabilization of off-cycle Lewis adducts.^61^

**Scheme 11:**
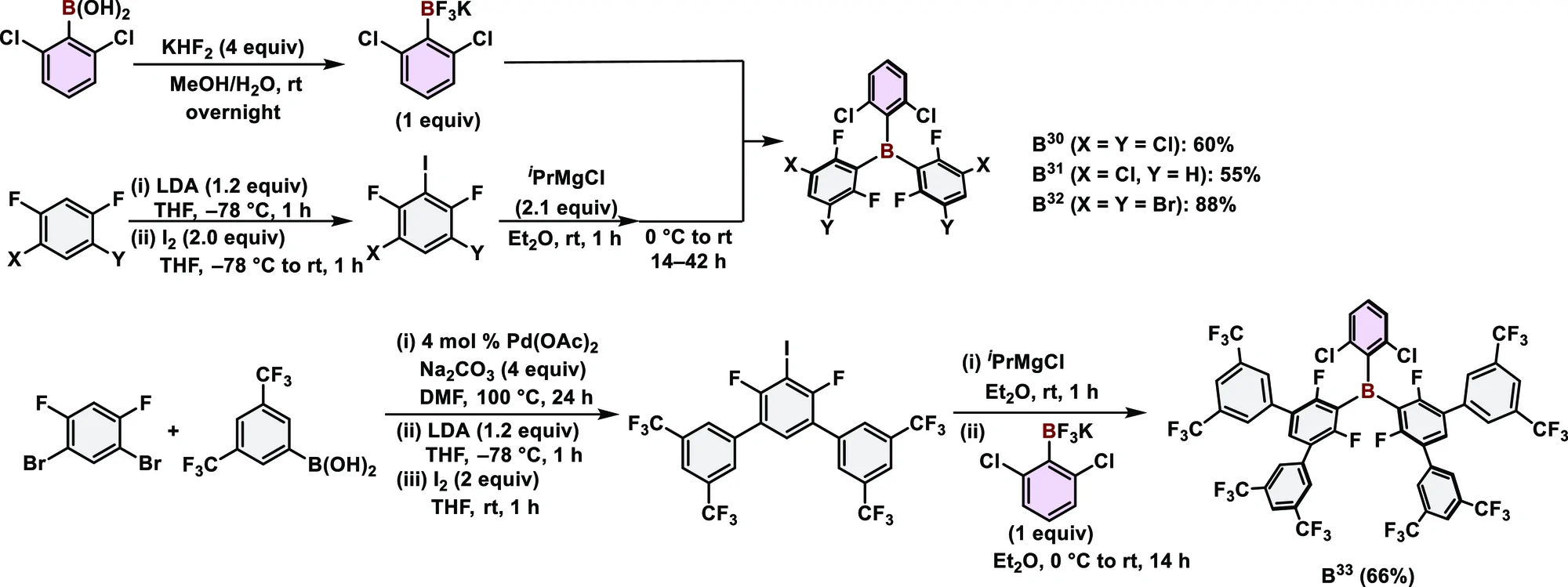
Synthesis of **B** ^**30**^–**B** ^**33**^ (LDA, Lithium Diisopropylamide)

**Scheme 12:**
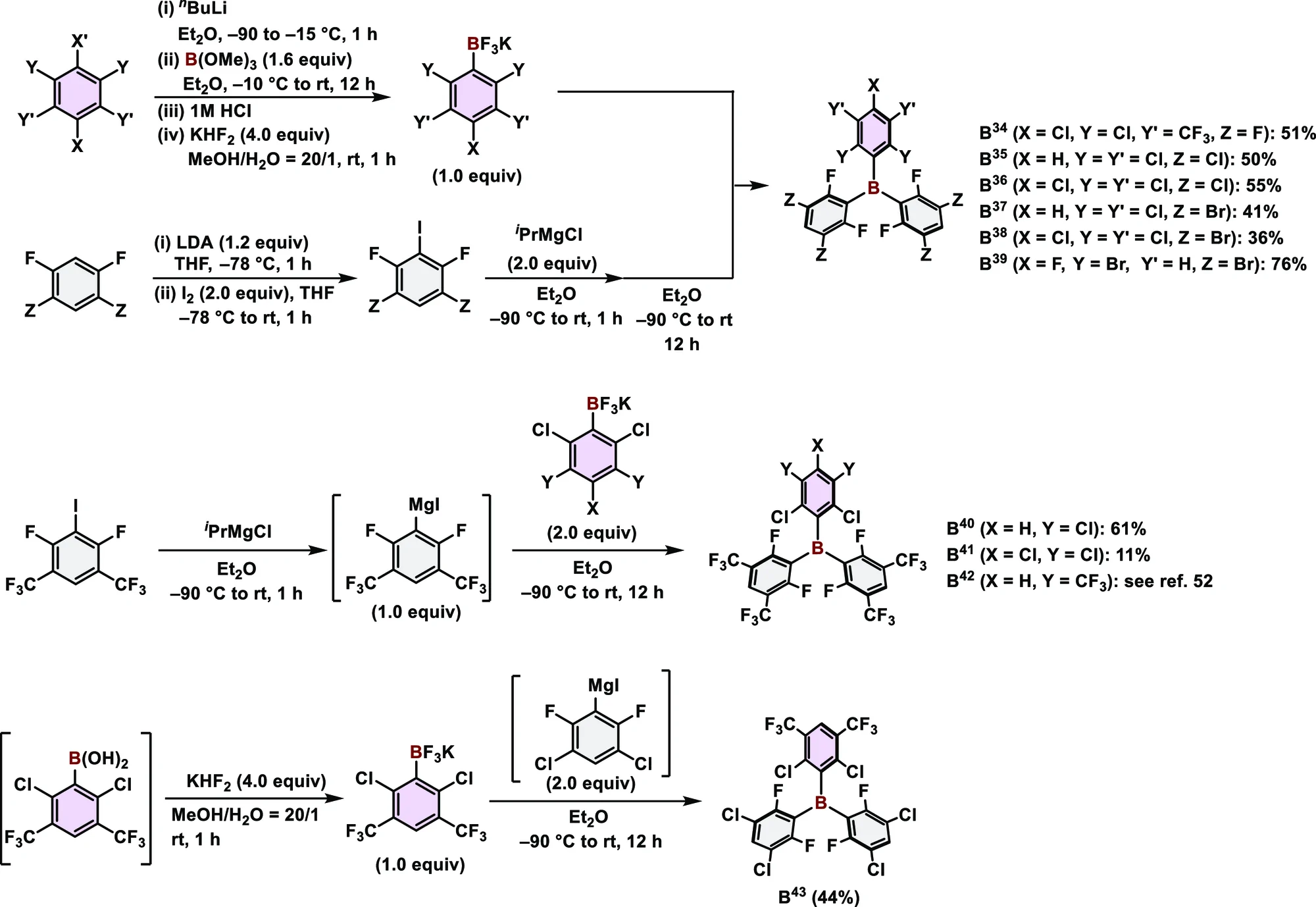
Synthesis of **B** ^**34**^–**B** ^**43**^

More sophisticated architectures have emerged through modular assembly strategies that allow precise differentiation of aryl substituents. A family of nonsymmetric triarylboranes, named “fiddler crab-type” boranes (Scheme 13), enables fine control over front and back strain, resulting in optimized Si–H activation, suppression of overreduction pathways, and generation of kinetically labile hydride intermediates.^62^ Similarly, incorporation of *o*-carboranyl substituents affords heteroleptic boranes (Scheme 14) with enhanced Lewis acidity, solubility, and resistance to deactivation, enabling efficient catalysis under demanding conditions, including crude H_2_ streams.^63,64^

**Scheme 13:**
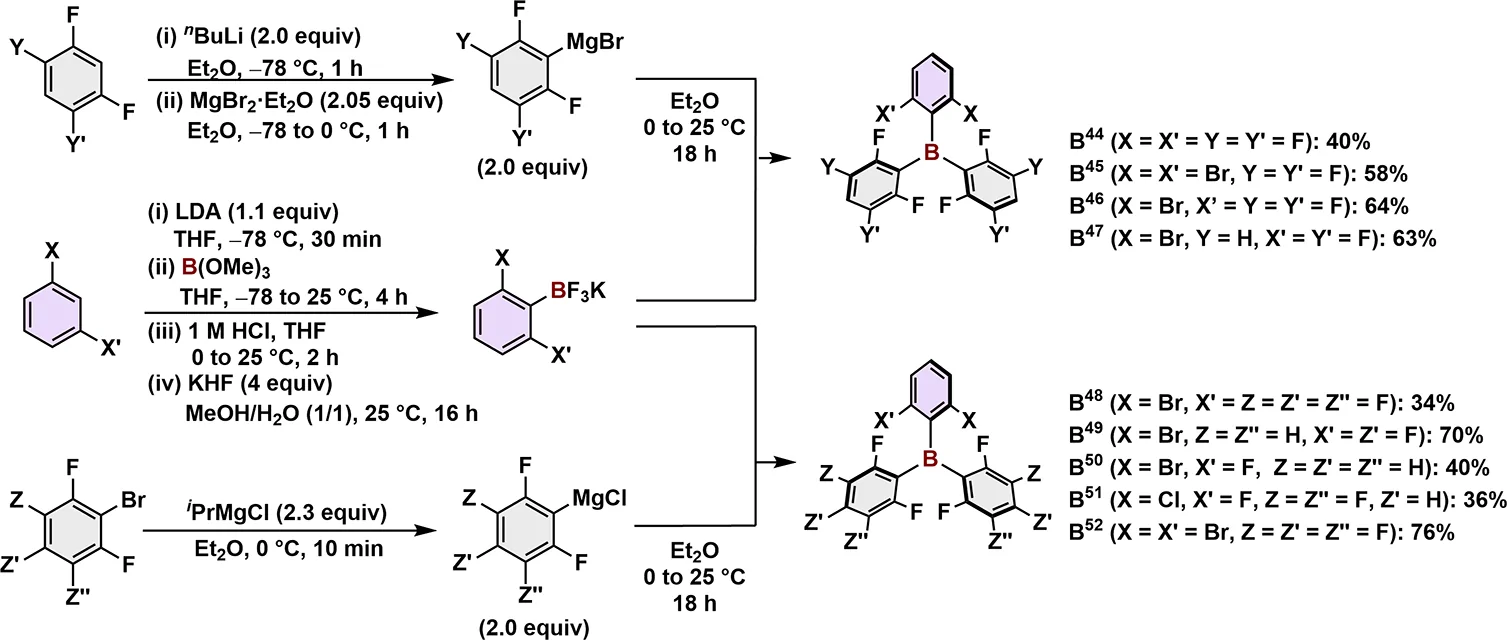
Synthesis of **B** ^**44**^–**B** ^**52**^

**Scheme 14:**
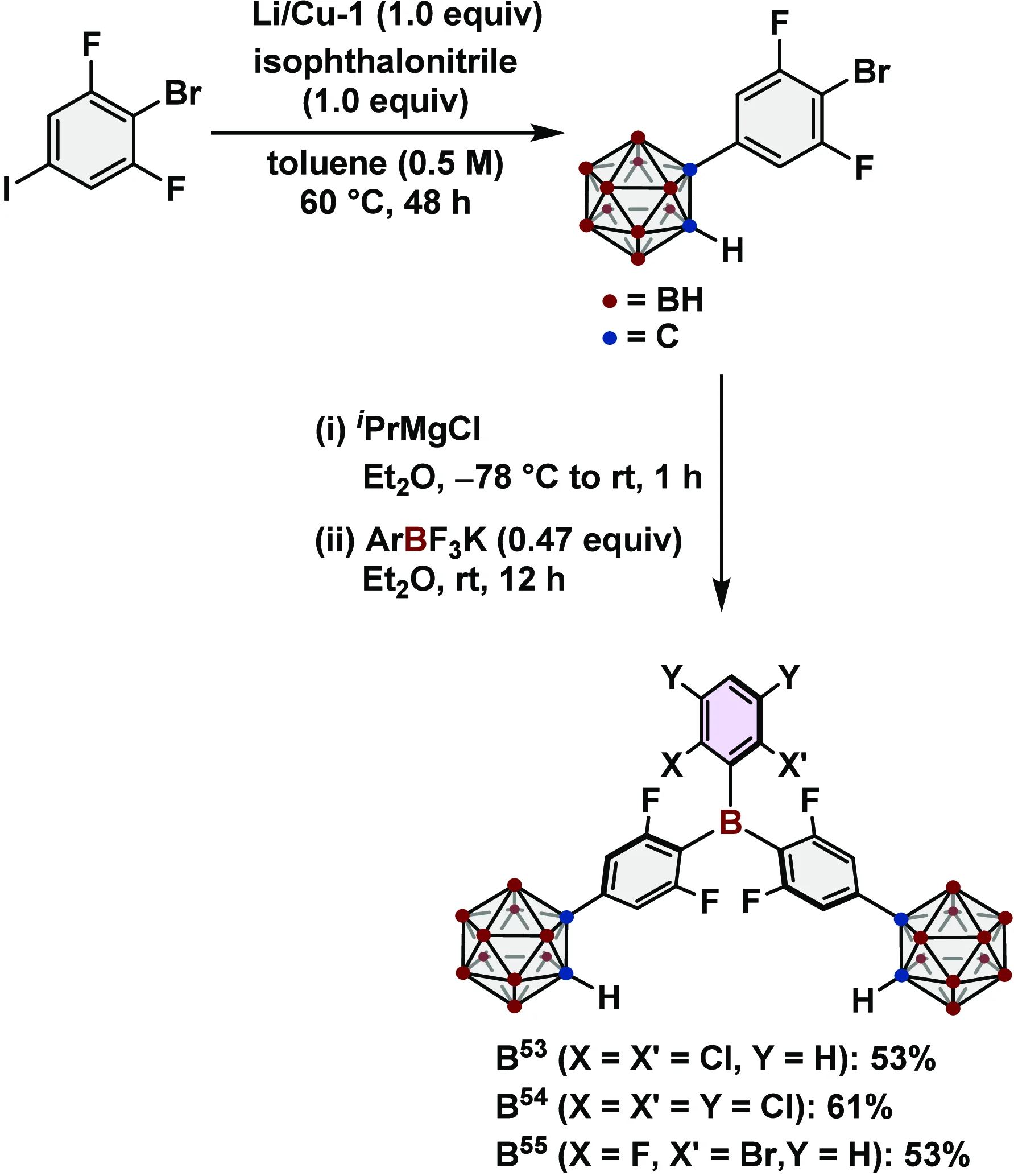
Synthesis of **B** ^**53**^–**B** ^**55**^ (**Li/Cu-1**, [Li­(thf)_4_]­[Cu­(*o*-carboran-1-yl)_2_])

The limited recyclability of molecular BAr_3_ has motivated the development of polymer-supported systems that improve stability and enable catalyst recovery. Early postfunctionalization strategies introduced boron centers into polymer backbones, thereby affording materials with controlled incorporation of Lewis acid sites.^65^ Subsequent reversible addition–fragmentation chain-transfer (RAFT)-derived copolymers enabled tunable site density but were largely confined to materials applications (Scheme 15).^66,67^

**Scheme 15:**
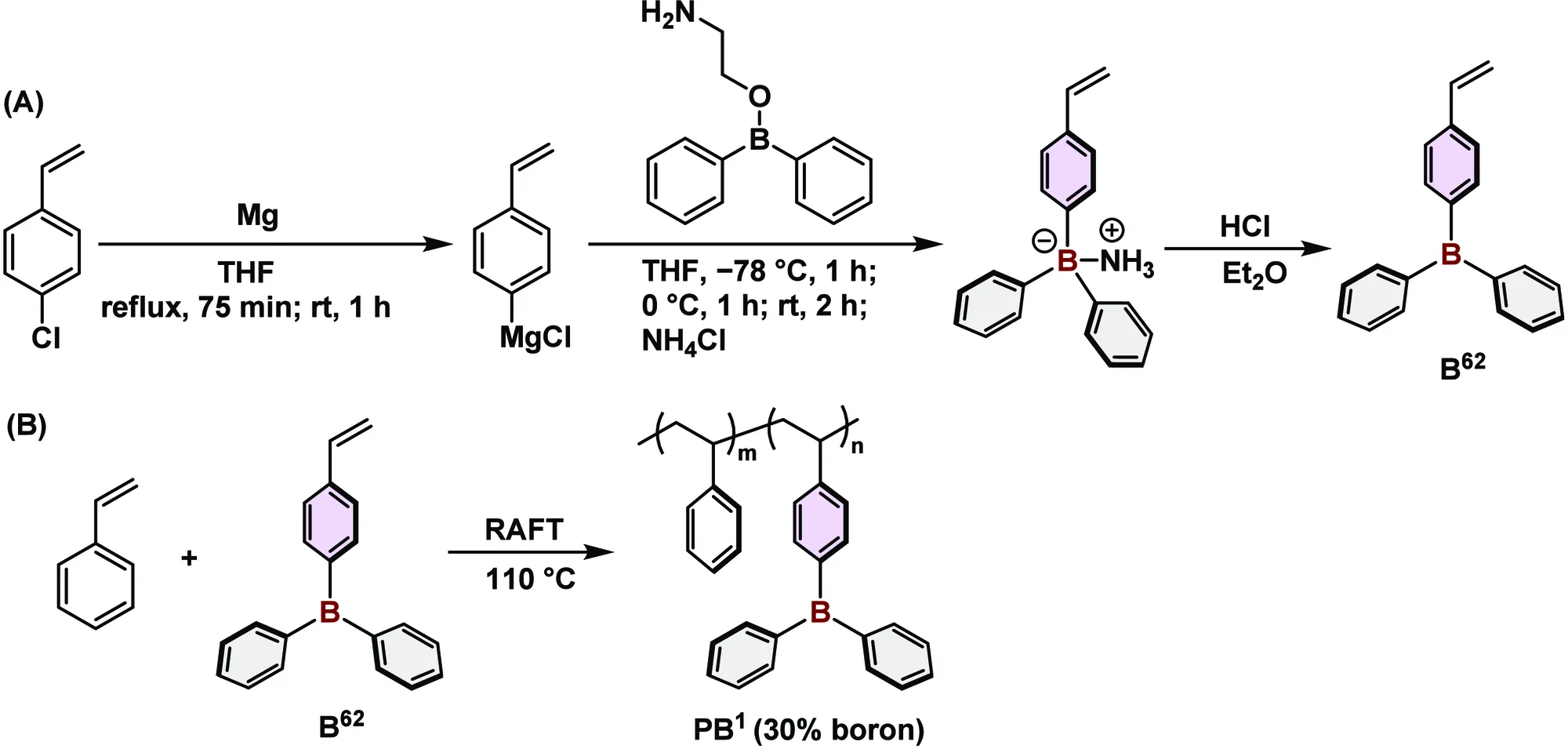
(A) Synthesis of **B** ^**62**^; (B) RAFT Copolymerization of Styrene with **B** ^**62**^ to Form **PB** ^**1**^

Recent advances have demonstrated catalytic activity in macromolecular FLP systems,^68^ while steric protection via *ortho*-substitution (Scheme 16) suppresses deactivation and preserves Lewis acidity, enabling efficient and recyclable hydrosilylation catalysis.^69^ Further refinement using prefunctionalized monomers and the ring-opening metathesis polymerization (ROMP) provides well-defined borane polymers (Scheme 17) with high activity and stability, approaching that of molecular analogues.^70^

**Scheme 16:**
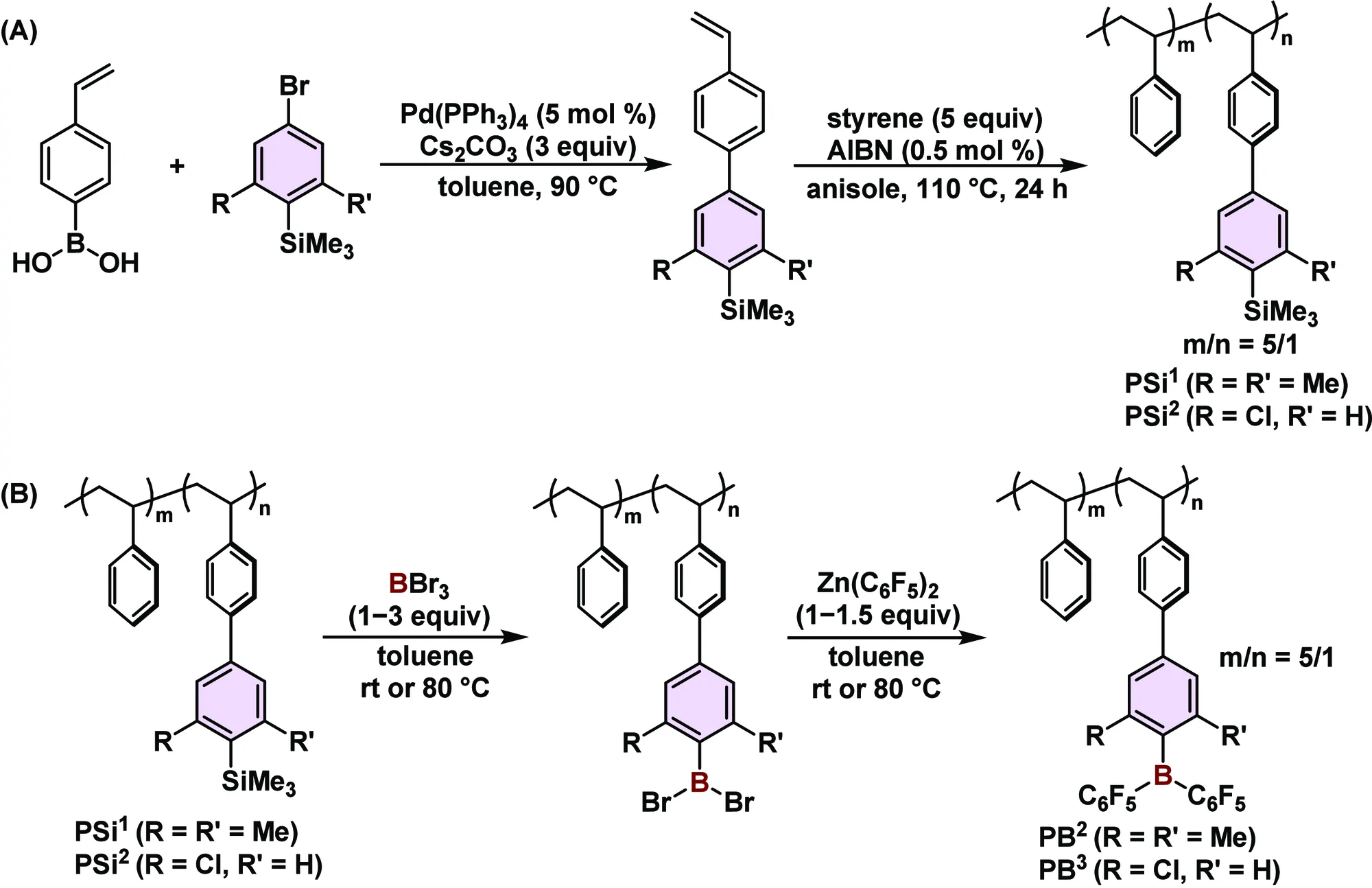
(A) Synthesis of Silane-Functionalized Polymers; (B) Conversion to Arylborane-Functionalized Polymers

**Scheme 17:**
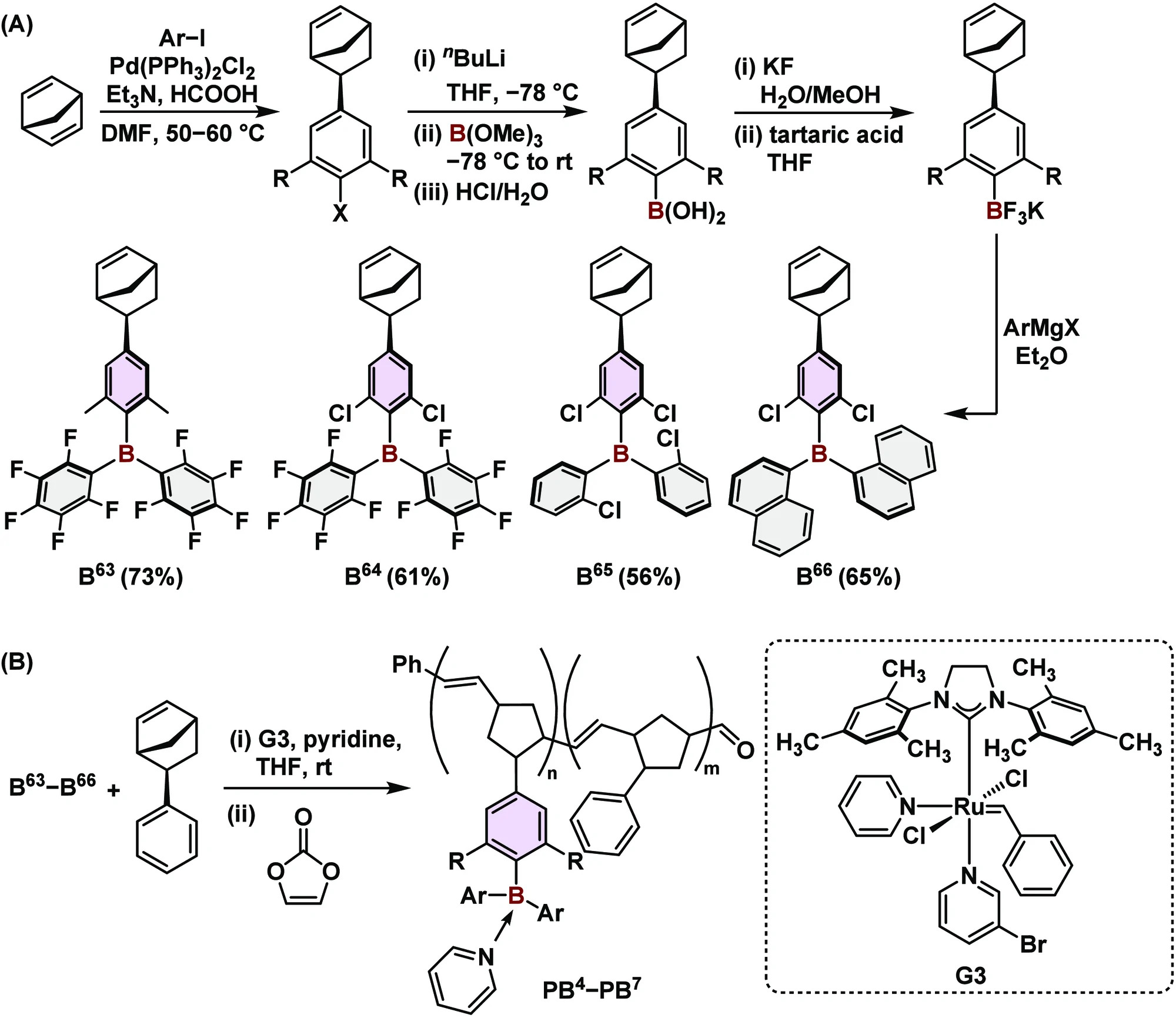
(A) Synthesis of **B** ^**63**^–**B** ^**66**^ and (B) Their ROMP Polymerization

## 3 Triarylborane-Catalyzed Transformations Using Molecular Hydrogen

### 3.1 Transformation Reactions Using Pure H_2_

Since the seminal discoveries by Stephan and Erker, the heterolytic cleavage of H_2_ by combinations of Lewis bases and BAr_3_ (e.g., **B** ^**1**^) has driven the extensive development of metal-free hydrogenation chemistry.^71–77^ These systems operate through FLP activation of H_2_, generating reactive borohydride species that enable the reduction of a wide range of unsaturated substrates.

A major limitation of early borane-catalyzed hydrogenation was its sensitivity to moisture, as strong Lewis bases promote the formation of irreversible H_2_O–borane adducts and catalyst deactivation. Soós and co-workers addressed this challenge by structural modification of BAr_3_ (**B** ^**28**^ and **B** ^**29**^), in which strategic fluorine-to-chlorine substitution destabilizes H_2_O–borane adducts while maintaining efficient H_2_ activation (Scheme 18).^52^ This design enables reductive alkylation of relatively basic amines with carbonyl compounds even under open-air conditions, establishing proximal back-strain engineering as a key principle for improving catalyst robustness.^78–81^

**Scheme 18:**
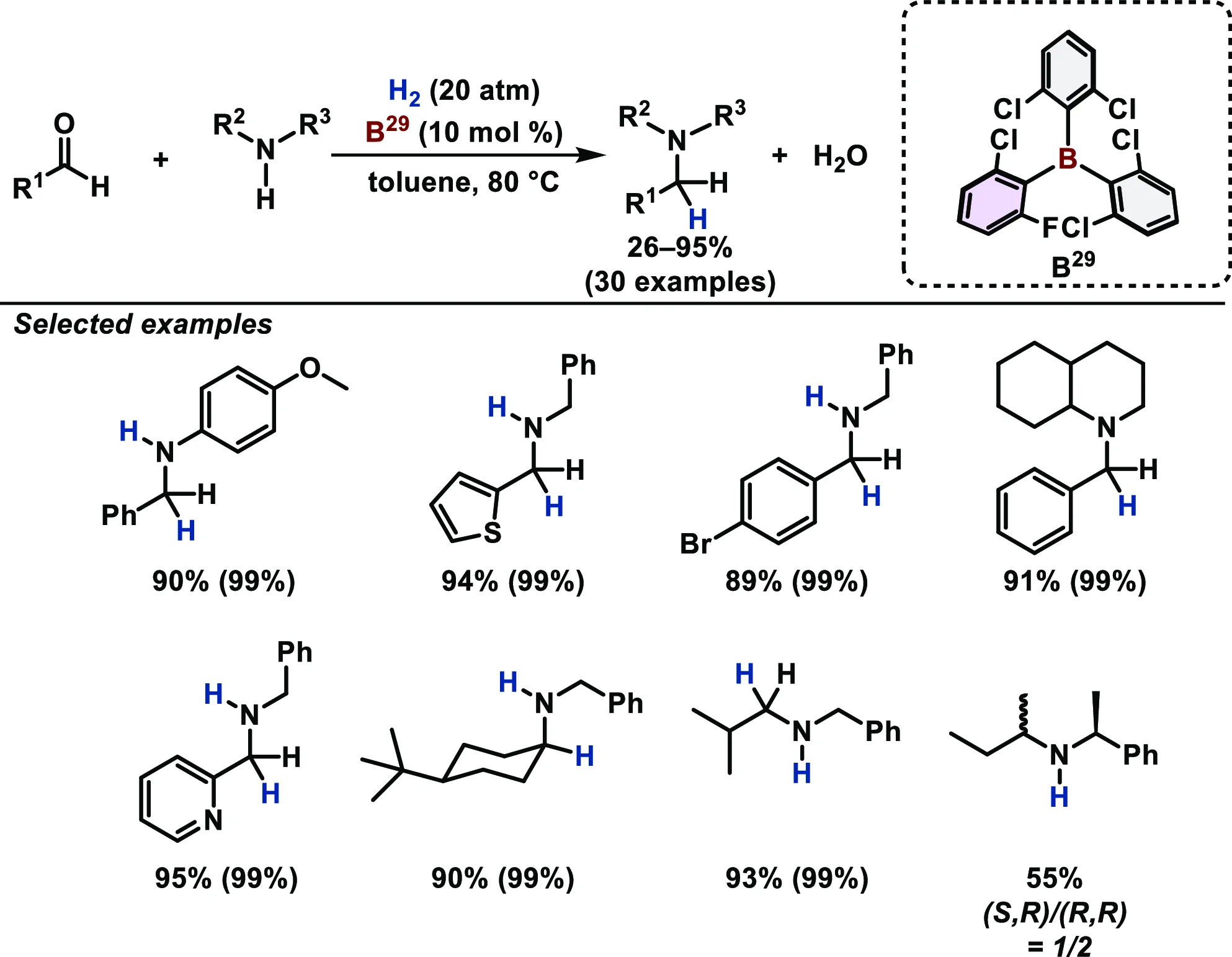
**B** ^**29**^-Catalyzed Reductive Amination of Carbonyl Compounds Using H_2_ as the Reductant^a^

a Isolated yields (conversions) are shown.

Borane design has also enabled hydrogenation of highly functionalized substrates. Hoshimoto and co-workers developed a tandem catalytic system for the reductive alkylation of aniline derivatives, in which Soós’ borane (**B** ^**56**^) catalyzed both imine formation and FLP-mediated hydrogenation steps (Scheme 19).^82^ Kinetic studies support rate-limiting H_2_ cleavage by a borane/solvent FLP, and the system exhibits broad tolerance toward functional groups such as carboxyl, hydroxyl, amide, and sulfonamide, with H_2_O as the sole byproduct.

**Scheme 19:**
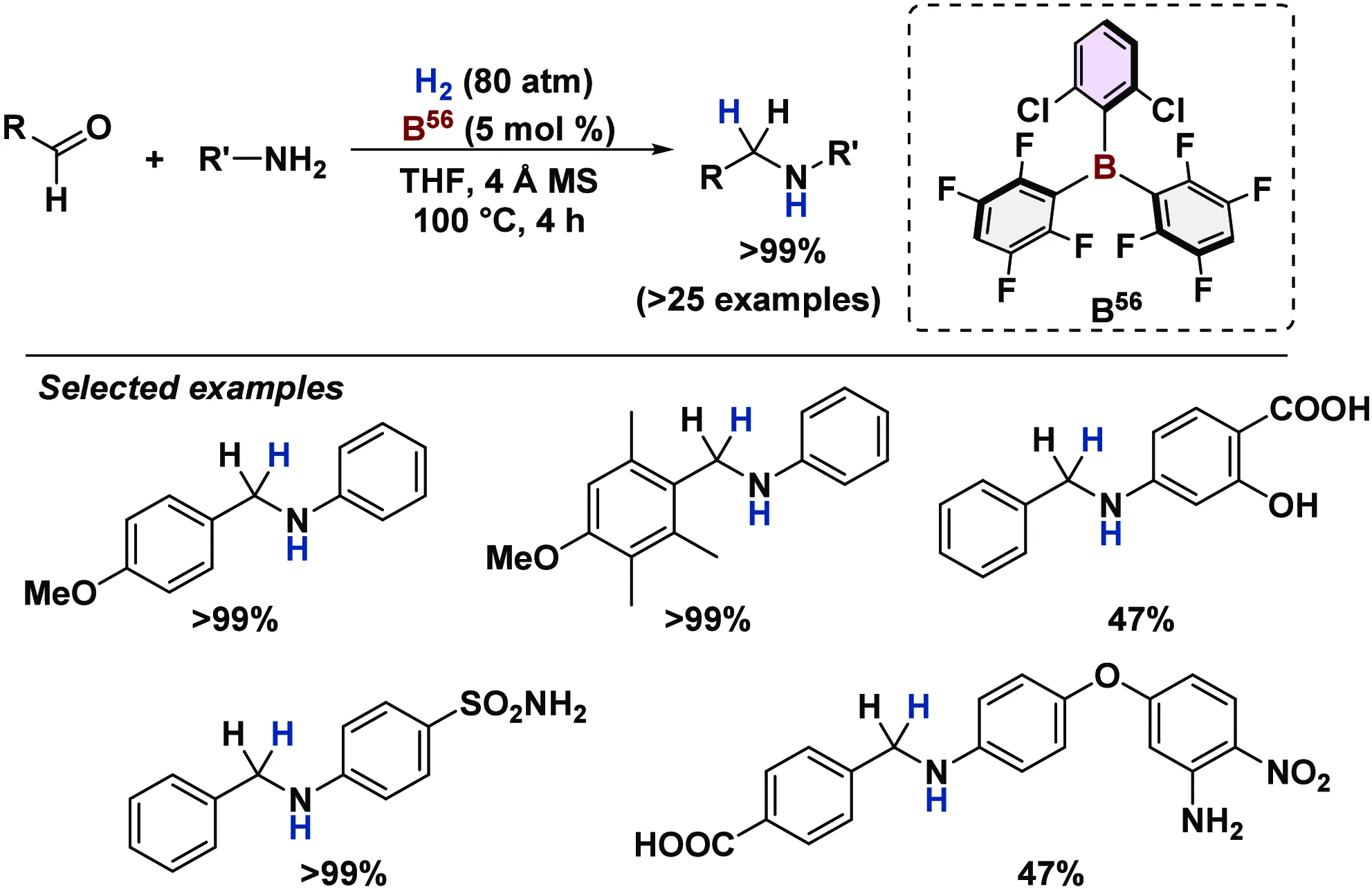
**B** ^**56**^-Catalyzed Reductive Alkylation of Amines with Aldehydes with H_2_

Expansion to structurally complex substrates has further demonstrated the versatility of borane-catalyzed hydrogenation. Hydrogenation of aza-Morita–Baylis–Hillman adducts proceeds with high *syn* diastereoselectivity via FLP-mediated H_2_ activation involving ammonium/borohydride ion pairs, followed by 1,4-addition and prototropic rearrangement (Scheme 20).^83^ In parallel, hydrogenation of amides—historically challenging due to their substantial stability—has been achieved through activation strategies that decouple substrate activation and reduction.^42,84,85^ Oxalyl chloride–mediated activation combined with halide-assisted FLP systems enables efficient reduction of amides to amines under relatively mild conditions (Scheme 21).^86^

**Scheme 20:**
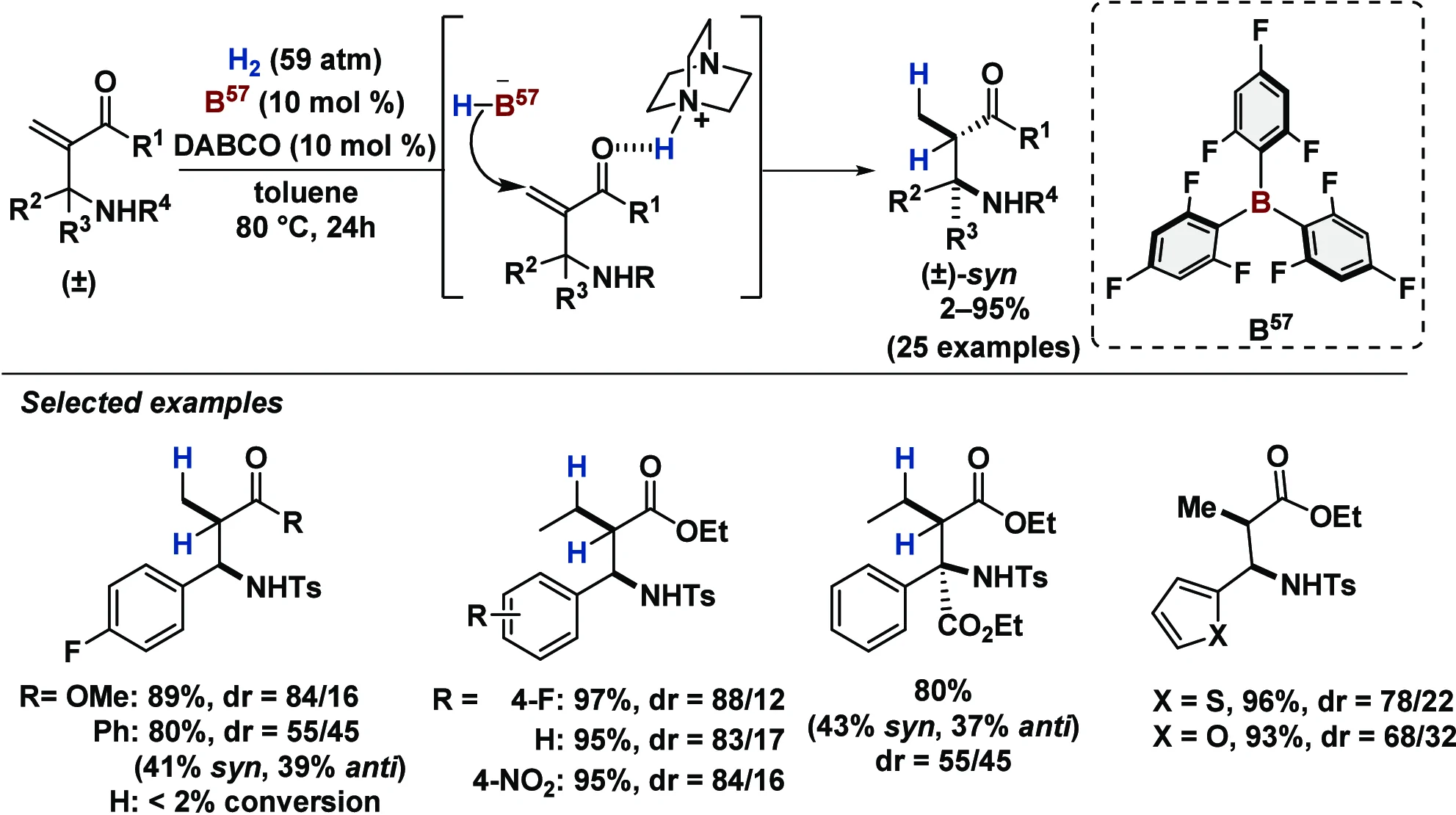
**B** ^**57**^-Catalyzed Hydrogenation of Aza-Morita–Baylis–Hillman Adducts under H_2_ Conditions

**Scheme 21:**
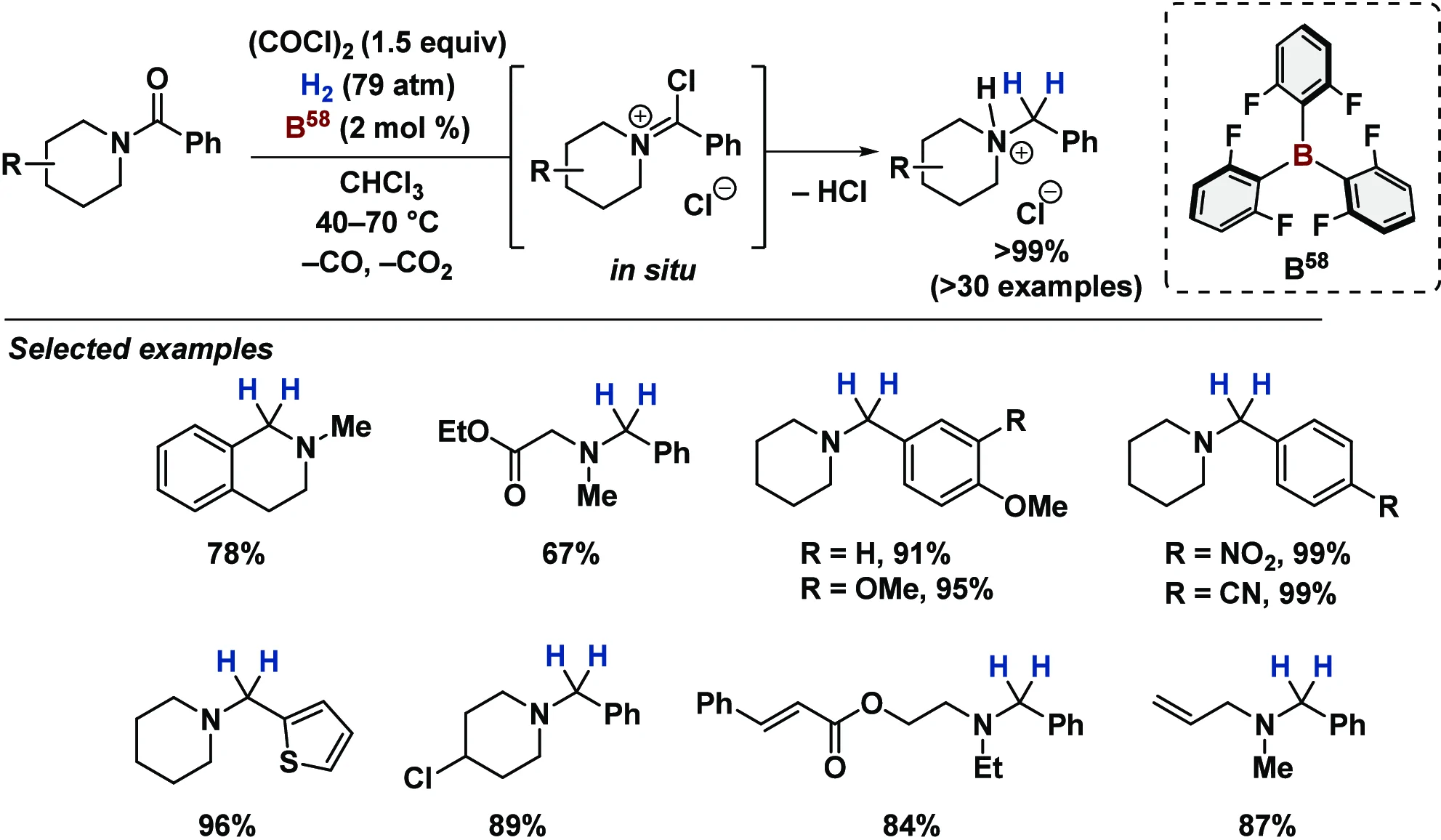
**B** ^**58**^-Catalyzed Reduction of Amides Using H_2_

Dearomatization chemistry represents a major advance in H_2_-based borane catalysis. Reduction of some pyridine derivatives bearing bulky substituents at the 2-position has been enabled through hydroboration/hydrogenation cascades that attenuate Lewis basicity prior to H_2_ activation (Scheme 22).^87^ These systems efficiently provide access to functionalized piperidines.^88–90^ Similarly, hydrogenation of indoles was achieved using the remote-back-strain-engineered **B** ^**32**^, which suppresses protodeboronation and enables high turnover numbers via dynamic switching of the Lewis basic site during H_2_ activation (Scheme 23).^91^

**Scheme 22:**
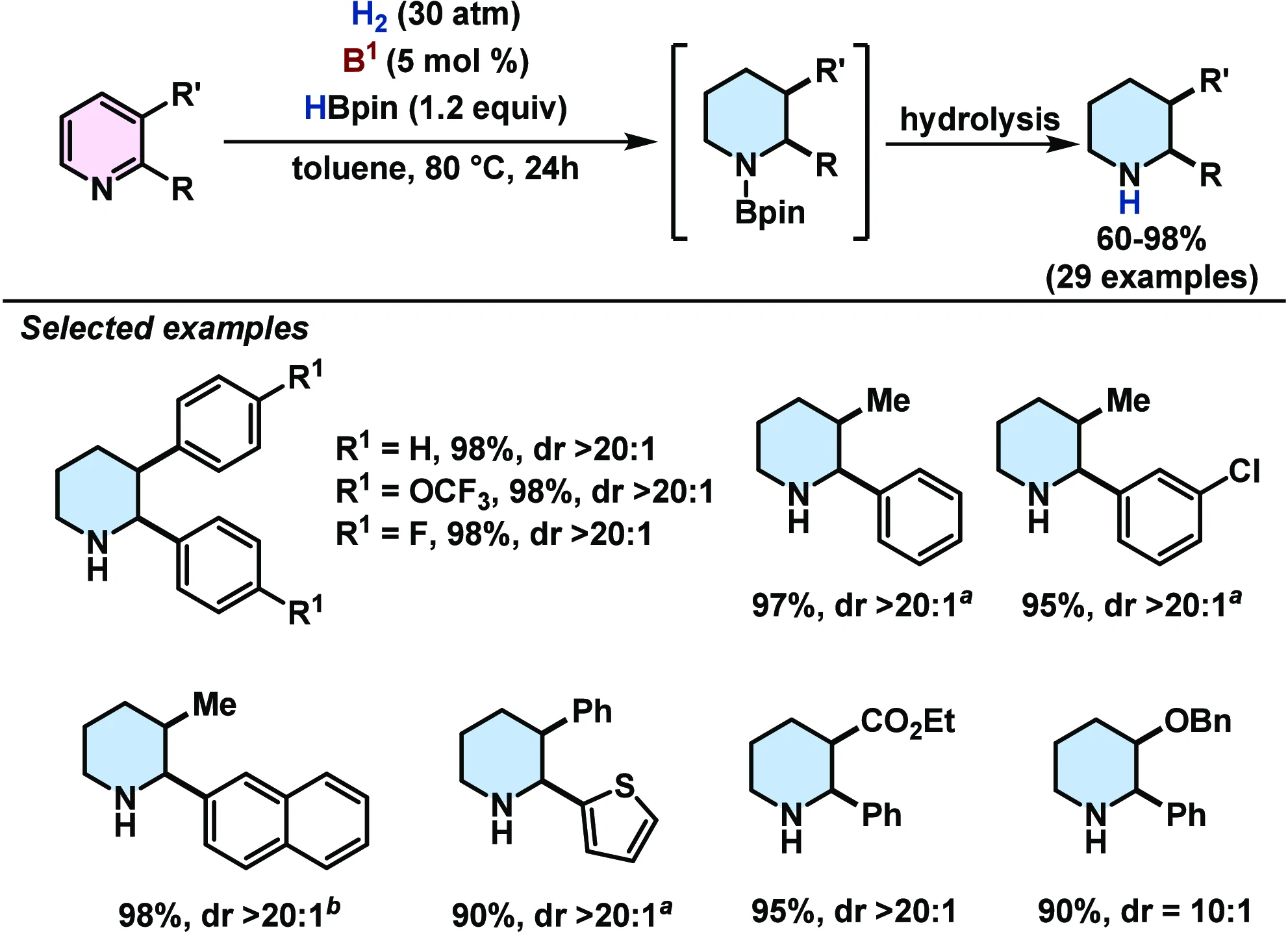
**B** ^**1**^-Catalyzed Reduction of 2,3-Disubstituted Pyridines with H_2_

a 90 °C.
b 3 mL of toluene.

**Scheme 23:**
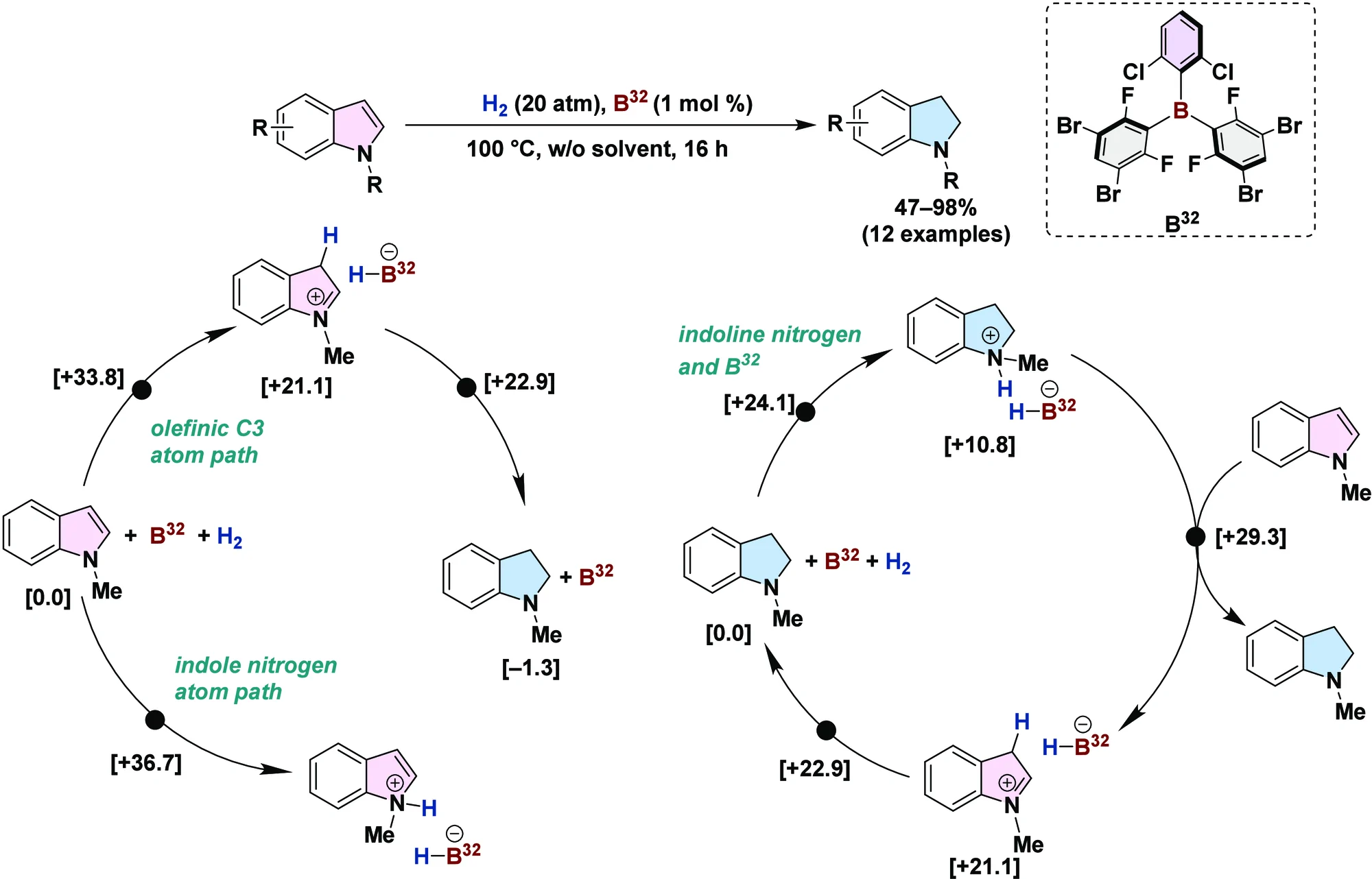
Hydrogenation of N-Substituted Indoles with **B** ^**32**^ under H_2_ Atmosphere; Relative Gibbs Free Energies [kcal mol^–1^] with Respect to [Indole (or Indoline) + **B** ^**32**^ + H_2_] Are Also Shown

Catalyst design has also been advanced through data-driven approaches. Screening of structurally diverse boranes identified **B** ^**40**^ as an optimal catalyst for reductive alkylation of amino acids and peptides, in which activity correlates with parameters governing H_2_ activation and catalyst stability rather than with simple Lewis acidity.^61^ These systems operate efficiently in 4-methyltetrahydropyran (MTHP) and exhibit broad substrate scope, enabling environmentally less-harmful hydrogenation of highly functionalized molecules (Scheme 24).

**Scheme 24:**
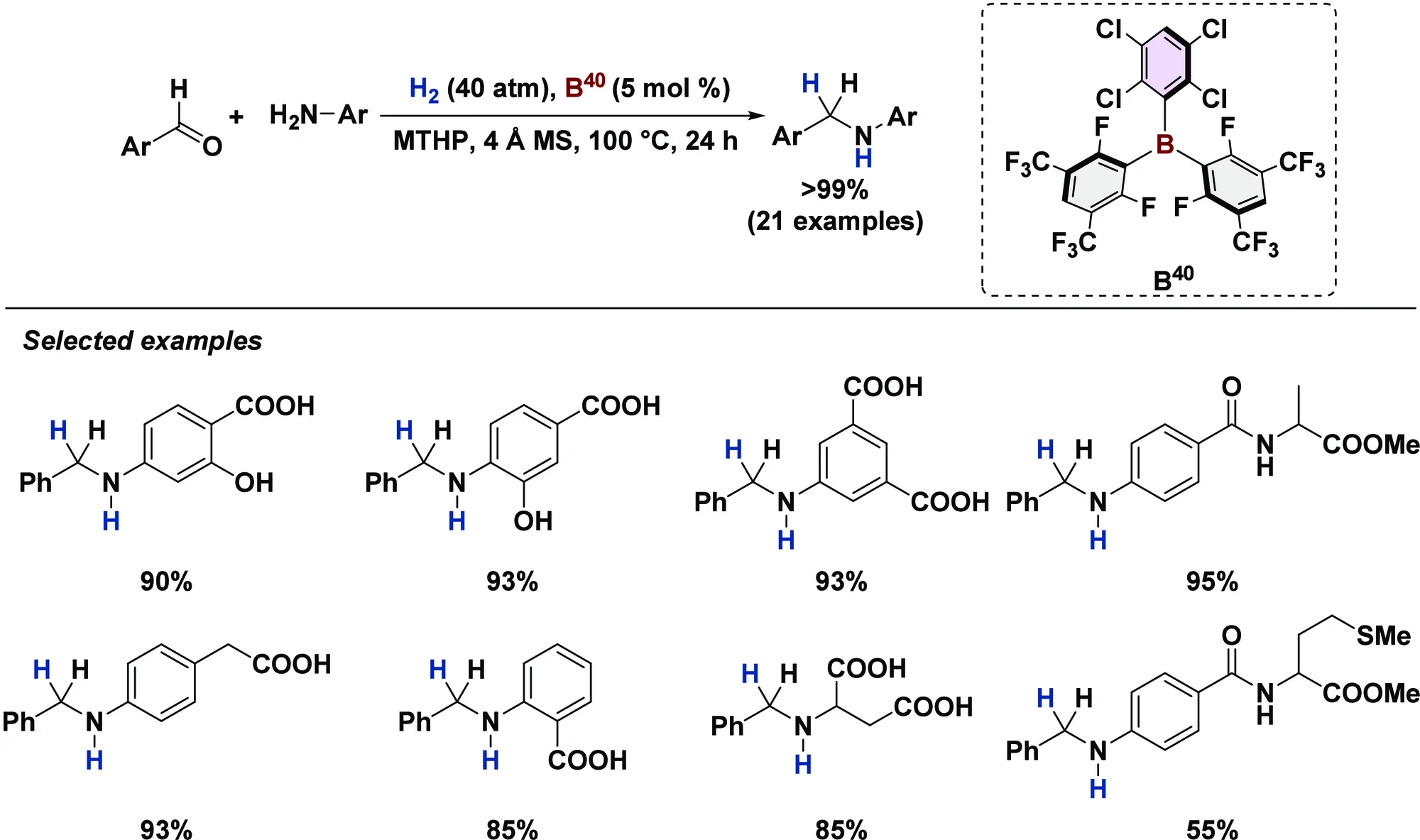
**B** ^**40**^-Catalyzed Reductive Alkylation of Aniline-Derived Amino Acids and Peptides in the Presence of H_2_ (MTHP, 4-Methyltetrahydropyran)

Borane-catalyzed hydrogenation has been extended to various unsaturated compounds, including polycyclic *N*-heterocycles and sterically hindered olefins.^92–94^ Such transformations proceed via carbocation-type reduction pathways enabled by strong Lewis acidity and optimized Lewis base partners, providing access to saturated heterocycles with high diastereoselectivity.^94,95^

Asymmetric hydrogenation was achieved using chiral FLP systems, where stereocontrol arose from concerted proton–hydride delivery and noncovalent interactions in the transition states. Both chiral Lewis base-derived FLPs and relay catalysis strategies, combining achiral boranes with chiral Brønsted acids, enabled enantioselective reduction of enones,^96^ chromones,^97^ and related substrates,^98,99^ as well as stereodivergent synthesis of complex frameworks (Scheme 25).^100^

**Scheme 25:**
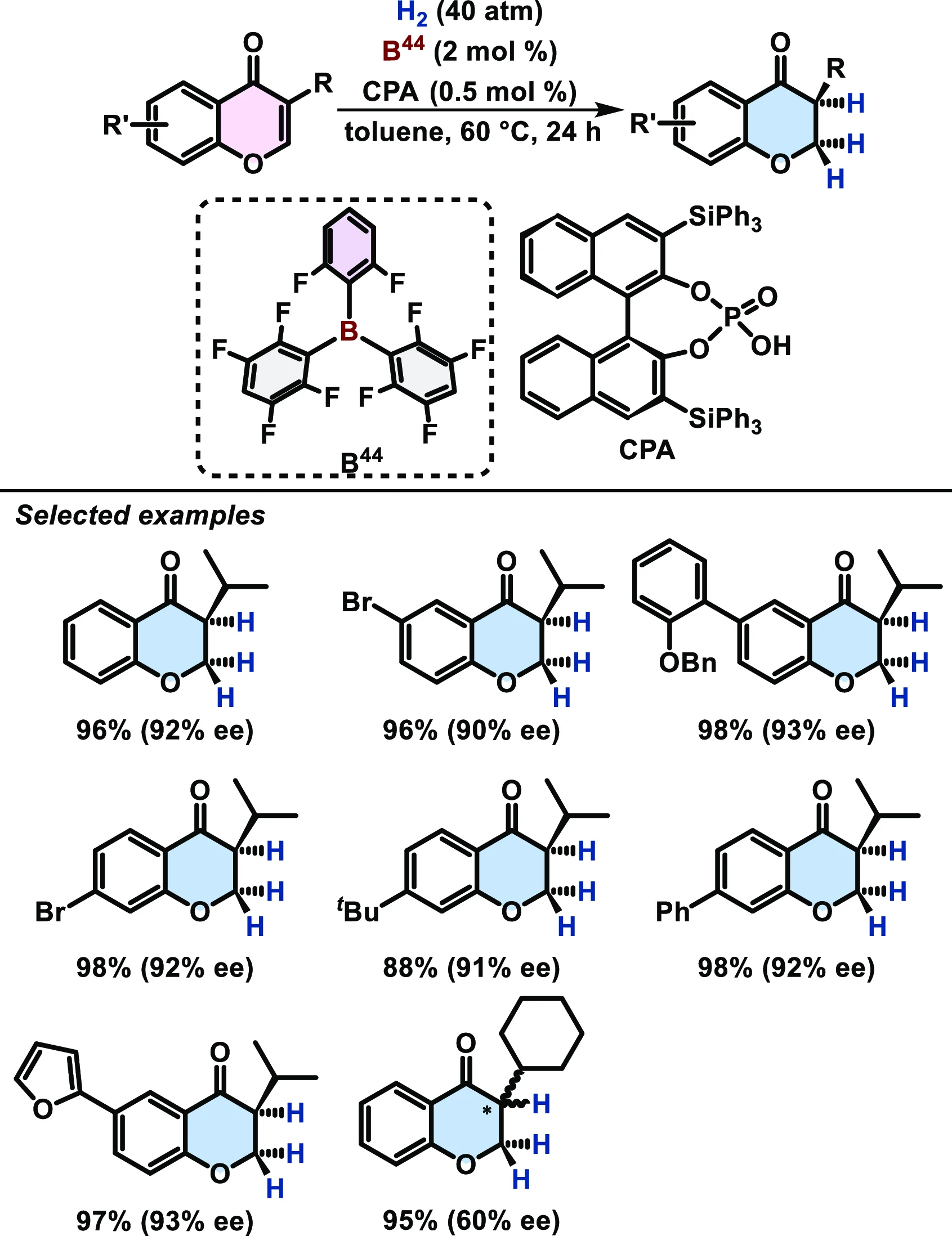
**B** ^**44**^-Catalyzed Asymmetric Hydrogenation of Chromones

Mechanistic studies provided detailed insight into H_2_ activation and reduction pathways. Computational analyses reveal that heterolytic H_2_ cleavage proceeds via a concerted yet asynchronous transition state, in which hydride transfer to boron precedes proton transfer to the Lewis base.^101^ Subsequent hydride transfer from triarylborohydride intermediates is typically stereodetermining and governed by Curtin–Hammett control, with reactive conformers stabilized by steric and noncovalent interactions.^102,103^

Beyond classical hydrogenation, unconventional FLP systems have emerged. Ionic borane–carbonate adducts enable metal-free reduction of CO_2_ to formate via H_2_ activation,^104^ while related borane/base systems mediate reduction of Si–X bonds^105^ and hydrogenative cyclization reactions (Scheme 26).^106^ These transformations highlight the ability of borane catalysts to orchestrate coupled reduction–bond-forming processes under mild, metal-free conditions.

**Scheme 26:**
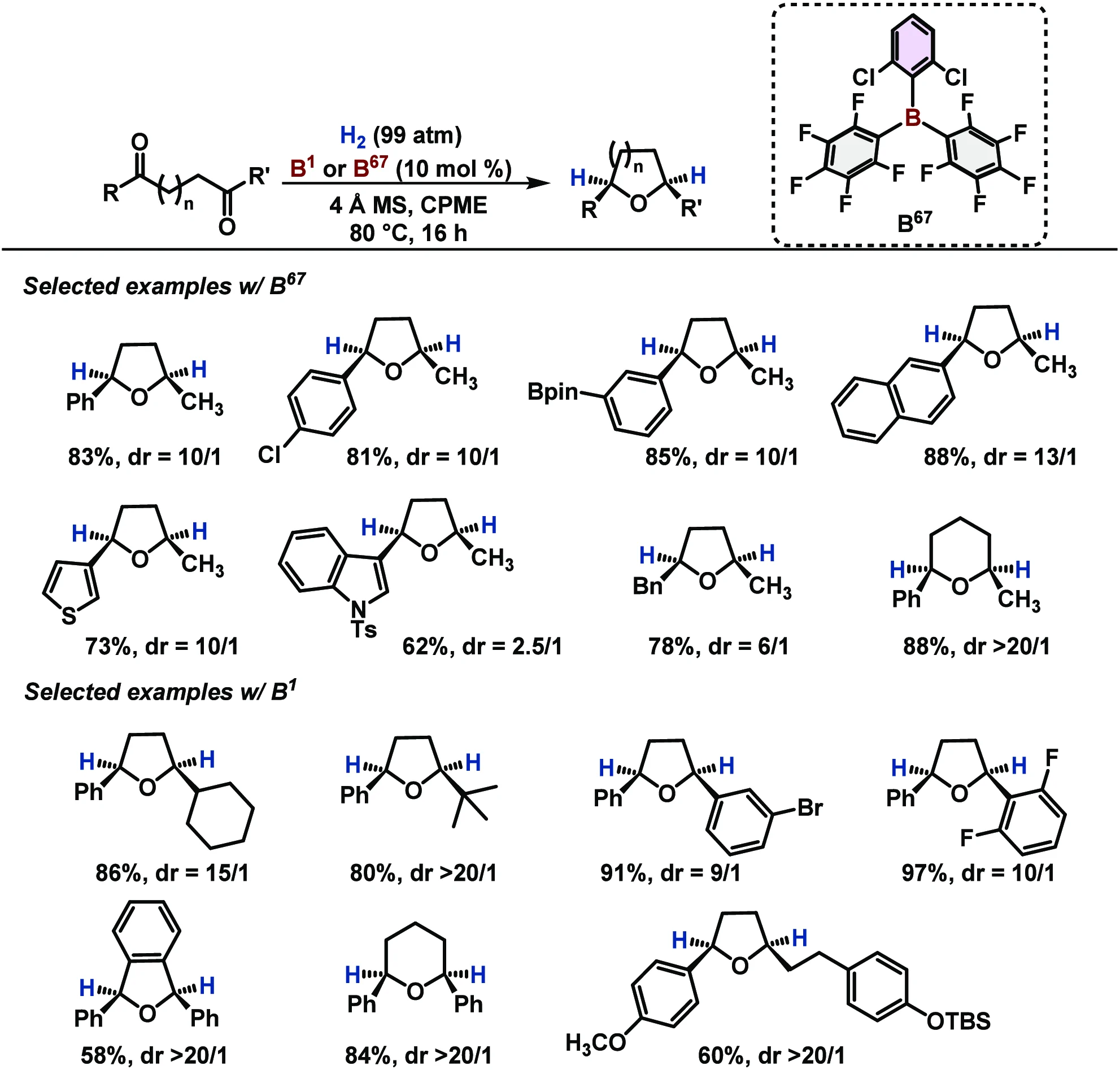
**B** ^**1**^- or **B** ^**67**^-Catalyzed Reductive Cyclization of Diketones (CPME, Cyclopentyl Methyl Ether)

### 3.2 Transformation Reactions Using Gaseous Mixtures Including H_2_

The use of gaseous mixtures containing H_2_ and common contaminants (e.g., CO, CO_2_, and CH_4_) as reductants in borane-catalyzed hydrogenation represents a significant advance toward industrially relevant conditions. The concept of using contaminated H_2_ streams containing CO, CO_2_, and/or gaseous hydrocarbons, introduced by Hoshimoto and co-workers, established crude hydrogen (or raw syngas) as a viable reductant for main-group catalysis.^107^ Subsequent studies demonstrated both experimental implementation and mechanistic understanding of main-group-catalyzed H_2_ purification and utilization under mixed-gas environments.^60^ Notably, the tolerance of appropriately designed triarylboranes toward impurities such as CO, CO_2_, and H_2_O represents a significant practical advantage over many transition-metal hydrogenation catalysts, which are often susceptible to poisoning or irreversible deactivation under comparable conditions. This enhanced impurity tolerance enables efficient hydrogenation using contaminated H_2_ streams and highlights the potential of borane catalysts for practical applications, where extensive gas purification is undesirable or economically prohibitive.

A central challenge in such systems arises from the increased reactivity of BAr_3_ toward CO and CO_2_ compared with H_2_; whereas CO reversibly binds to the Lewis acidic boron center,^108,109^ CO_2_ fixation and the subsequent decomposition of BAr_3_ species seriously compromise the catalytic performance. This limitation was addressed through the remote back-strain strategy, in which steric repulsion and noncovalent interactions between *meta* substituents modulated deformation energy and effective Lewis acidity without significantly altering the intrinsic electronic properties. This approach enabled the development of boranes (e.g., **B** ^**30**^ and **B** ^**32**^) that retain efficient H_2_ activation and catalytic hydrogenation activity even under H_2_/CO/CO_2_/CH_4_ (1/1–5/1–5/1 molar ratio) mixtures.^60^ Notably, quinoline hydrogenation proceeded with activity and selectivity comparable to those observed under pure H_2_, demonstrating that rationally engineered boranes can directly utilize crude hydrogen streams (Scheme 27).

**Scheme 27:**
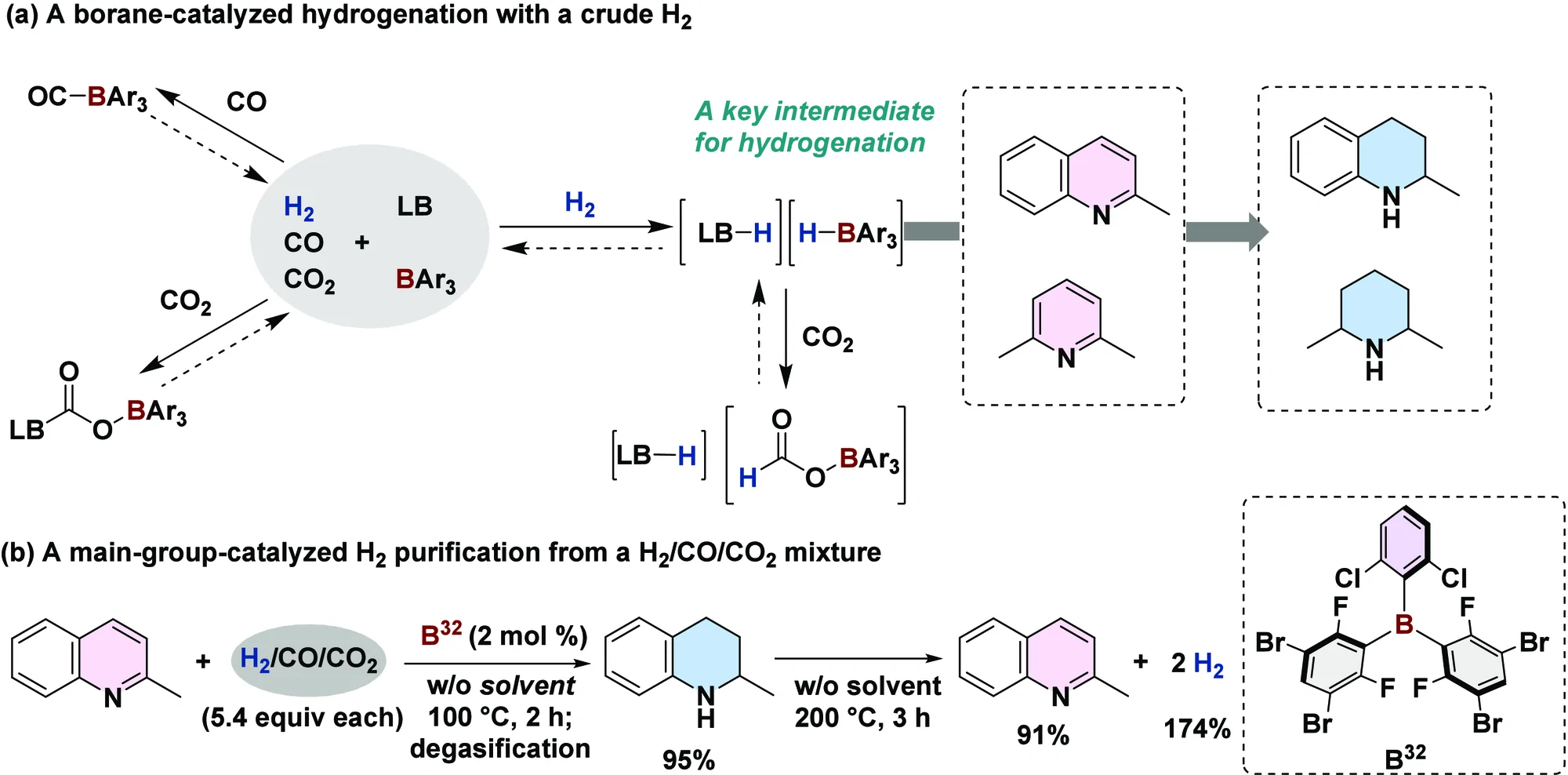
(a) Catalytic Hydrogenation of 2-Methylquinoline and 2,6-Lutidine Using Boranes under Crude H_2_; (b) **B** ^**32**^-Catalyzed H_2_ Purification from a H_2_/CO/CO_2_ Mixture

This design principle was subsequently generalized to a broader range of catalysts. Related boranes (e.g., **B** ^**7**^ and **B** ^**8**^) exhibit enhanced activity relative to **B** ^**1**^ for quinoline hydrogenation under mixed-gas conditions.^52^ Extension to carbonyl substrates further demonstrated that remote backstrain-engineered boranes (e.g., **B** ^**10**^ and **B** ^**30**^) could mediate selective heterolytic H_2_ cleavage in complex gas mixtures, including industrially relevant compositions containing CO, CO_2_, and CH_4_ after brief drying with molecular sieves.^53^ These systems enabled efficient hydrogenation of both aromatic and aliphatic aldehydes and ketones (Scheme 28).

**Scheme 28:**
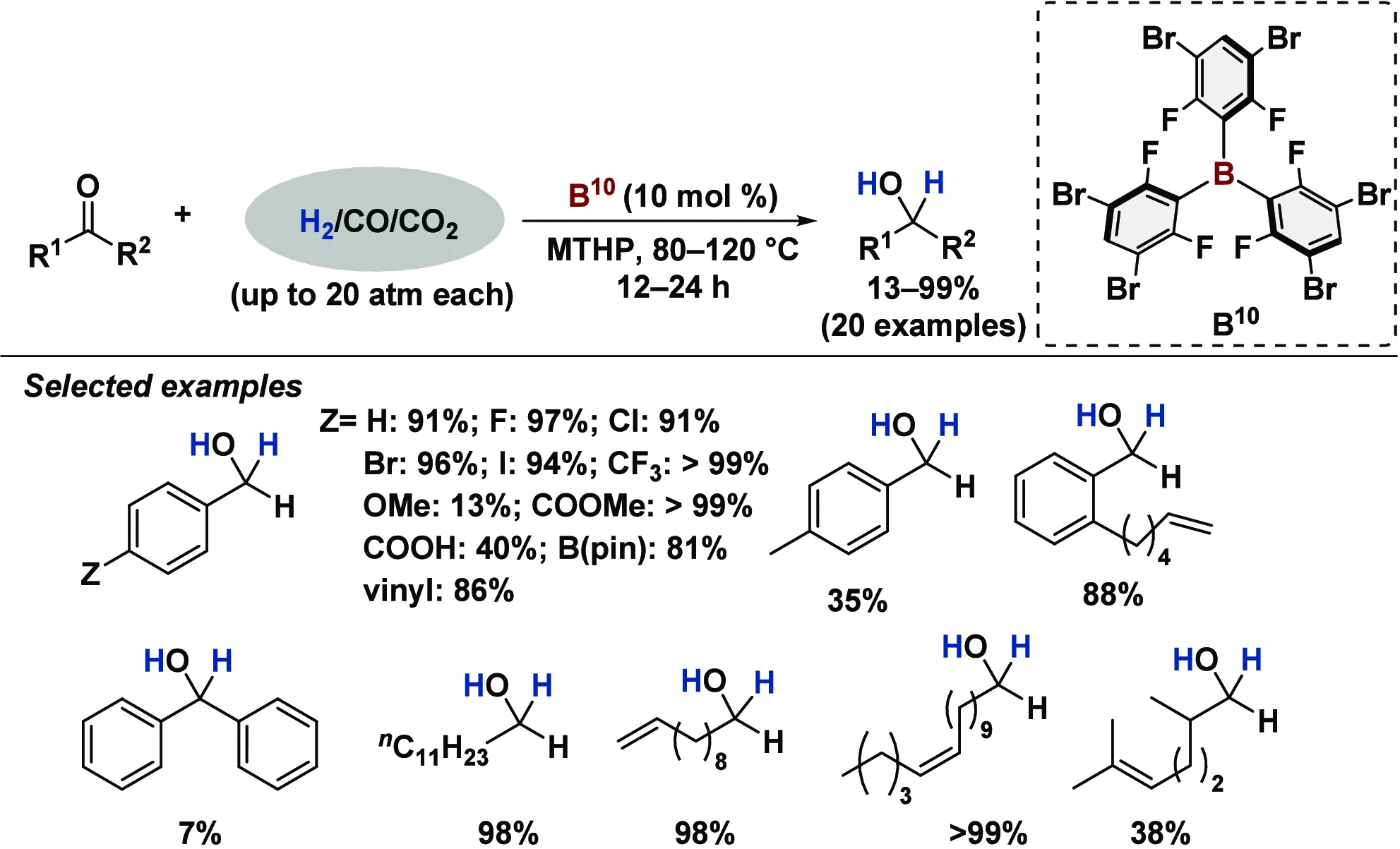
**B** ^**10**^-Catalyzed Hydrogenation of Unsaturated Carbonyls Using Crude H_2_ (MTHP, 4-Methyltetrahydropyran)

Mechanistic studies have provided deeper insights into these multicomponent systems. Comprehensive experimental investigations, combined with artificial force-induced reaction (AFIR) analysis, revealed the interplay among gas-phase composition, borane structure, and catalytic turnover, offering a unified framework for understanding reactivity under realistic conditions.^14,110^

More recently, catalyst design expanded to heteroleptic BAr_3_ incorporating (*o*-carboran-1-yl)­aryl substituents (e.g., **B** ^**53**^–**B** ^**55**^). The strongly electron-withdrawing yet highly lipophilic nature of *o*-carborane moieties enhanced both the effective Lewis acidity of the boron centers and the catalyst solubility, while conferring resistance to deactivation by Lewis bases. As a result, these systems enabled solvent-free hydrogenation of 2-methylquinoline with high turnover numbers (TONs up to 2400 under H_2_/CO_2_ and 1440 under H_2_/CO/CO_2_), while maintaining activity comparable to that under pure H_2_.^63^ In addition to hydrogenation, these catalysts also promote dehydrogenation of tetrahydroquinoline derivatives and reductive alkylation of amino acid esters, further demonstrating their versatility under mixed-gas conditions.

## 4 Triarylborane-Catalyzed Transformations Using Hydrosilanes

### 4.1 Reductive Functional Group Transformations

Hydrosilanes are versatile organic reductants in borane-catalyzed transformations, offering tunable and chemoselective alternatives to H_2_. Early studies established **B** ^**1**^-catalyzed hydrosilylation of carbonyl compounds as a general platform for reduction, proceeding via an S_N_2-type transition state involving a characteristic [Si–H···B] interaction.^111–115^ While these foundational mechanistic studies defined the reactivity of borane–silane systems,^116–122^ early applications were largely confined to carbonyl reduction and closely related transformations. These earlier developments have been documented in several previous reviews.^5,123–126^ More recent developments have significantly expanded the scope of borane-catalyzed hydrosilylation to include diverse reductive functional-group transformations. In parallel, BAr_3_-catalyzed Si–H activation has enabled the development of silicon-containing materials^127–140^ and has been leveraged in emerging strategies for material degradation.^141–147^ Accordingly, this section focuses on advances from 2016 onward, during which the field has transitioned toward broadly applicable and synthetically powerful reductive methodologies.

The reduction of nitro groups represents an early extension beyond that of carbonyl chemistry. Porwal and Oestreich demonstrated efficient conversion of nitroarenes and aliphatic nitro compounds to amines using Et_3_SiH under solvent-free conditions.^148^ Enantioselective hydrosilylation has also been achieved using sterically congested binaphthyl-derived boranes in combination with trihydrosilanes, delivering up to 99% ee in the reduction of acetophenone.^149^

Borane-catalyzed systems further enable selective C–C and C–heteroatom functionalization without complete reduction. For example, hydrosilylation of α,β-unsaturated esters and amides proceeds via initial 1,4-reduction followed by borane-mediated silyl migration, affording α-silyl carbonyl compounds with high regio- and chemoselectivity under mild conditions (Scheme 29).^150^ Similarly, selective conjugate reduction of α,β-unsaturated ketones has been achieved via silylium–hydridoborate ion pairs, enabling β-selective hydride delivery while suppressing full deoxygenation.^151^

**Scheme 29:**
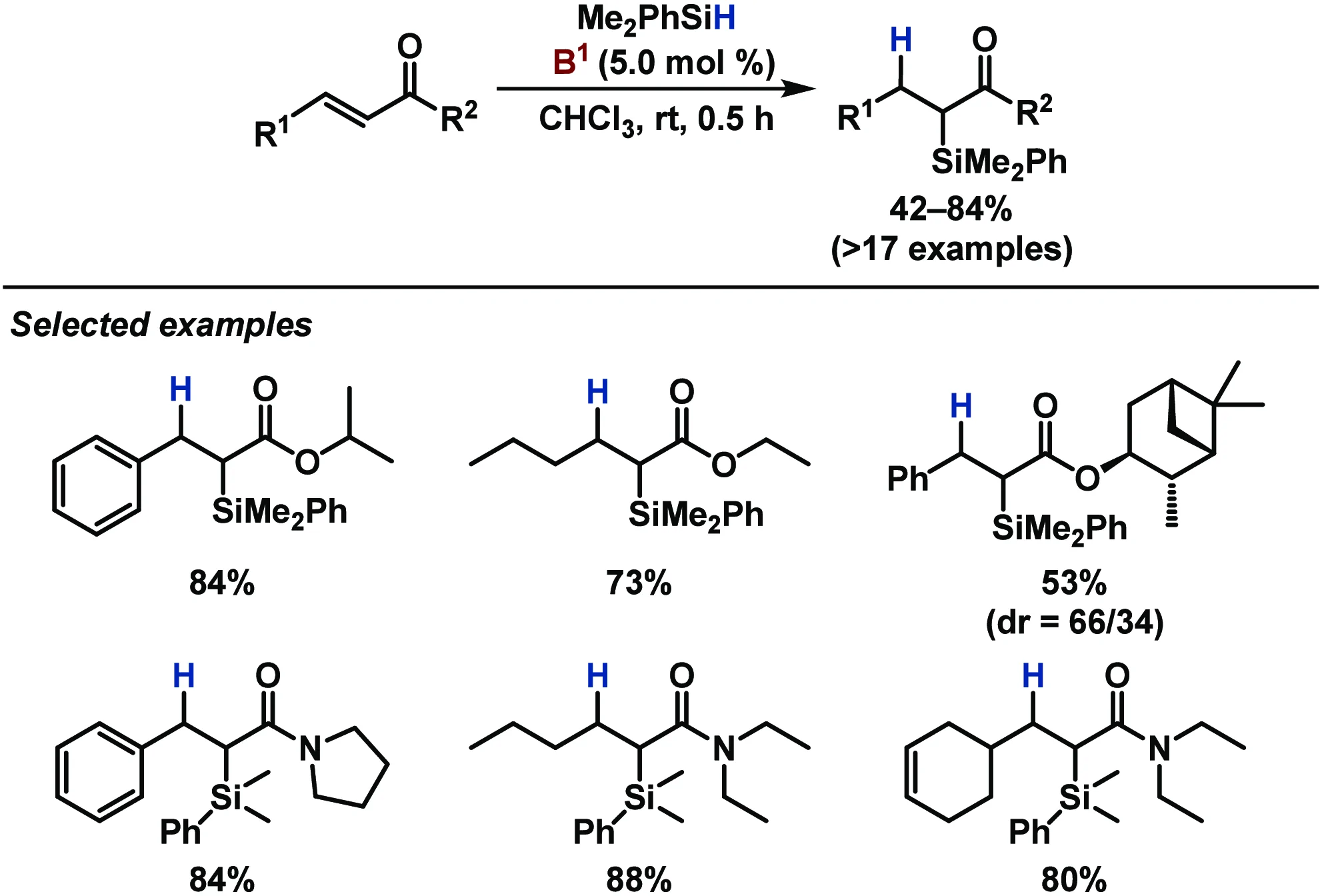
**B** ^**1**^-Catalyzed Hydrosilylation of α,β-Unsaturated Esters and Amides with Me_2_PhSiH

Control over regio- and stereoselectivity has also been demonstrated in unsaturated systems. Highly selective hydrosilylation of thioalkynes proceeds via Si–H activation to generate silylium-like intermediates, followed by nucleophilic alkyne attack to afford stereodefined alkenyl silanes.^152^ In parallel, selective hydrosilylation of terminal alkynes enables access to vinylsilanes and, with further silane incorporation, unsymmetrical geminal bis­(silanes).^153^

Reductive amination has emerged as a key transformation catalyzed by BAr_3_. Ingleson and co-workers demonstrated that **B** ^**1**^-catalyzed reductive amination can proceed efficiently under “wet” conditions using Me_2_PhSiH, overturning the assumption that boranes are irreversibly deactivated by water (Scheme 30).^154^ This reactivity arises from the reversible formation and deprotonation of H_2_O–**B** ^**1**^ adducts, thereby regenerating the active catalyst and favoring imine reduction over competing pathways. Extension to less Lewis acidic boranes (e.g., **B** ^**59**^ and **B** ^**61**^) further enables reductive amination of aldehydes and ketones with a broad range of alkylamines under operationally simple, moisture-tolerant conditions (Scheme 31).^155^

**Scheme 30:**
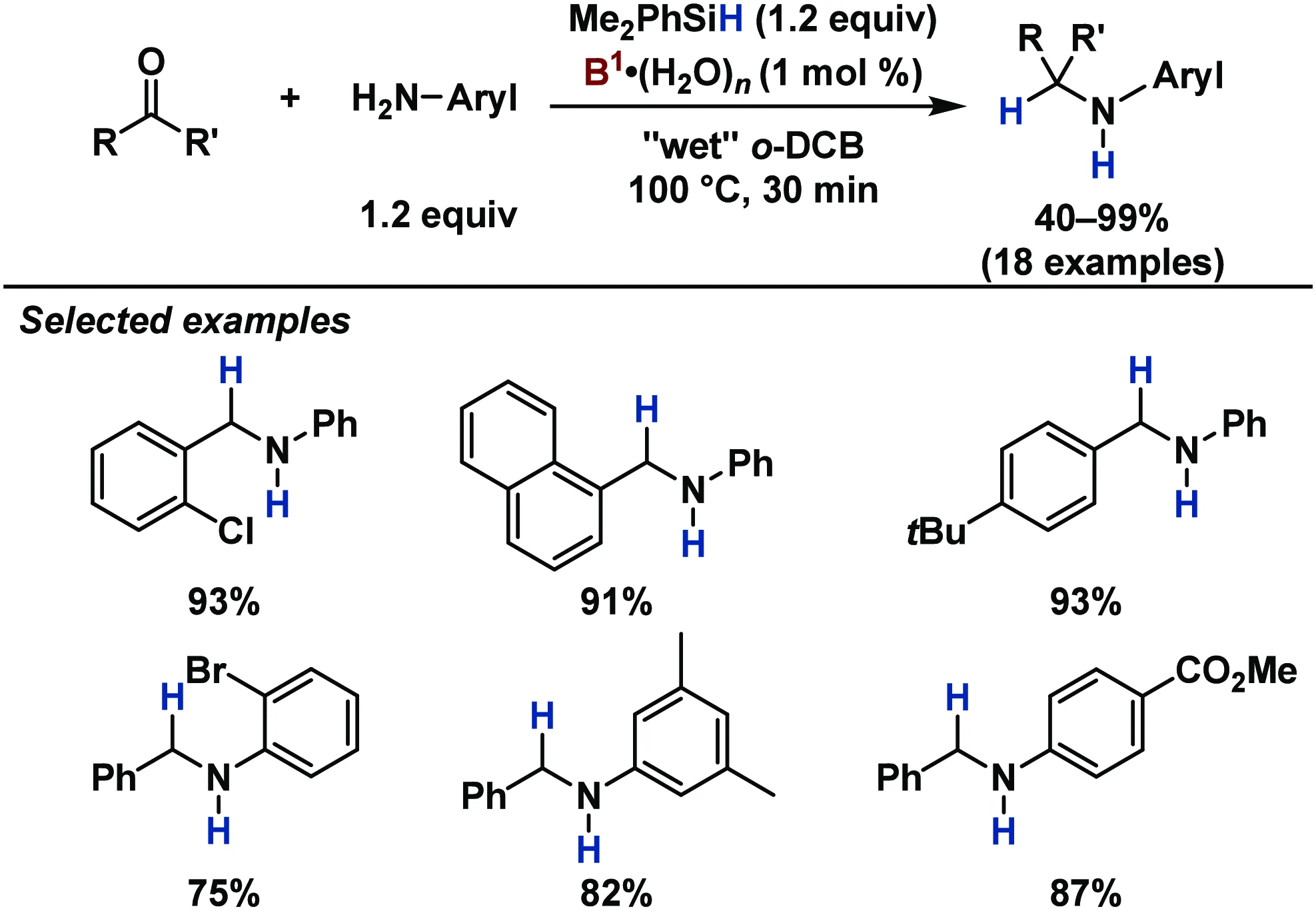
**B** ^**1**^-Catalyzed Reductive Amination with Me_2_PhSiH (*o*-DCB, *o*-Dichlorobenzene)

**Scheme 31:**
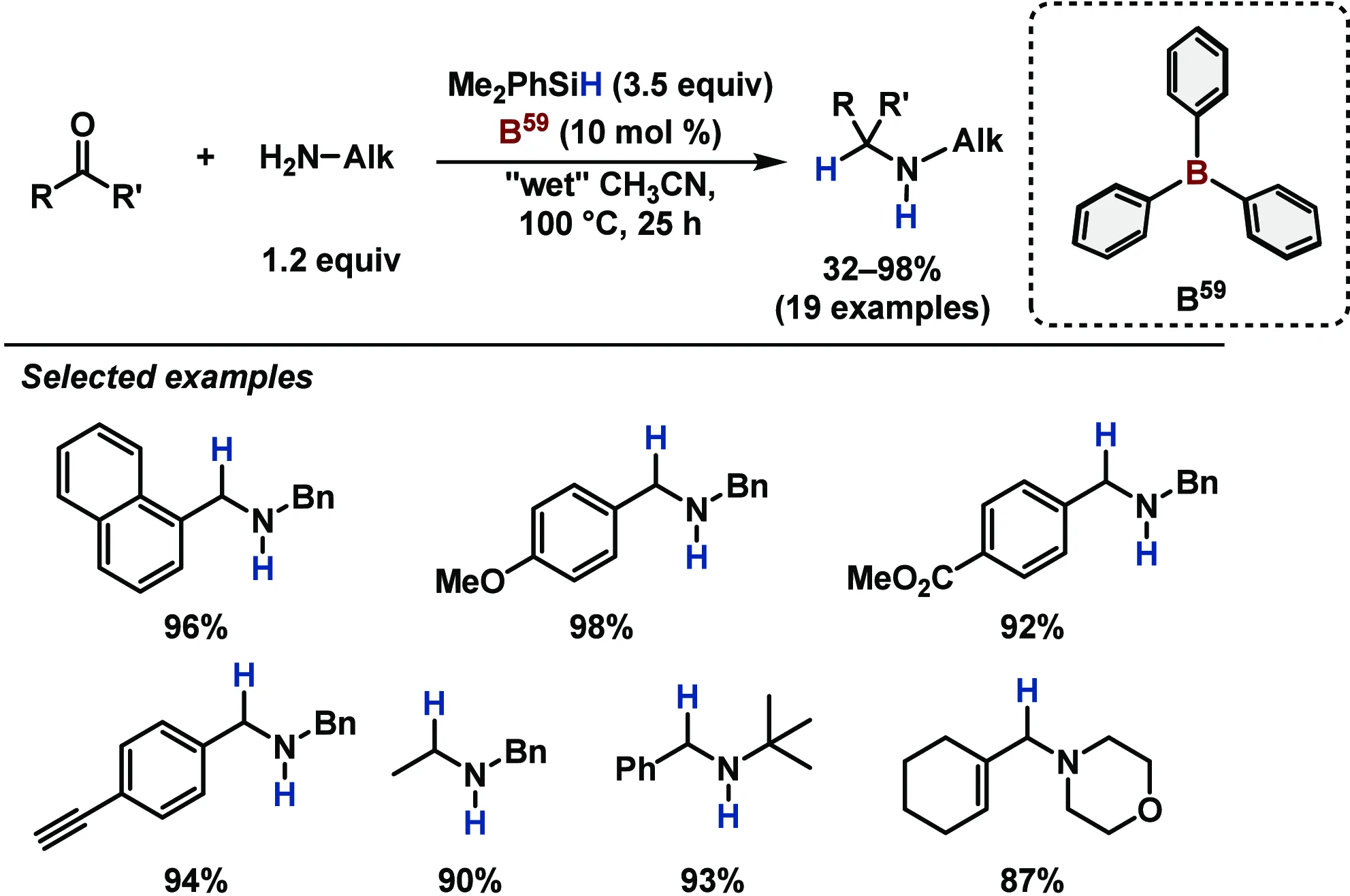
**B** ^**59**^-Catalyzed Reductive Amination with Me_2_PhSiH under “Wet” Conditions

Dearomatization chemistry represents another major advance. Cascade hydrosilylation–transfer hydrogenation of pyridines enables efficient conversion to partially or fully reduced products under mild conditions, overcoming limitations associated with catalyst deactivation and poor functional-group tolerance (Scheme 32).^156^ Related systems enable the reduction of quinolines, indoles, and quinoxalines, including enantioselective variants using chiral boranes.^157–160^

**Scheme 32:**
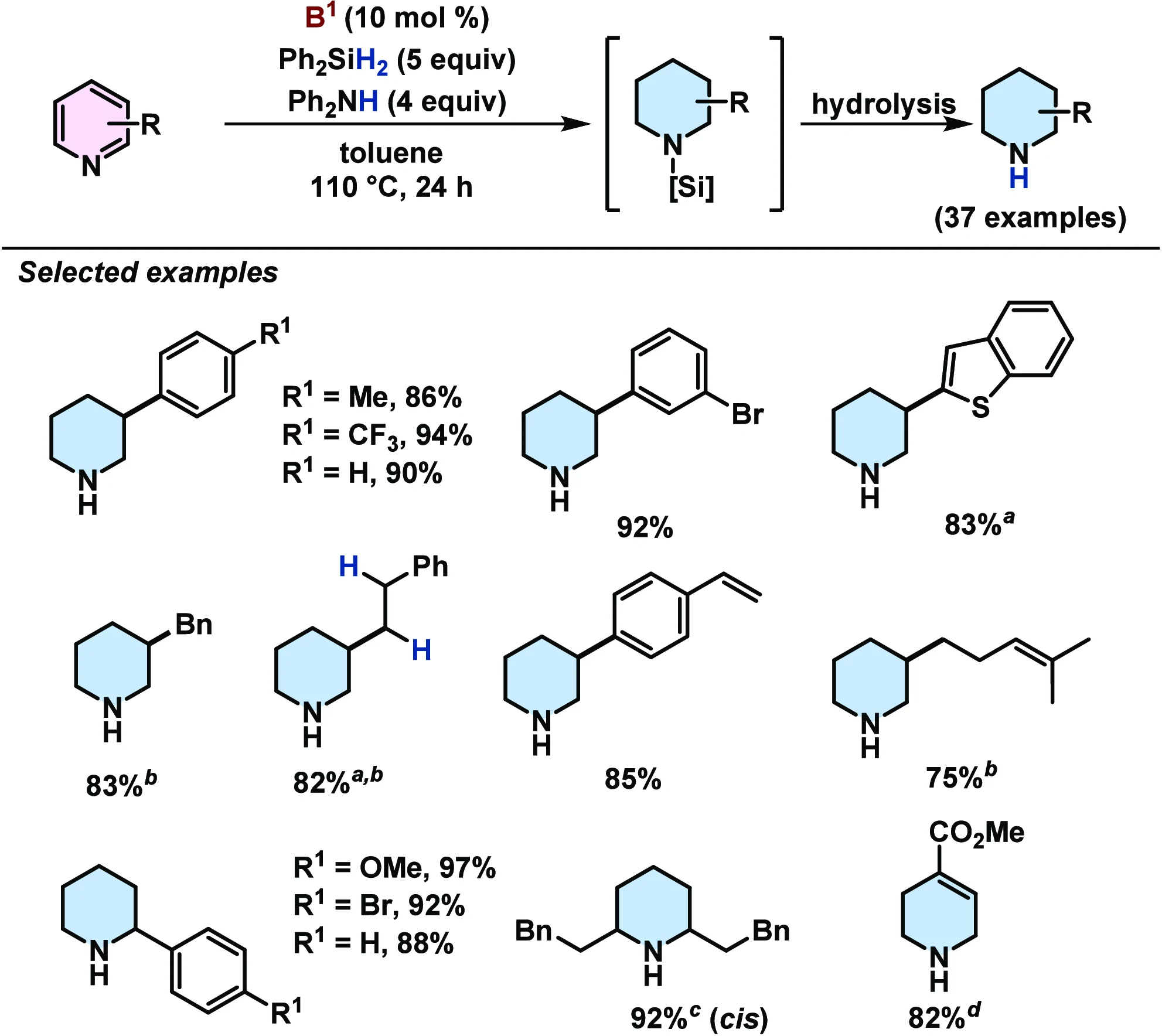
**B** ^**1**^-Catalyzed Cascade Reduction of Pyridines

a Ph_2_SiH_2_ (6 equiv).
b 120 °C.
c Ph_2_SiH_2_ (5 equiv) and PhNH_2_ (4 equiv).
d 5 mol % **B** ^**1**^.

Mechanistic studies have revealed important distinctions between borane and silylium catalysis. Both systems enable 1,4-selective hydrosilylation of N-heteroarenes via N-silylated intermediates, but differences in hydride donor strength can lead to divergent reactivity, including unusual C–C coupling pathways under silylium catalysis (Scheme 33).^161^ Beyond small-molecule reduction, borane–silane systems enable rapid intramolecular transformations. Use of bifunctional hydrosilanes such as (Me_2_HSiCH_2_)_2_ promotes efficient reduction–lactonization sequences, affording γ- and δ-lactones with high diastereoselectivity via intramolecular silicon–carbonyl interaction.^162^ A related trihydrosilane-based variant from the same group affords 1,4-azasilinanes through controllable Si–C bond formation.^163^

**Scheme 33:**
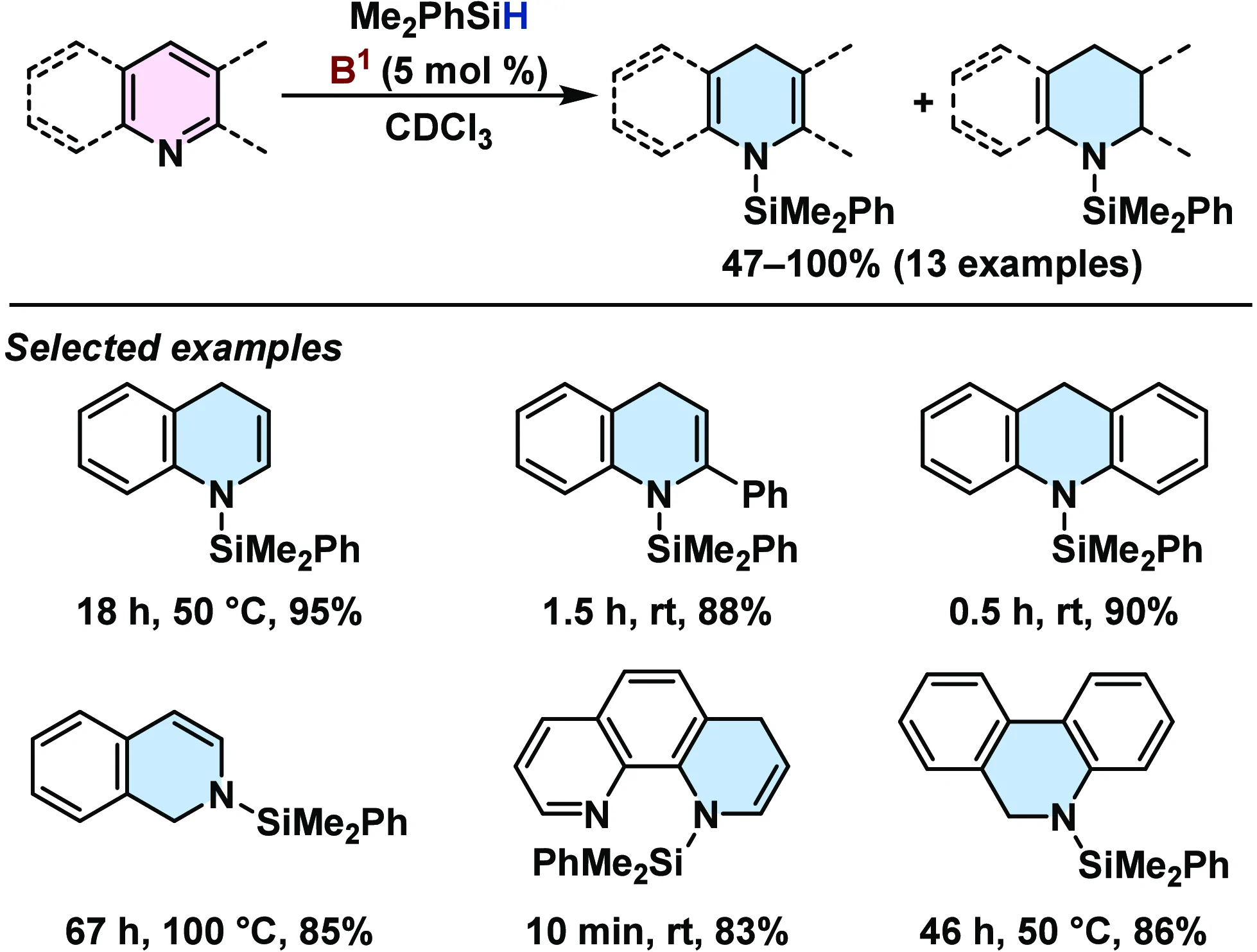
**B** ^**1**^-Catalyzed Monohydrosilylation of Pyridines with Me_2_PhSiH

Reduction of CO_2_ further highlights the versatility of the borane–silane systems. **B** ^**1**^-catalyzed hydrosilylation enables sequential reduction of CO_2_ to CH_4_, often accompanied by siloxane formation, while tandem Lewis acid systems improve efficiency by separating CO_2_ activation and downstream reduction steps.^164–166^ These processes illustrate how borane-mediated hydride transfer and silane oxophilicity can drive multistep deoxygenation beyond formate-level products.

Selective reduction of complex substrates has also been achieved. Chemoselective hydrosilylation of isatins enables controlled access to indolinones or indolines depending on reaction conditions.^167,168^ In addition, borane-catalyzed hydrosilylation of internal ynamides affords β-silyl enamides with excellent regio- and stereoselectivity,^169^ while furan substrates undergo cascade ring-opening (Scheme 34)^170^ and ring-closing processes to yield silicon-functionalized products.^171^

**Scheme 34:**
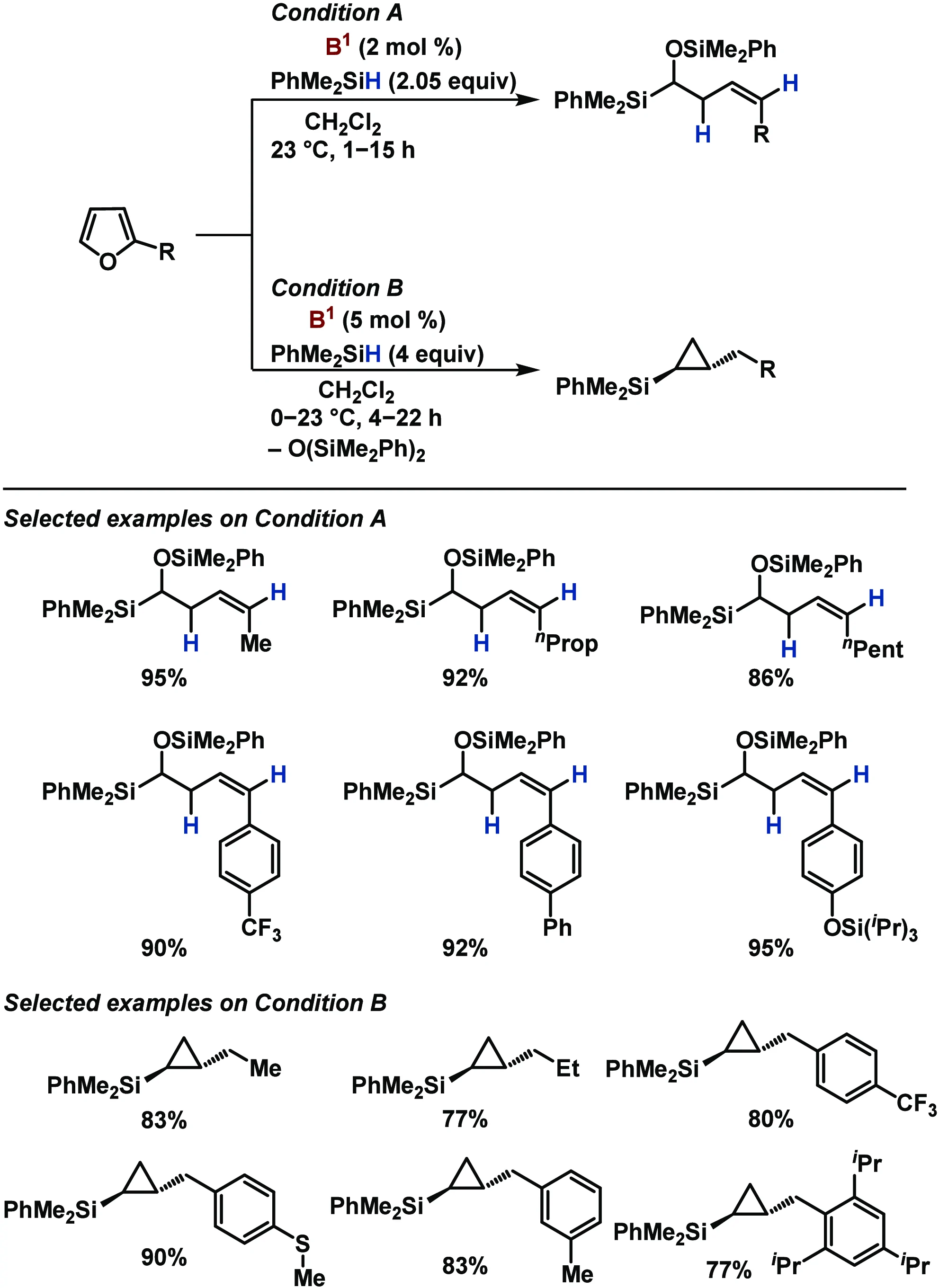
**B** ^**1**^-Catalyzed Cascade Silylative Transformation of Furans

BAr_3_ catalyst design again played a crucial role in advancing selectivity. Soós and co-workers developed proximal-back-strain-engineered boranes that regulate the reactivity of silylium–borohydride ion pairs, enabling selective reduction of esters to aldehyde acetals while suppressing overreduction (Scheme 35).^62^ These design principles have also enabled size-exclusion strategies that prevent catalyst deactivation and allow controlled hydrosilylation in complex environments.^172^

**Scheme 35:**
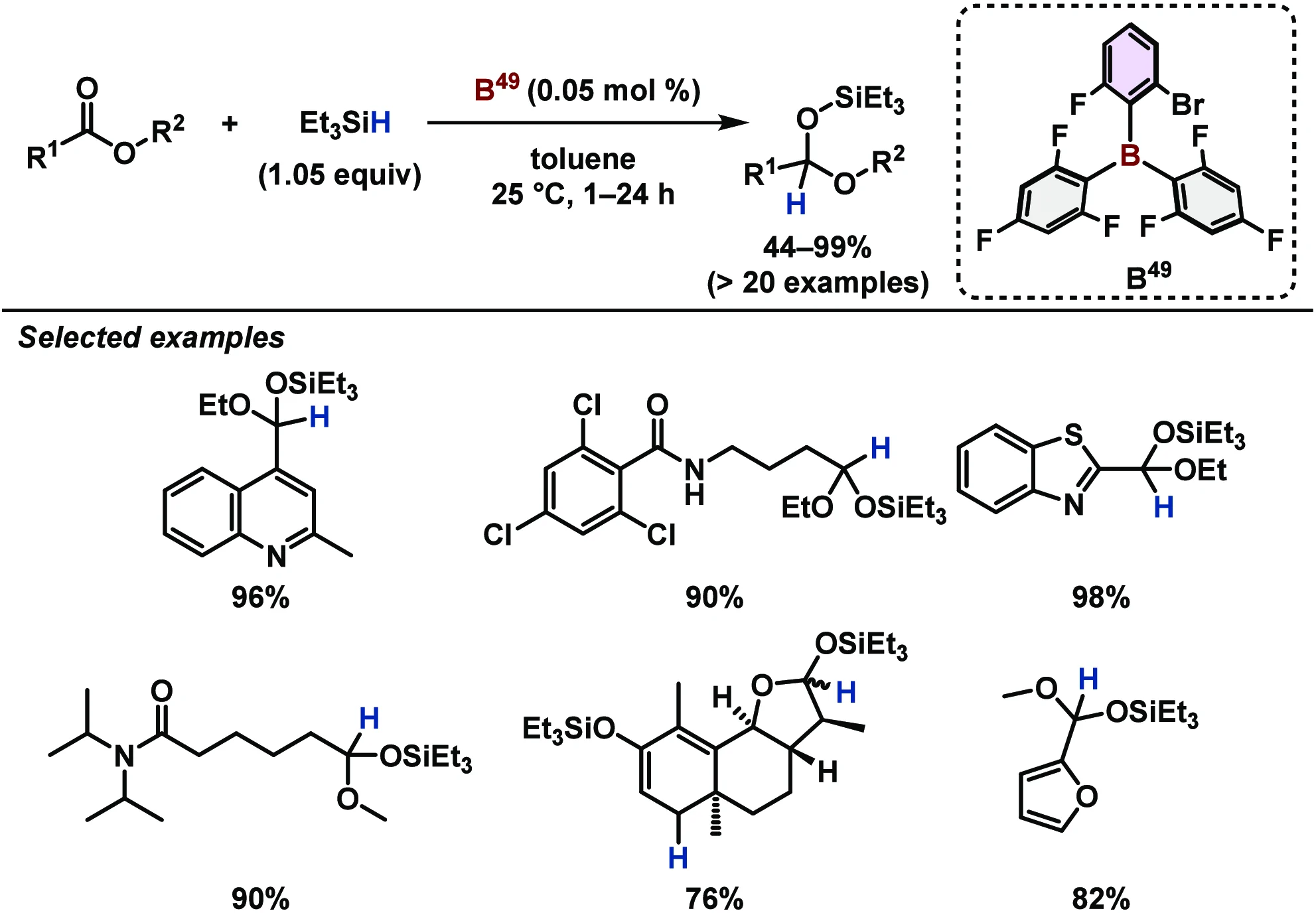
Selective Hydrosilylation of Esters to Acetals with **B** ^**49**^ and Et_3_SiH

Beyond reducing unsaturated bonds, borane–silane systems enable direct C–H functionalization. *Para*-selective C–H silylation of electron-rich arenes proceeds under neutral conditions without external hydrogen acceptors, demonstrating that Si–H activation can be harnessed for bond formation rather than reduction.^173^

Strained systems further illustrate the unique selectivity of borane catalysis. Hydrosilylation of methylenecyclopropanes and vinylcyclopropanes proceeded with ring retention, avoiding the ring-opening pathways commonly observed in transition-metal catalysis.^174,175^ These transformations were enabled by stabilization of cationic intermediates through β-silicon effects. Borane-catalyzed (4 + 1) cycloaddition of vinylcyclopropanes enabled formation of silolanes via sequential Si–H activation, silylium formation, and intramolecular cyclization.^176^ In parallel, finely tuned borane catalysts enabled selective hydrosilylation of esters to silyl acetals under mild conditions (Scheme 36),^177^ addressing the longstanding challenge of stopping reduction at the aldehyde stage while suppressing overreduction.

**Scheme 36:**
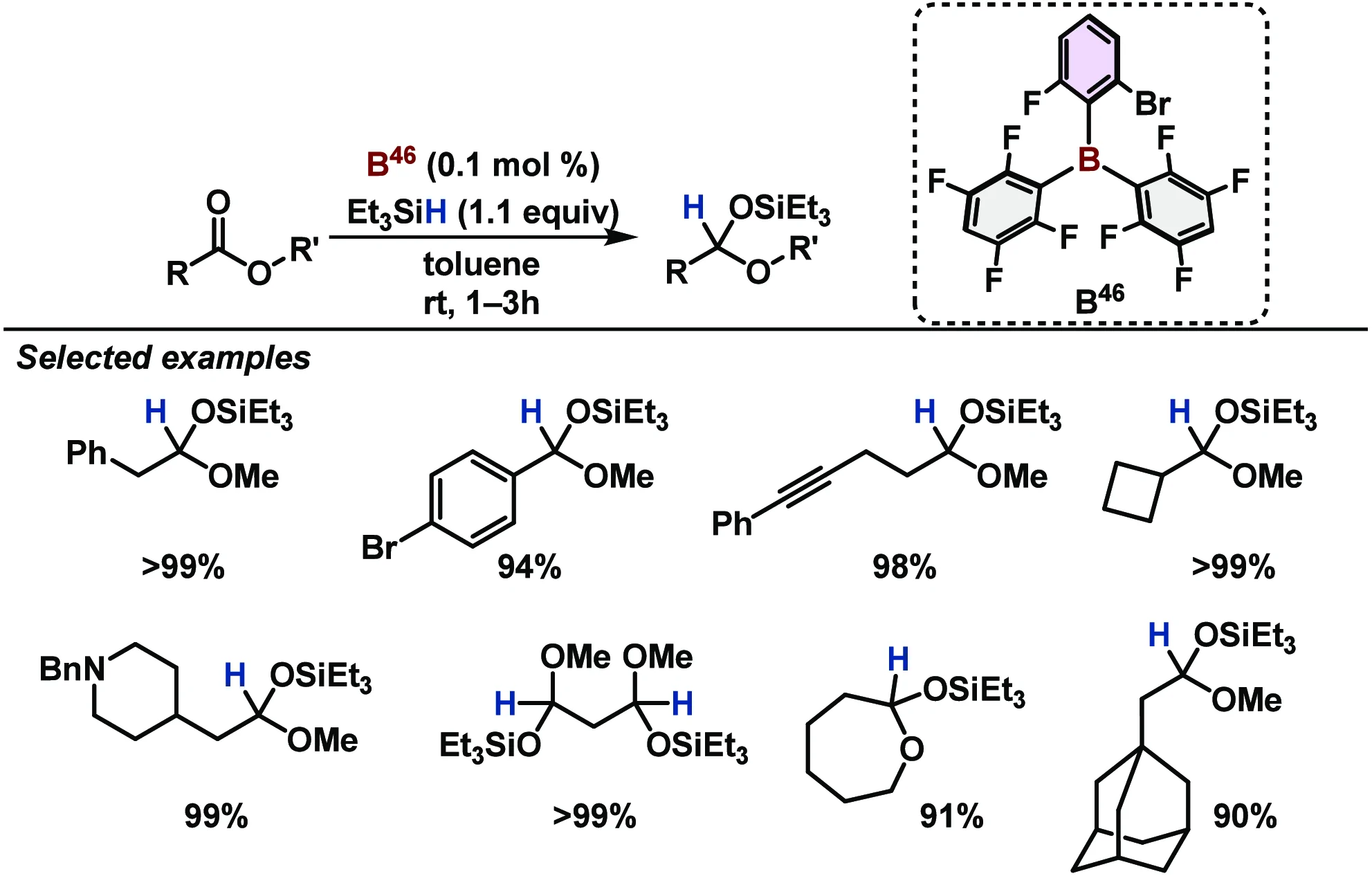
**B** ^**46**^-Catalyzed Hydrosilylation

### 4.2 Reductive Defunctionalization via C–Heteroatom Cleavage

Reductive defunctionalization via C–heteroatom bond cleavage using hydrosilanes and electron-deficient BAr_3_ has emerged as a powerful metal-free strategy, wherein BAr_3_ activate Si–H bonds to enable selective or exhaustive removal of oxygen- and heteroatom-based functionalities. Beyond classical carbonyl reduction, these systems now enable diverse C–O, C–N, and C–S bond cleavage processes across small molecules and polymers.

**B** ^**1**^-catalyzed defunctionalization of unsaturated polyols proceeded via cooperative activation of hydrosilanes, enabling selective C–O bond cleavage under mild conditions.^178^ With Et_3_SiH or Me_2_EtSiH, chemoselective deoxygenation of allylic and carbohydrate-derived substrates occurred via silyloxonium intermediates, favoring S_N_2′ pathways. Related strategies exploited *in situ* formation of borohydride from **B** ^**1**^/Et_3_SiH to achieve chemoselective C­(sp^3^)–OTs bond cleavage, providing practical access to deoxygenated diols and carbohydrate derivatives with broad functional-group tolerance (Scheme 37).^179^ At the macromolecular level, **B** ^**1**^/1,1,3,3-tetramethyldisiloxane systems enabled postpolymerization deoxygenation of poly­(methyl acrylate) to polypropylene while preserving backbone integrity.^180^ Near-quantitative conversion was achieved under mild conditions, and silane loading allowed controlled partial deoxygenation to afford functionalized materials with tunable residual groups.

**Scheme 37:**
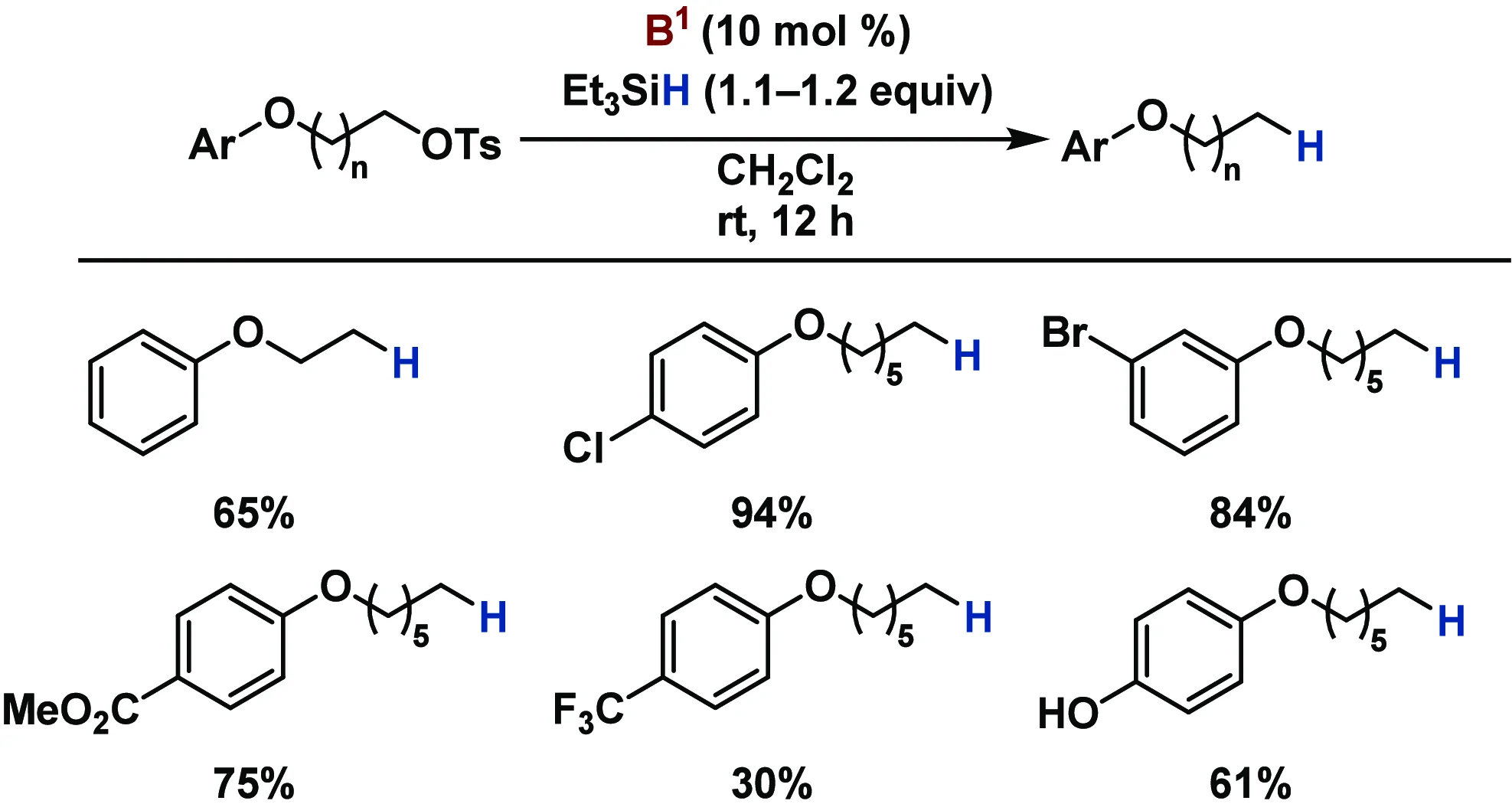
**B** ^**1**^-Catalyzed Chemoselective Cleavage of Tosylates in the Presence of Aryl Ethers

Directed, chemoselective C–N bond cleavage was also achieved. Hydroxy-directed **B** ^**1**^-catalyzed hydrosilylation enabled site-selective reduction of secondary amides using dihydrosilanes (PhMeSiH_2_ or PhSiH_3_), proceeding via dehydrogenative silylation to form tethered silyl ethers that promote intramolecular carbonyl activation through silylium-like intermediates.^181^ This strategy afforded hydroxy amines in high yields with excellent selectivity, even in multifunctional substrates. Complementarily, **B** ^**59**^-catalyzed systems employing PhMeSiH_2_ provided an alternative activation mode, enabling selective reduction of tertiary amides (Scheme 38)^182^ and CO_2_ under mild conditions through weaker, dynamic Lewis acid–substrate interactions.^183^

**Scheme 38:**
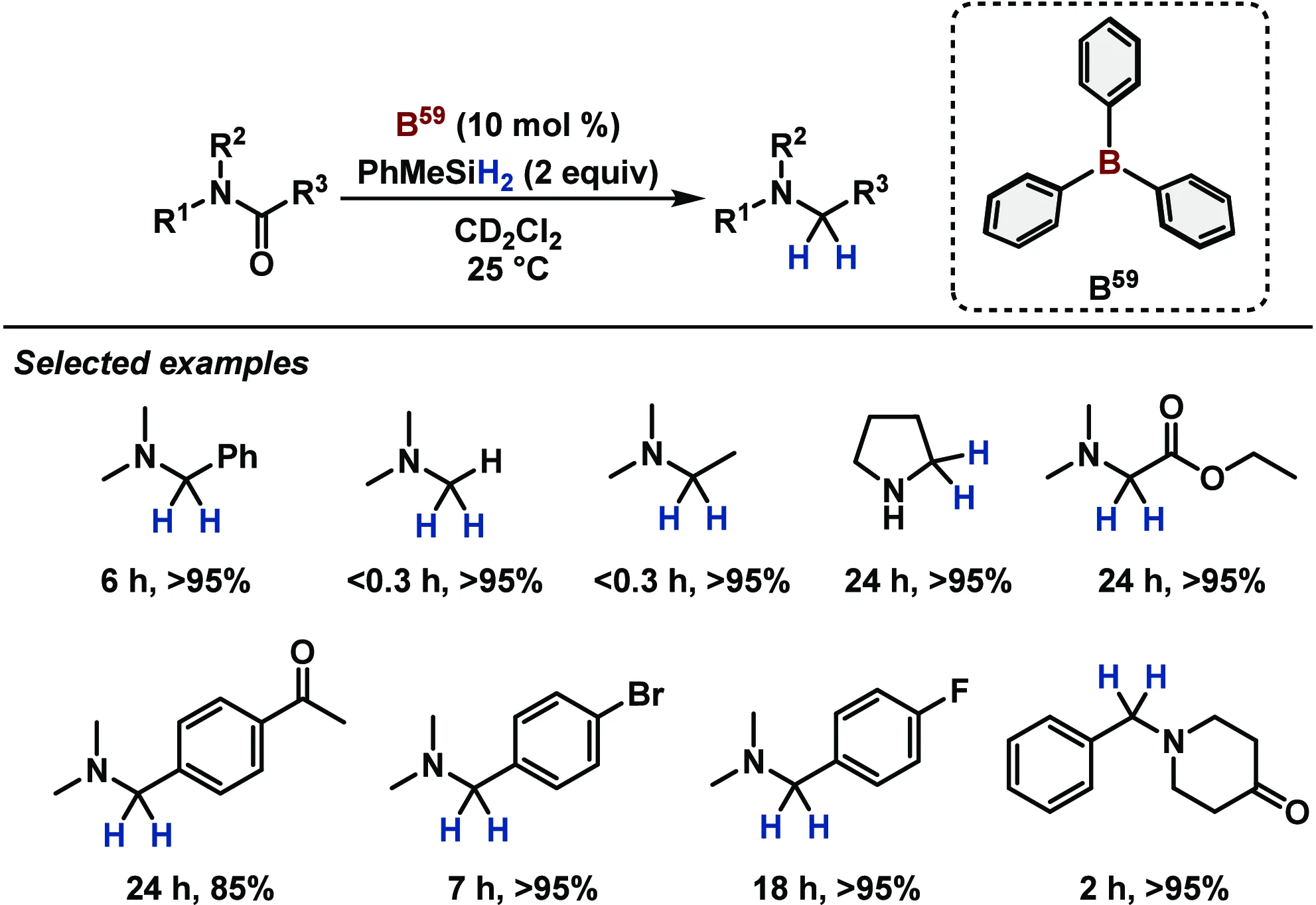
**B** ^**59**^-Catalyzed Reduction of Tertiary Amides Using PhMeSiH_2_

Reagent-controlled defunctionalization further highlighted the versatility of these systems. In **B** ^**1**^-catalyzed reduction of α-keto thioesters, silane identity dictated divergent pathways, with polymethylhydrosiloxane enabling exhaustive deoxygenation to thioethers, while other silanes afforded selective transformations.^184^ Complete C–N bond cleavage was likewise achieved using PhSiH_3_, enabling reductive denitrogenation of nitriles via sequential hydrosilylation and trisilylammonium intermediates, followed by S_N_1-type bond scission.^185^ This reactivity extended to regioselective ring opening of cyclic amines governed by carbocation stability (Scheme 39),^186^ as well as stepwise desulfurization of thioamides through controlled double hydrosilylation.^187^

**Scheme 39:**
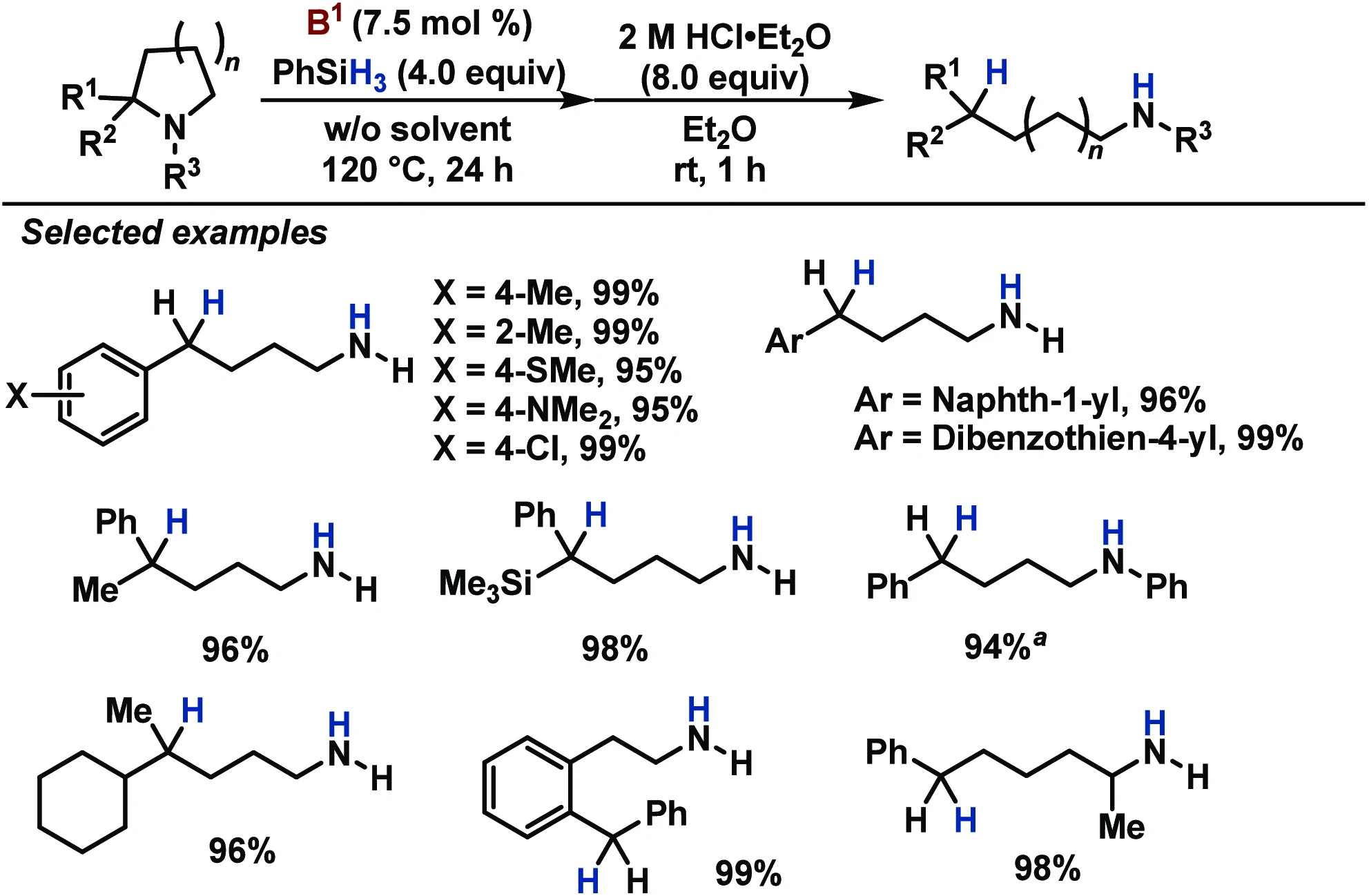
**B** ^**1**^-Catalyzed Ring Opening of Secondary and Tertiary Cyclic Amines

a PhSiH_3_ (3 equiv).

More complex transformations included tandem and unconventional pathways. **B** ^**1**^/PhSiH_3_ systems enabled efficient reduction of cyclic imides to pyrrolidines via stepwise hydride transfer (Scheme 40).^188^ Similarly, tandem cyclization–reduction platforms provided access to diverse *N* -heterocycles with product selectivity controlled by hydrosilane loading.^189–192^ Distinct mechanistic manifolds were observed in alkenyl–O bond cleavage, where borane-activated hydrosilylation followed by silicon-assisted β-elimination enabled selective removal of the vinyl groups (Scheme 41).^193,194^ Related systems employing specialized silanes facilitated selective C­(sp^3^)–O cleavage and full deoxygenation of carbonyl compounds while preserving sensitive functionalities.^195^

**Scheme 40:**
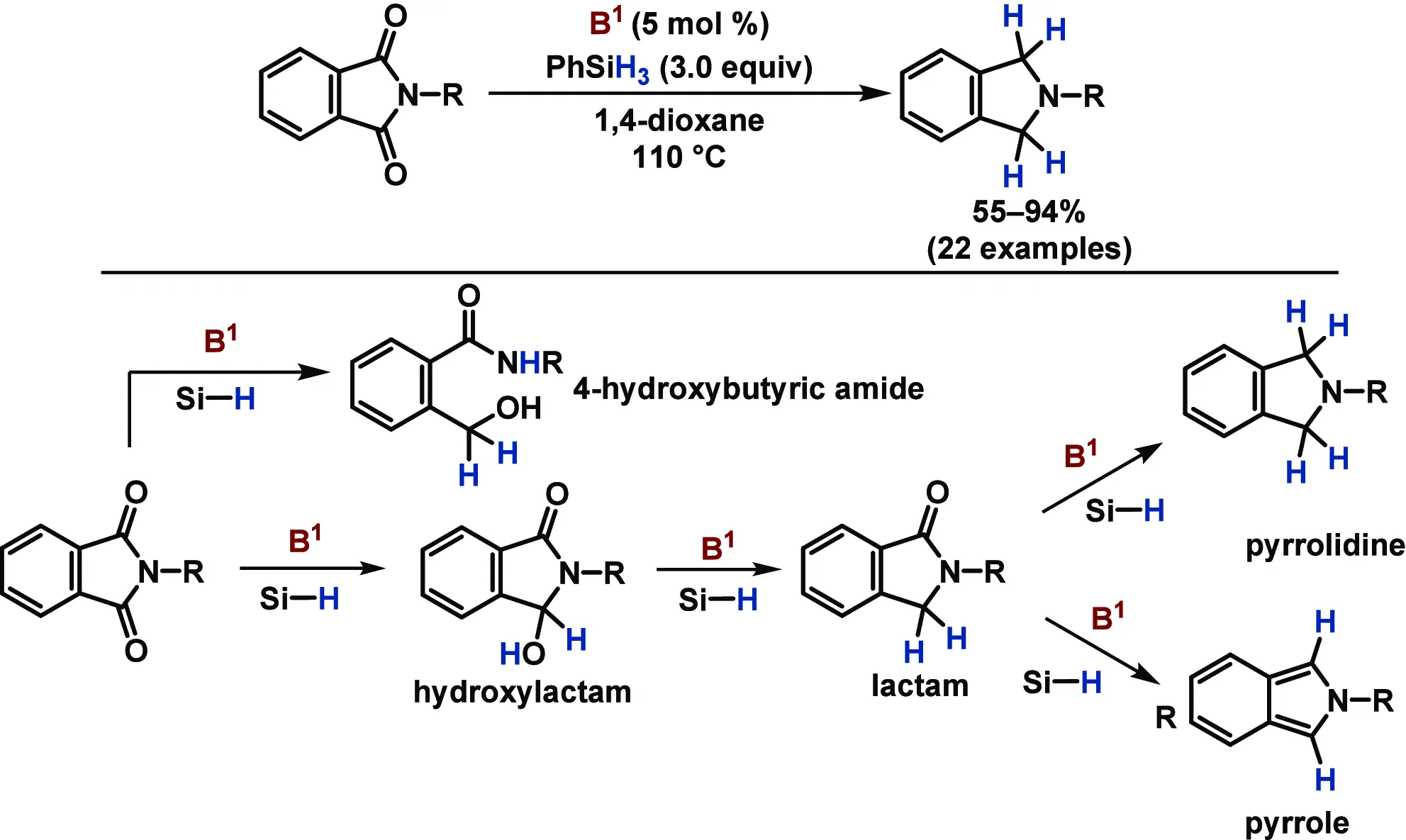
**B** ^**1**^-Catalyzed Reduction of Cyclic Imides with PhSiH_3_

**Scheme 41:**
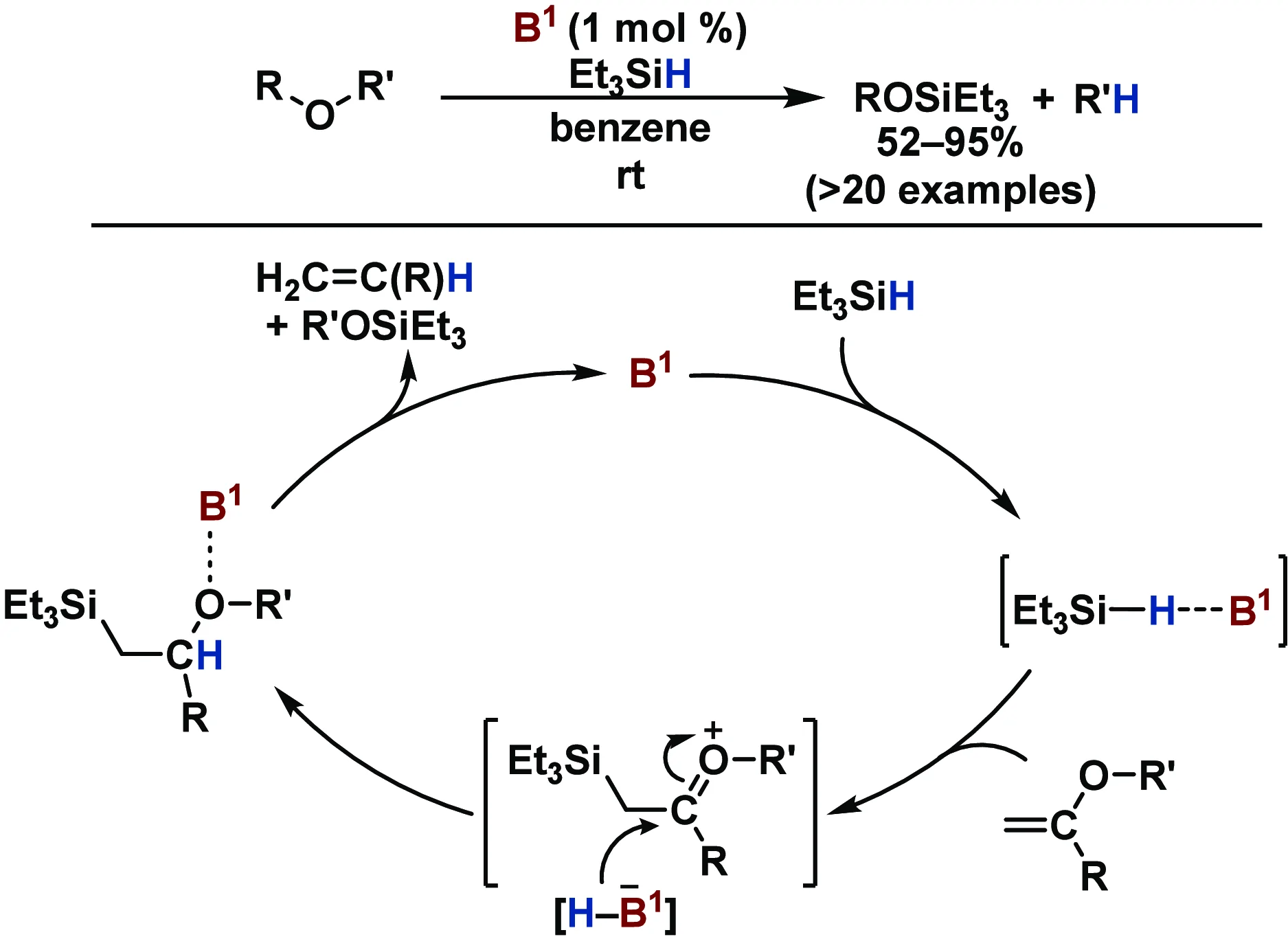
**B** ^**1**^-Catalyzed Reductive Cleavage of Enol Ethers with Et_3_SiH

Recent advances further demonstrated the breadth of borane-mediated defunctionalization. Bioinspired decarbonylation of aliphatic formates using **B** ^**1**^/Et_3_SiH enabled rapid conversion to alkanes via S_N_1-type pathways,^196,197^ while chemoselective 1,2-deoxygenation of alkynones proceeded under mild conditions with high selectivity.^196^ Cooperative systems, including Ir/**B** ^**1**^ catalysis, enabled full deoxygenation of tertiary amides with high efficiency and broad functional-group tolerance.^198^ Finally, borane-promoted dehydrogenative Si–N coupling enabled reductive deamination of amines without preactivation (Scheme 42),^199^ establishing nitrogen as a traceless functional handle in defunctionalization chemistry.^200,201^

**Scheme 42:**
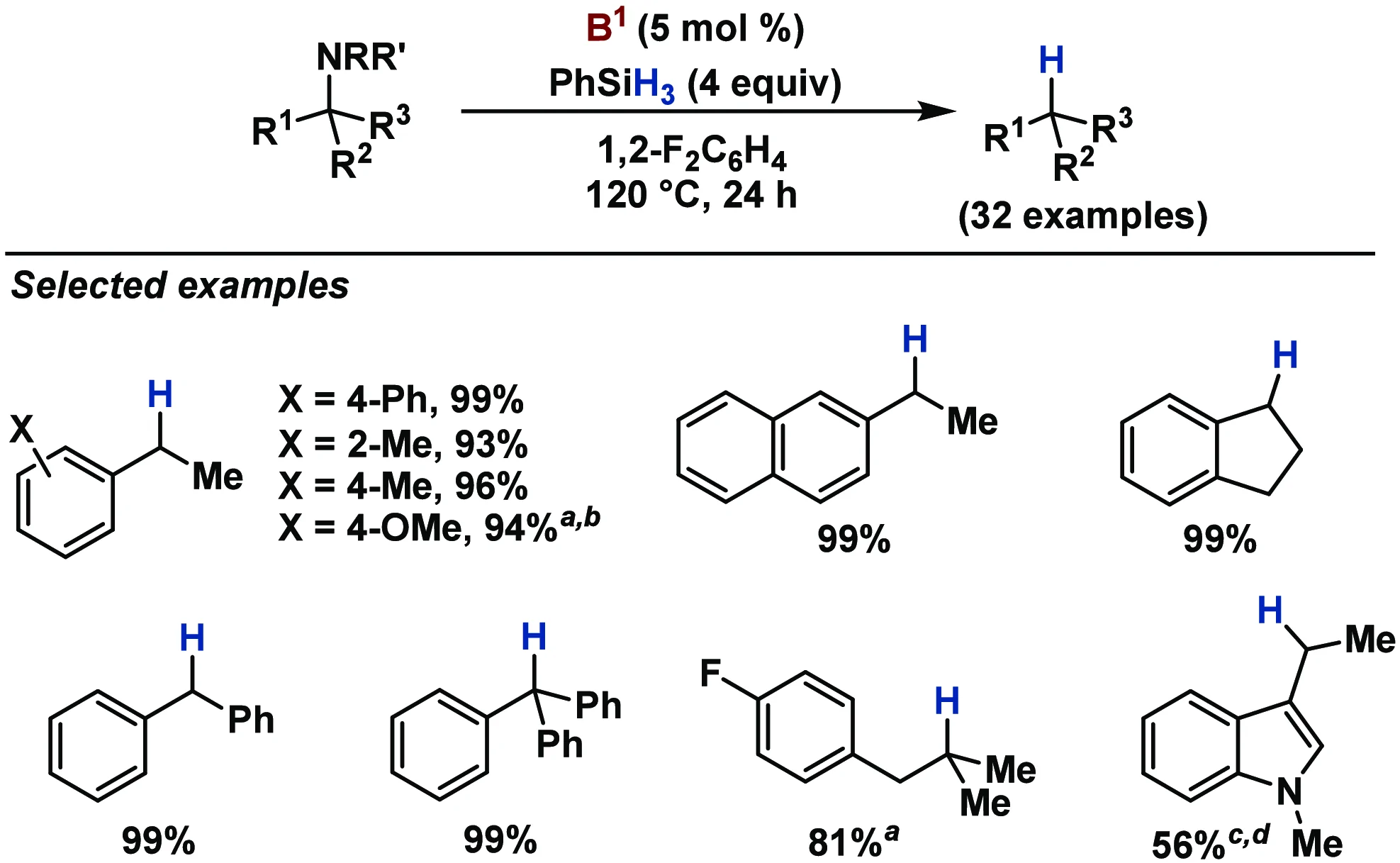
**B** ^**1**^-Catalyzed Reductive Deamination of 1°, 2°, and 3° Amines

a
**B** ^**1**^ (30 mol %).
b Me_2_PhSiH (4 equiv).
c PhSiH_3_ (2 equiv).
d 22% 1-methyl-3-ethylindoline was formed.

### 4.3 Other Transformations

Beyond conventional reduction and reductive defunctionalization, BAr_3_-catalyzed processes employing hydrosilanes have evolved to encompass a broader spectrum of transformations, including synthetically valuable bond-forming and rearrangement reactions.

In 2016, Gagné and co-workers reported a diastereoselective reductive carbocyclization strategy using the strong Lewis acid **B** ^**1**^ in combination with tertiary hydrosilanes (e.g., Et_3_SiH, Me_2_EtSiH) to transform unsaturated carbohydrates into densely functionalized cyclopropanes and cyclopentanes.^202^ The reaction proceeded across unsaturated 2-deoxysugars, allylic polyols, and dienes, delivering cyclopropanes with excellent diastereoselectivity (dr >98:2) and cyclopentanes bearing multiple contiguous stereocenters. Product selectivity was determined by substrate topology and silane identity, with preorganized intermediates favoring cyclopropanation.

Metal-free CO_2_ valorization was also achieved using **B** ^**1**^/hydrosilane systems, enabling the synthesis of benzimidazoles from *o*-phenylenediamines (Scheme 43).^203^ PhSiH_3_ served as the optimal reductant; bulkier silanes exhibited diminished reactivity, reflecting the importance of hydricity and steric accessibility. The transformation proceeded via an FLP pathway involving transient Lewis pair formation and CO_2_ capture to generate a carbamate-like intermediate.

**Scheme 43:**
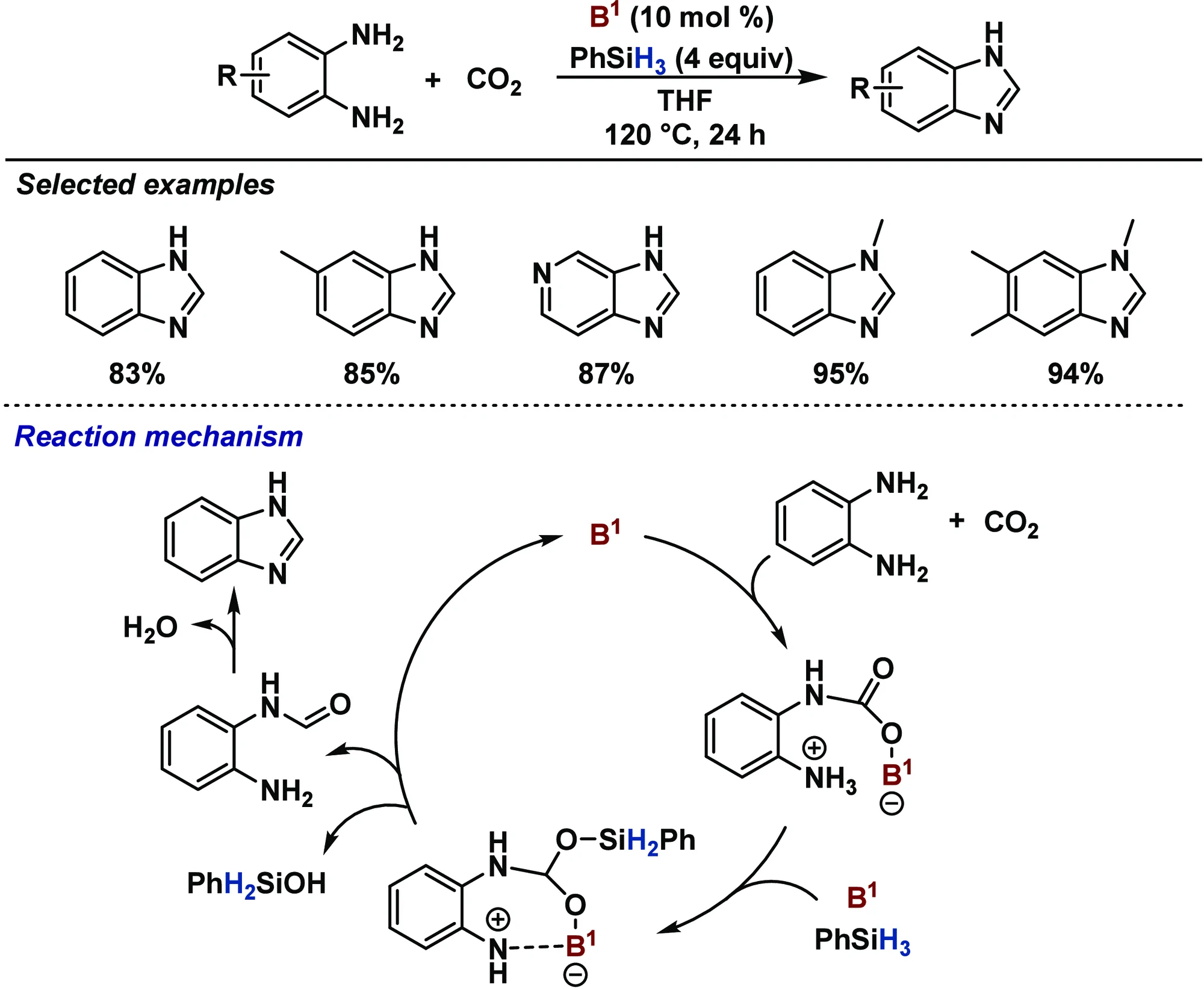
**B** ^**1**^-Catalyzed Cyclization of *o*-Phenylenediamines with CO_2_

A one-pot Meinwald rearrangement/reductive amination sequence converted epoxides into β-functionalized amines using **B** ^**1**^ and hydrosilanes (e.g., PhMe_2_SiH).^204^ This protocol integrated epoxide rearrangement, imine formation, and reduction within a single catalytic manifold, affording β-diarylamines in high yields (up to 99%), although aliphatic epoxides remained less reactive.

Reduction of heteroatom oxides has been an active area of research. Phosphine oxides were efficiently reduced to phosphines under metal-free conditions using **B** ^**1**^/PhSiH_3_ systems.^205^ Related hydrosilylation protocols enabled the conversion of sulfoxides and amine *N*-oxides to the corresponding sulfides and amines in good to excellent yields (Scheme 44).^206^ Exhaustive deoxygenation of sulfoxides and sulfones to sulfides was achieved under similar conditions.^207^

**Scheme 44:**
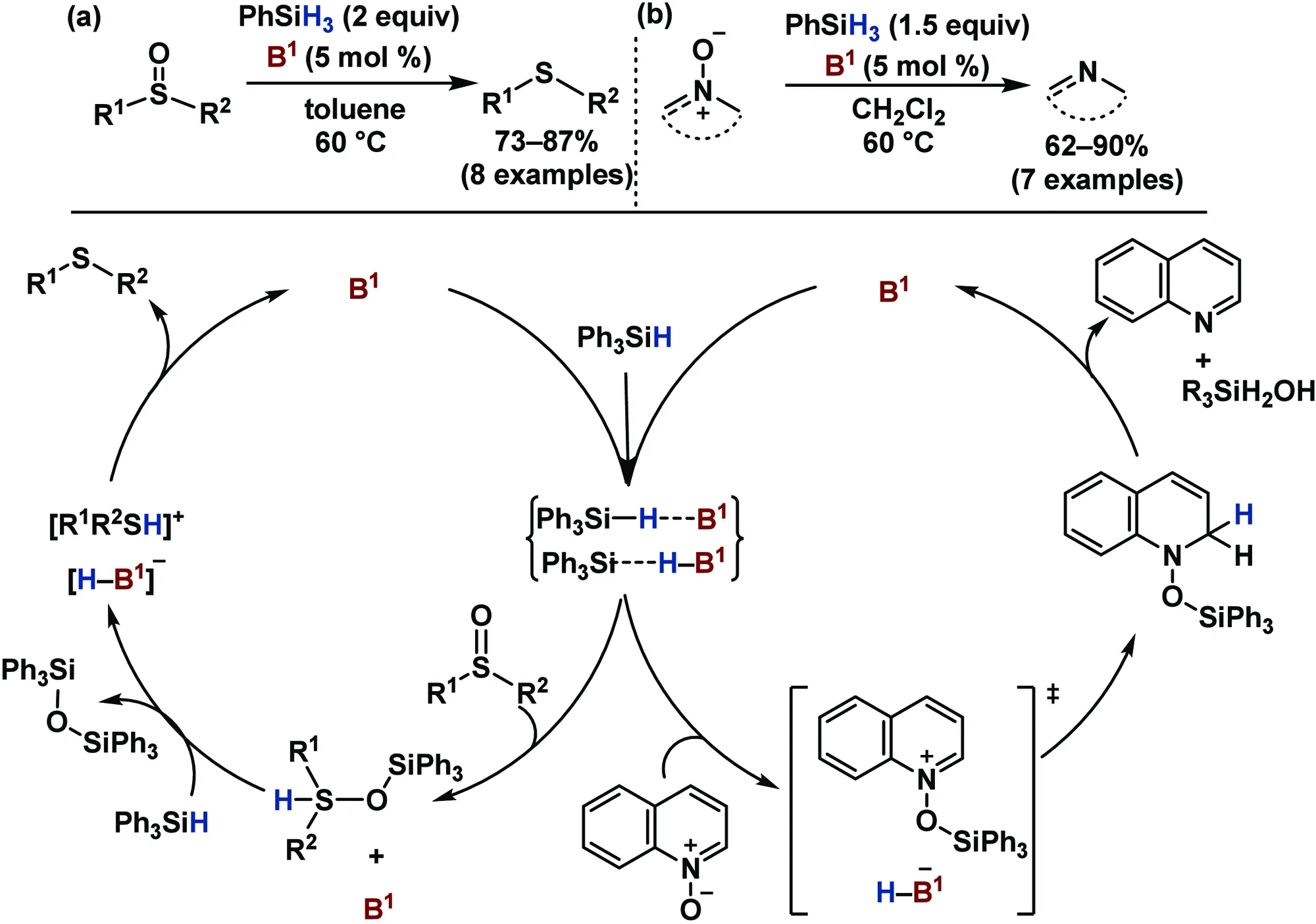
**B** ^**1**^-Catalyzed Reduction of Sulfoxides and *N*-Oxides with PhSiH_3_

Rearrangement chemistry represents an important direction. Reductive pinacol-type rearrangement of unactivated 1,2-diols was achieved using **B** ^**1**^/hydrosilane systems, overcoming the limitations of classical acid-mediated processes and providing rearranged alcohols with high stereocontrol.^208^ In a complementary approach, Oestreich and co-workers demonstrated reductive rearrangement of oximes and oxime ethers using hydrosilanes, highlighting the ability of borane catalysts to mediate complex skeletal reorganization.^209^

C–C bond transformations further illustrated the expanding scope of borane catalysis. Reductive cleavage of bis­(indolyl)­methanes enabled access to C3-alkylated indoles via silylium–borohydride intermediates (Scheme 45).^210^ Complementarily, reductive C–C coupling of indoles with carboxylic acids provided direct access to bis­(indolyl)­methanes under mild conditions, replacing traditional aldehyde electrophiles and tolerating diverse functional groups.^211^

**Scheme 45:**
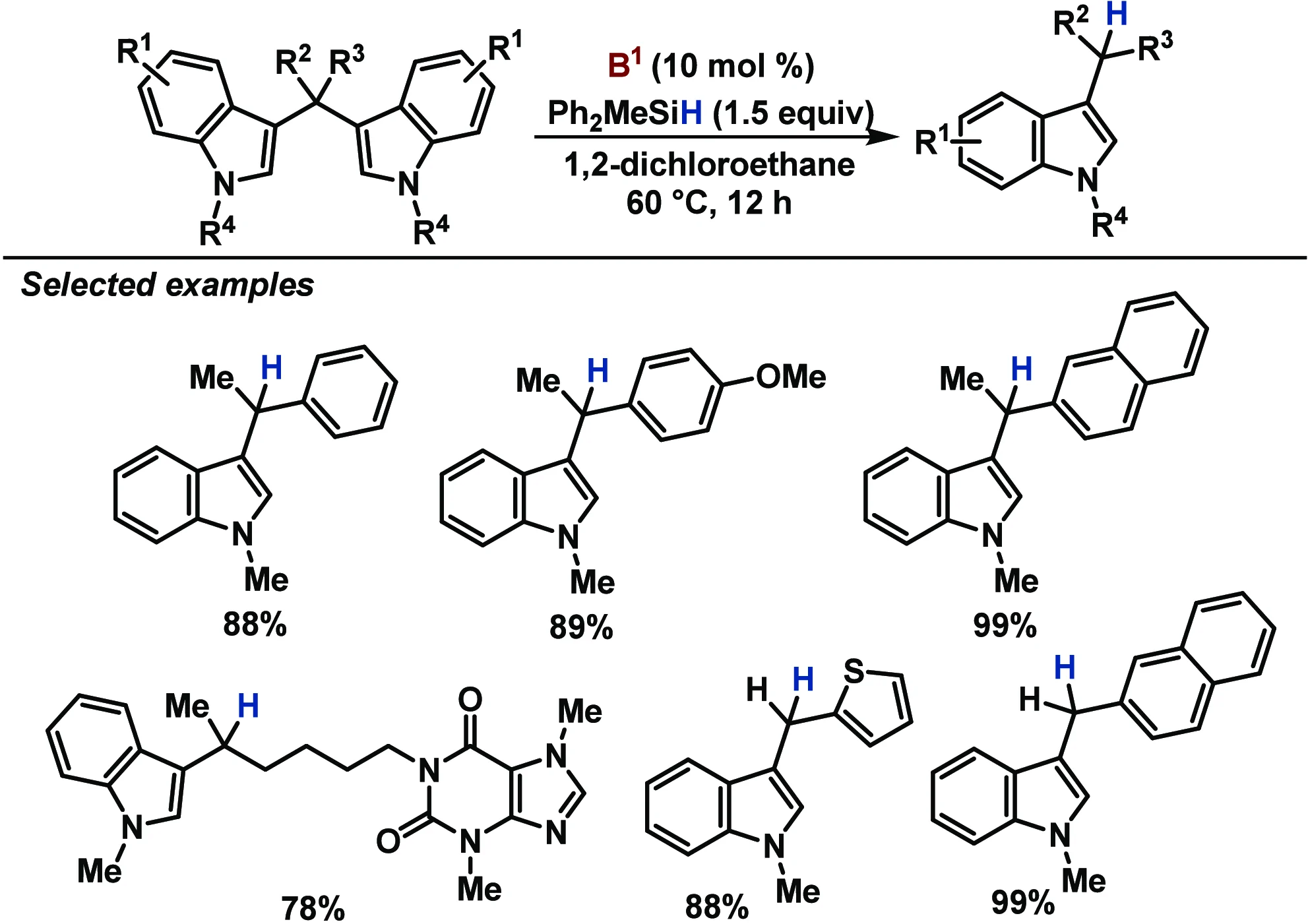
**B** ^**1**^-Catalyzed Reductive C–C Bond Cleavage of Bis­(indolyl)­methanes

Additional bond-forming processes included electrophilic cyclization–silylation of alkynes, enabling access to 3-silyl heterocycles such as benzothiophenes, benzofurans, and indoles.^212^ Unusual reactivity was observed in reductive ring opening of oxetanes, where FLP-type systems promoted double hydrosilylation coupled with aryl migration to afford rearranged hydrocarbons in excellent yields.^213^

Recent advances highlighted the potential of borane catalysis for skeletal editing and complex molecular transformations. Oestreich and co-workers reported a **B** ^**1**^/MePhSiH_2_ system enabling skeletal rearrangement of amines via *N*-hydroxylamine intermediates (Scheme 46).^214^ Selective ring-opening reduction of cyclic anhydrides was also achieved, affording unsymmetrical silyl ether–silyl ester products under mild conditions with broad substrate scope.^215^ Notably, Sarpong and co-workers introduced a conceptually distinct reductive amination of C–C σ bonds, enabling formal nitrogen insertion and carbon extrusion in azacycles, with substrate-controlled divergence between ring-contraction and aryl-migration pathways.^216^

**Scheme 46:**
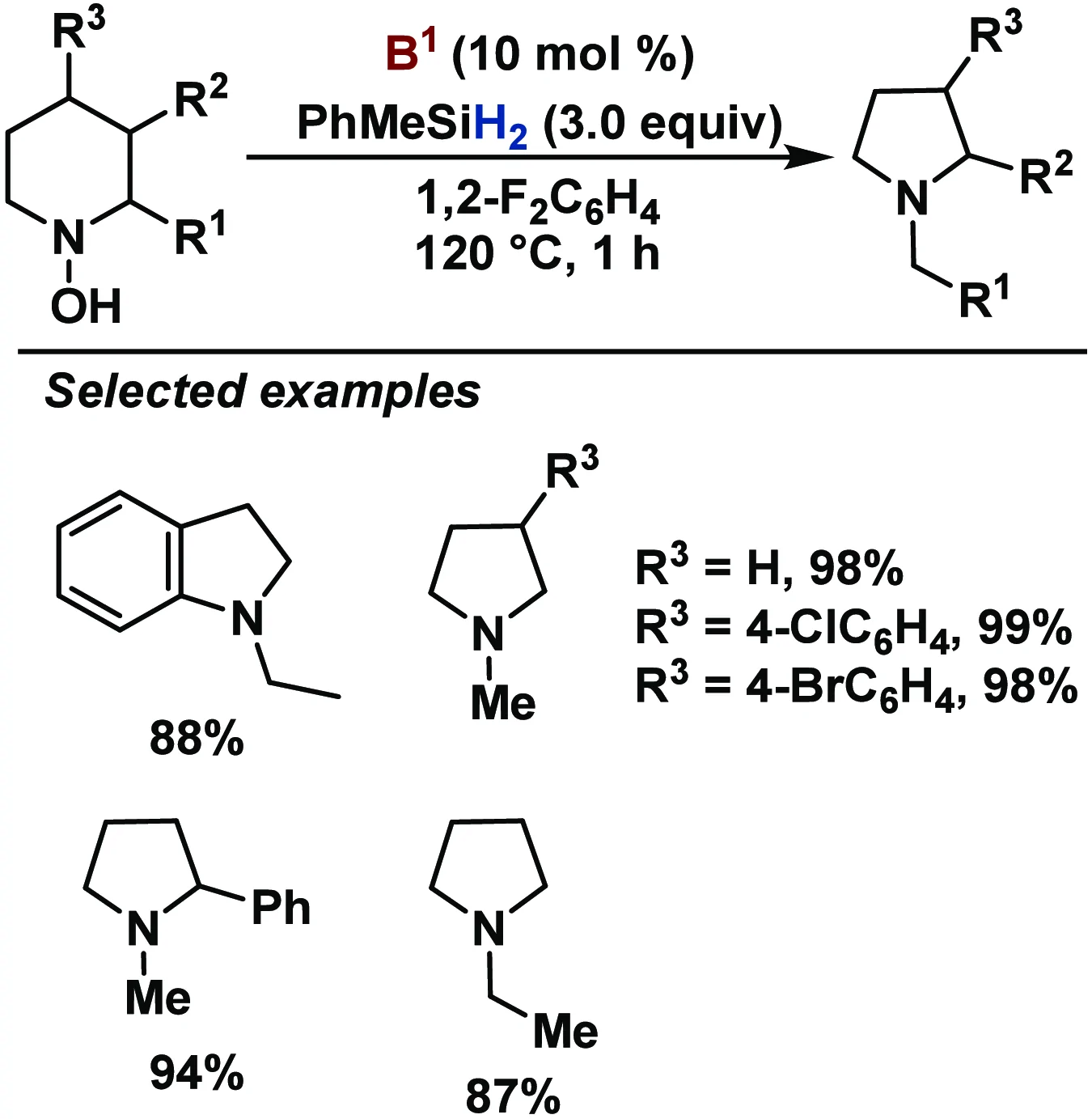
Ring Contraction of Saturated Cyclic Amines and Rearrangement of Acyclic Amines

Finally, Song and co-workers reported a **B** ^**1**^-catalyzed (5 + 1)-annulation of enynamides with hydrosilanes (e.g., PhSiH_3_, BnSiH_3_), enabling direct synthesis of 1,4-azasilines (Scheme 47).^217^ The transformation proceeded via generation of a silylium-like intermediate, followed by β-hydrosilylation and intramolecular Si–C bond formation, affording azasilines and benzo-fused analogues in moderate to excellent yields.

**Scheme 47:**
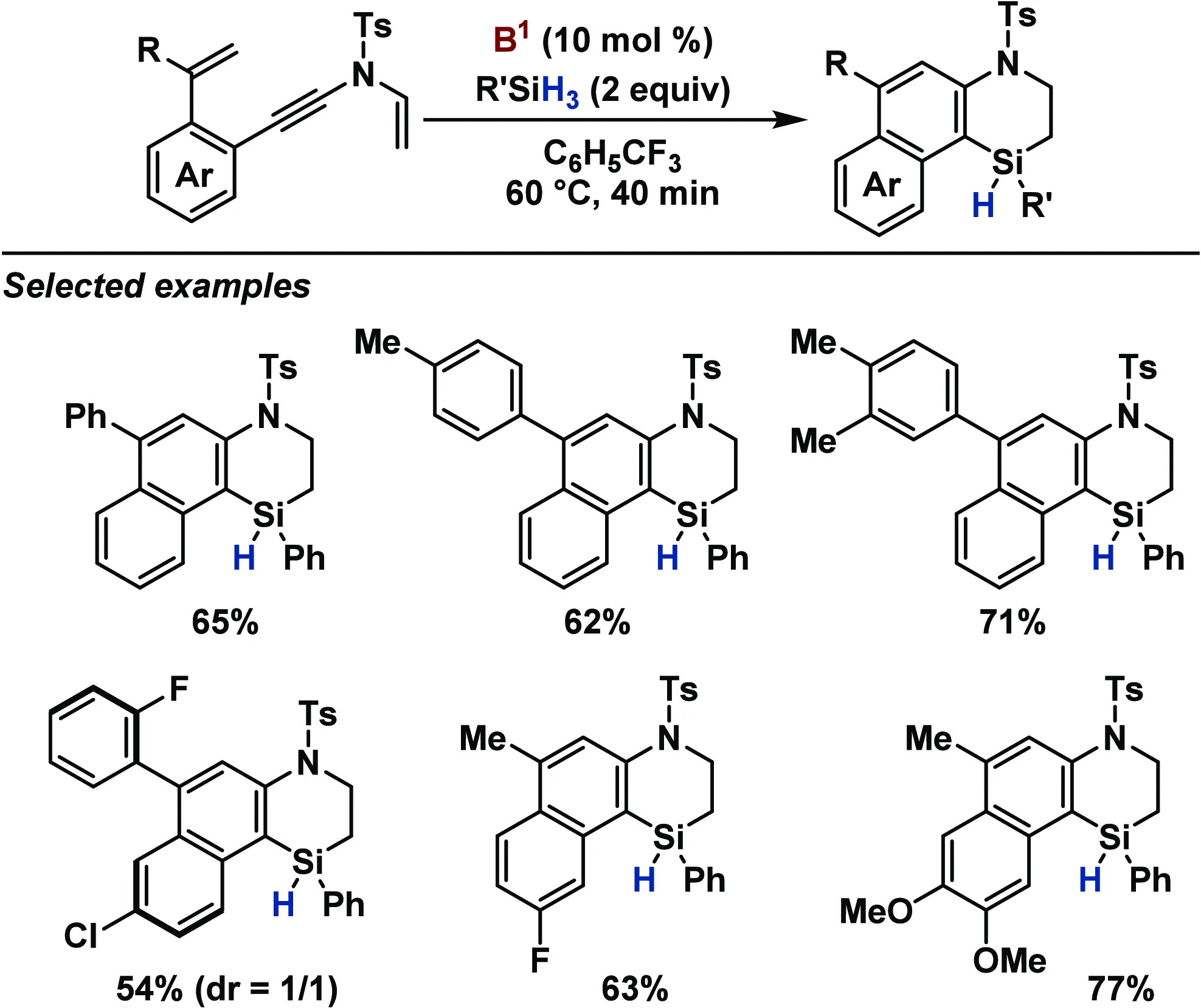
**B** ^**1**^-Catalyzed (5 + 1)-Annulation of Enynamides with Trihydrosilanes

## 5 Triarylborane-Catalyzed Transformations Using Hantzsch Esters

The Hantzsch ester, first reported by Arthur Hantzsch in 1881, is a prototypical organic hydride donor widely used as a biomimetic analogue of NAD­(P)­H.^218–222^ It delivers two hydrogen equivalents to unsaturated substrates through aromatization to a pyridine derivative, enabling reduction of C=C, C=N, and C=O functionalities. While extensively employed in Brønsted acid- and transition-metal-catalyzed transfer hydrogenation, its integration into borane-mediated systems has only recently emerged.

Early mechanistic studies established that Hantzsch esters can transfer hydride directly to strong Lewis acids such as **B** ^**1**^, forming pyridinium–borohydride ion pairs.^223^ Building on these insights, borane-catalyzed transfer hydrogenation has been developed as an efficient metal-free platform for reductive transformations.

A key advance was the use of strongly Lewis acidic boranes to activate carbonyl substrates. Uozumi and co-workers demonstrated that **B** ^**24**^ efficiently catalyzed the reduction of aldehydes using Hantzsch esters under mild conditions, affording primary alcohols in high to quantitative yields with broad functional group tolerance, including halides, nitro, cyano, esters, and heteroarenes (Scheme 48).^224^ Notably, the reaction proceeded efficiently at room temperature for aromatic substrates and at elevated temperatures for aliphatic systems, highlighting the versatility of this approach.

**Scheme 48:**
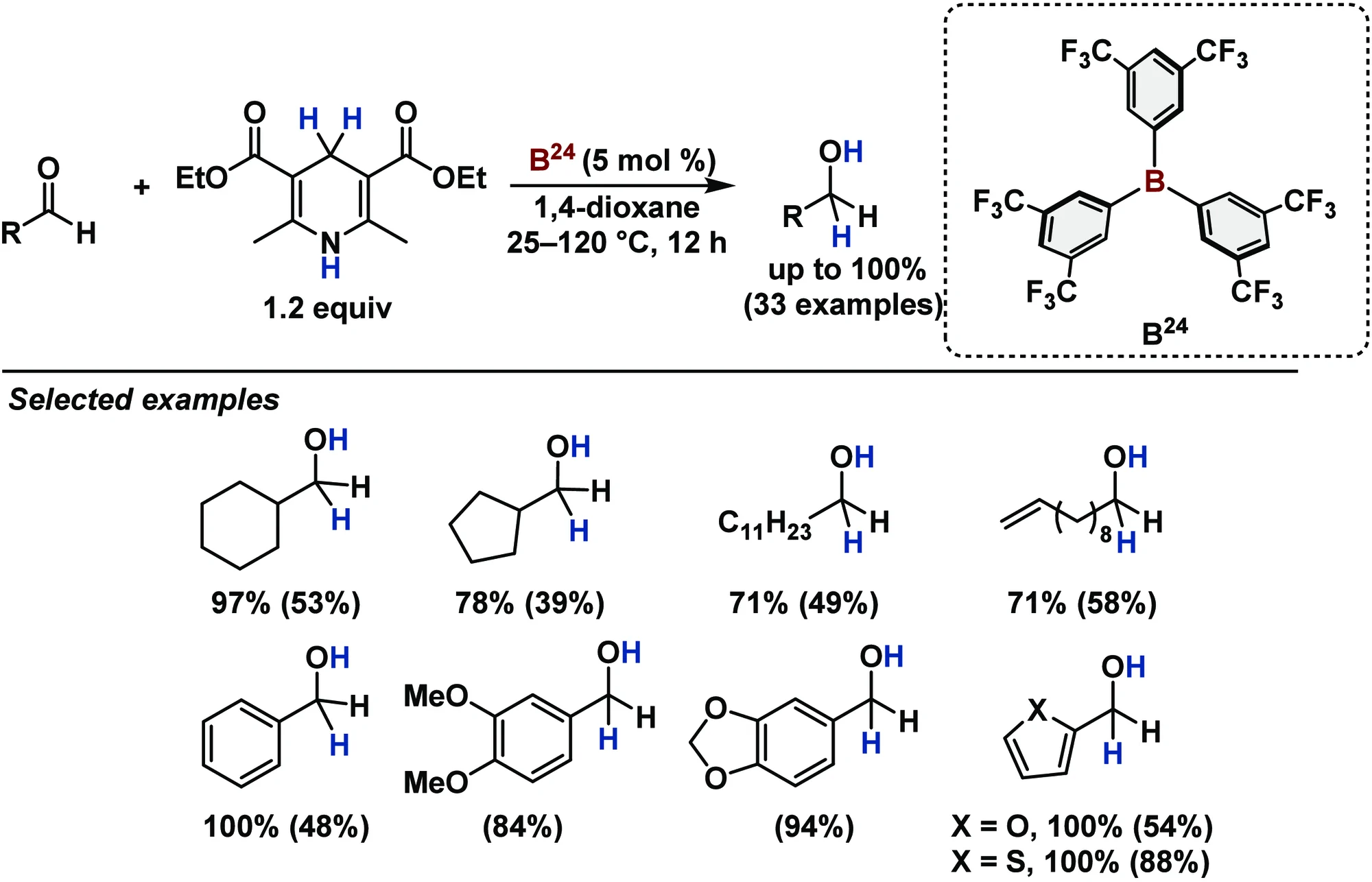
**B** ^**24**^-Catalyzed Hydrogenations of Aldehydes with a Hantzsch Ester^a^

a Isolated yields are given in parentheses.

Beyond carbonyl reduction, borane/Hantzsch ester systems enabled hydroaromatization and C–C bond-forming transformations. Reduction of *p*-quinone methides and related fuchsones provided access to structurally diverse diaryl- and triarylmethanes under mild conditions, with broad tolerance toward functional groups such as halides, nitro groups, esters, and heterocycles.^225^ These transformations proceeded efficiently for electron-rich substrates, while electron-deficient systems exhibited slightly diminished reactivity (Scheme 49).

**Scheme 49:**
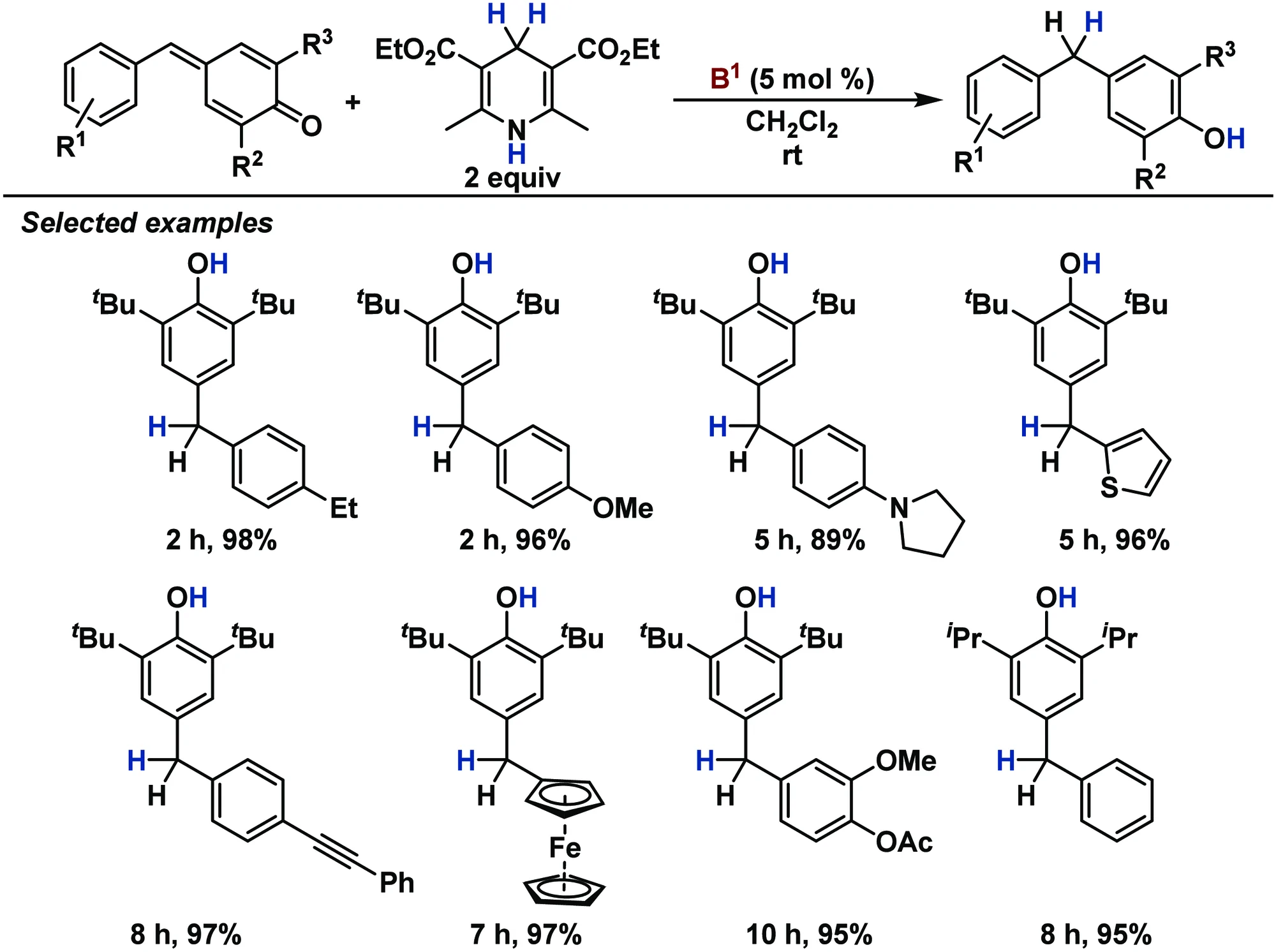
**B** ^**1**^-Catalyzed Reduction of *p*-Quinone Methides and Fuchsones to Access Unsymmetrical Diaryl- and Triarylmethanes

Transfer hydrogenation of imines has also been achieved with a high efficiency. Using **B** ^**1**^ in combination with Hantzsch esters, a broad range of imines were reduced to amines in excellent yields under very low catalyst loadings.^226^ The reaction is proposed to proceed via the formation of an iminium borohydride intermediate generated from interaction between the borane-activated Hantzsch ester and the imine. Extension to asymmetric variants using chiral borane systems enabled the enantioselective reduction of ketimines, although enantioselectivity remained moderate (Scheme 50).

**Scheme 50:**
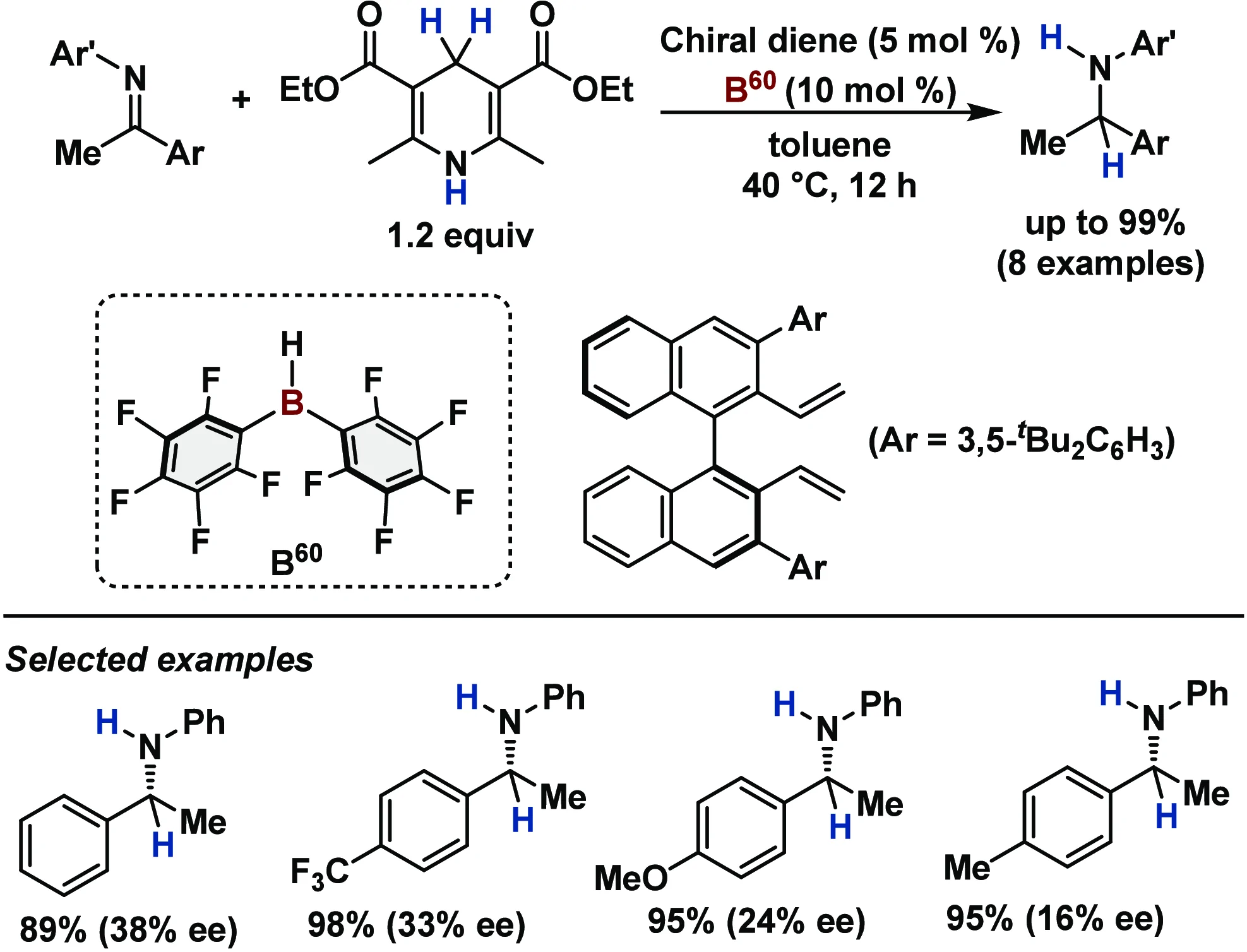
**B** ^**60**^-Catalyzed Hydrogenations of Imines with a Hantzsch Ester

Mechanistic studies provided important insight into catalyst-dependent reactivity. Detailed spectroscopic and computational investigations revealed that borane-catalyzed transfer hydrogenation of aldehydes proceeds via Lewis acid activation of the carbonyl, followed by direct hydride transfer from the Hantzsch ester, rather than through a discrete borohydride pathway.^227^ The superior performance of **B** ^**24**^ arose from its optimal balance between strong substrate activation and minimized product inhibition, favoring formation of reactive aldehyde–borane complexes while suppressing competitive binding to alcohol products or the Hantzsch ester. In contrast, other boranes exhibited either excessive binding to nonproductive species or higher activation barriers, thereby reducing catalytic efficiency.

## 6 Triarylborane-Catalyzed Transformations Using Ammonia Borane

Ammonia borane (H_3_N·BH_3_) is regarded as an attractive hydrogen source because of its high gravimetric hydrogen content (19.6 wt %), low molecular weight, ready availability, and operationally convenient handling. The polarized B–H and N–H bonds have enabled H_3_N·BH_3_ to function as both a hydride and a proton donor under relatively mild conditions, making it a highly effective reductant in borane-catalyzed transfer hydrogenation reactions of a wide range of unsaturated organic substrates.^228^ Because of these features, H_3_N·BH_3_ has attracted significant attention as an efficient hydrogen carrier and a versatile reagent for metal-free hydrogenation processes, enabling diverse synthetic applications in modern organic chemistry.^228–230^

In 2016, Du and co-workers reported that a chiral FLP consisting of HB­(C_6_F_5_)_2_ (**B** ^**60**^)^231^ (10 mol %) and (*S*)-*tert*-butylsulfinamide (10 mol %), in combination with H_3_N·BH_3_ as the reductant, efficiently promoted the asymmetric transfer hydrogenation of imines under mild conditions, affording the corresponding chiral amines in high yields.^232^ Optimal performance was achieved in toluene at ambient temperature, furnishing a broad range of chiral amines with excellent enantioselectivities. The protocol exhibited a wide substrate scope, efficiently accommodating various imines, including aryl-, heteroaryl-, and aliphatic derivatives. Mechanistic investigations identified an eight-membered-ring transition state responsible for stereocontrolled hydrogen transfer and the regeneration of the active FLP through H_3_N·BH_3_ mediated hydride delivery. The catalytic cycle is initiated by the association of the borane with the chiral sulfinamide to generate a reactive FLP, which activates the imine substrate and facilitates hydrogen transfer, ultimately affording the corresponding amine along with a boron–nitrogen intermediate (Scheme 51).

**Scheme 51:**
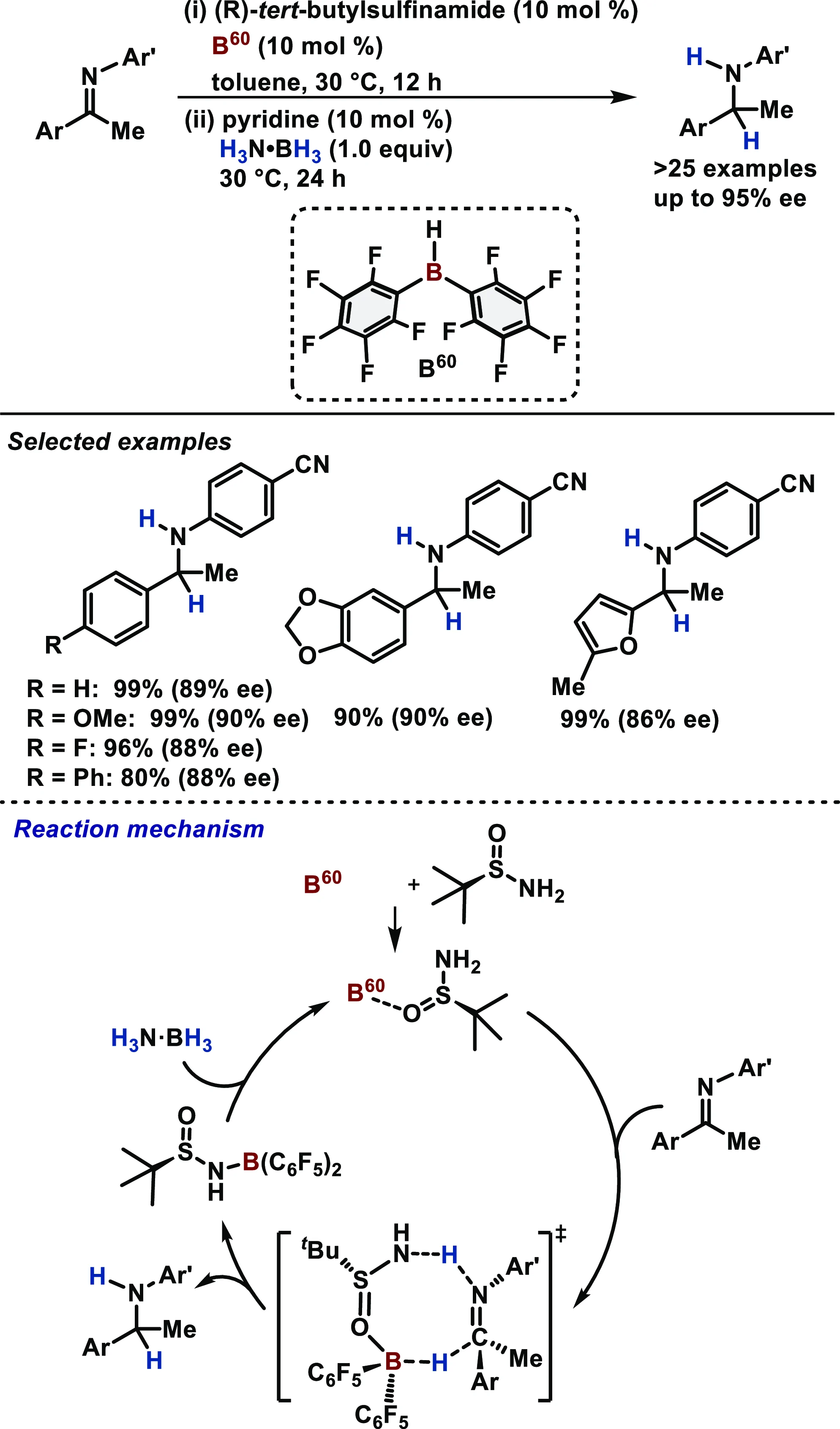
**B** ^**60**^-Catalyzed Hydrogenation of Imines Using H_3_N·BH_3_

The same authors reported a borane-catalyzed transfer hydrogenation of pyridines using H_3_N·BH_3_, providing a practical alternative to conventional high-pressure hydrogenation.^233^ The catalytic system employed **B** ^**1**^ or *in situ*-generated boranes to activate H_3_N·BH_3_, generating reactive species capable of reducing pyridines. Mechanistically, heterolytic cleavage of the B–H and N–H bonds of H_3_N·BH_3_ by an FLP comprising pyridines and **B** ^**1**^ results in hydride and proton transfer to the pyridinium intermediate, ultimately yielding piperidines. Optimization studies revealed that reaction concentration, catalyst loading, and solvent selection significantly influence both the reactivity and selectivity of the transformation. Under optimized conditions, toluene at 120 °C proved most effective, enabling complete conversion within 6 h with 10 mol % **B** ^**1**^ in the presence of H_3_N·BH_3_. The methodology demonstrated a broad substrate scope, enabling efficient reduction of 2,6-diarylpyridines, 2-aryl-6-methylpyridines, and dialkylpyridines to the corresponding piperidines in yields of up to 88% with predominant *cis* selectivity. Notably, sterically demanding and electronically diverse pyridine derivatives were well-tolerated. However, monosubstituted and 2,3-disubstituted pyridines exhibited comparatively lower reactivity (Scheme 52).

**Scheme 52:**
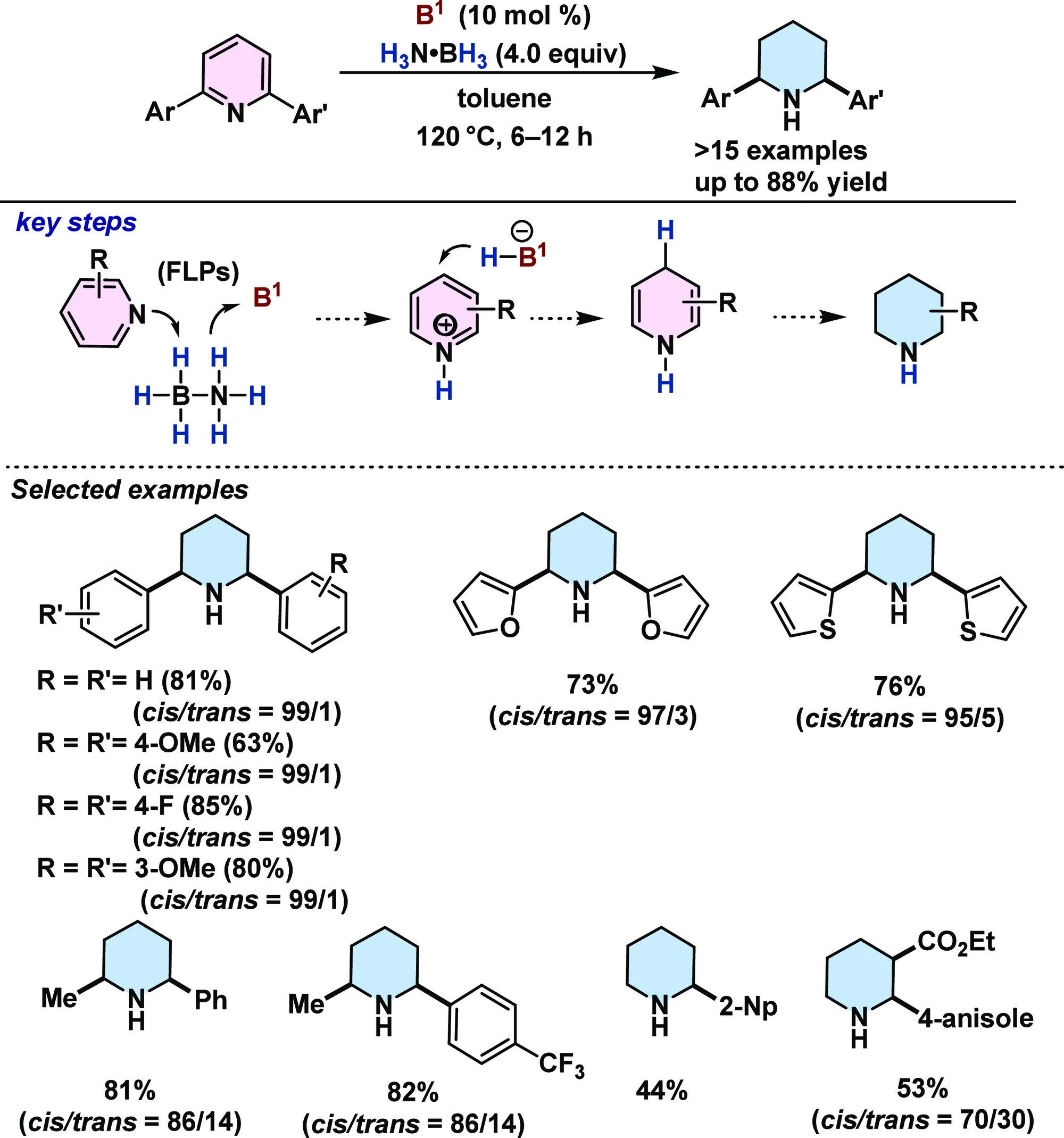
**B** ^**1**^-Catalyzed Hydrogenations of Pyridines with H_3_N·BH_3_

Shi and co-workers reported a metal-free hydrogenation strategy employing the strong Lewis acid **B** ^**1**^ (5 mol %) in combination with H_3_N·BH_3_ as a convenient hydrogen source in toluene at 80 °C.^234^ Solvent screening revealed that toluene provided superior performance compared to CH_3_CN, THF, dioxane, and 1,2-dichloroethane. Under these conditions, the protocol enabled selective hydrogenation of a broad range of six-membered nitrogen-containing heteroarenes, including quinoline-, quinoxaline-, and indole-derived substrates, under relatively mild conditions. Notably, the catalytic system exhibited excellent functional-group tolerance, with halo-substituted substrates, such as bromo and chloro derivatives, affording the corresponding hydrogenated products in moderate to excellent yields (Scheme 53).

**Scheme 53:**
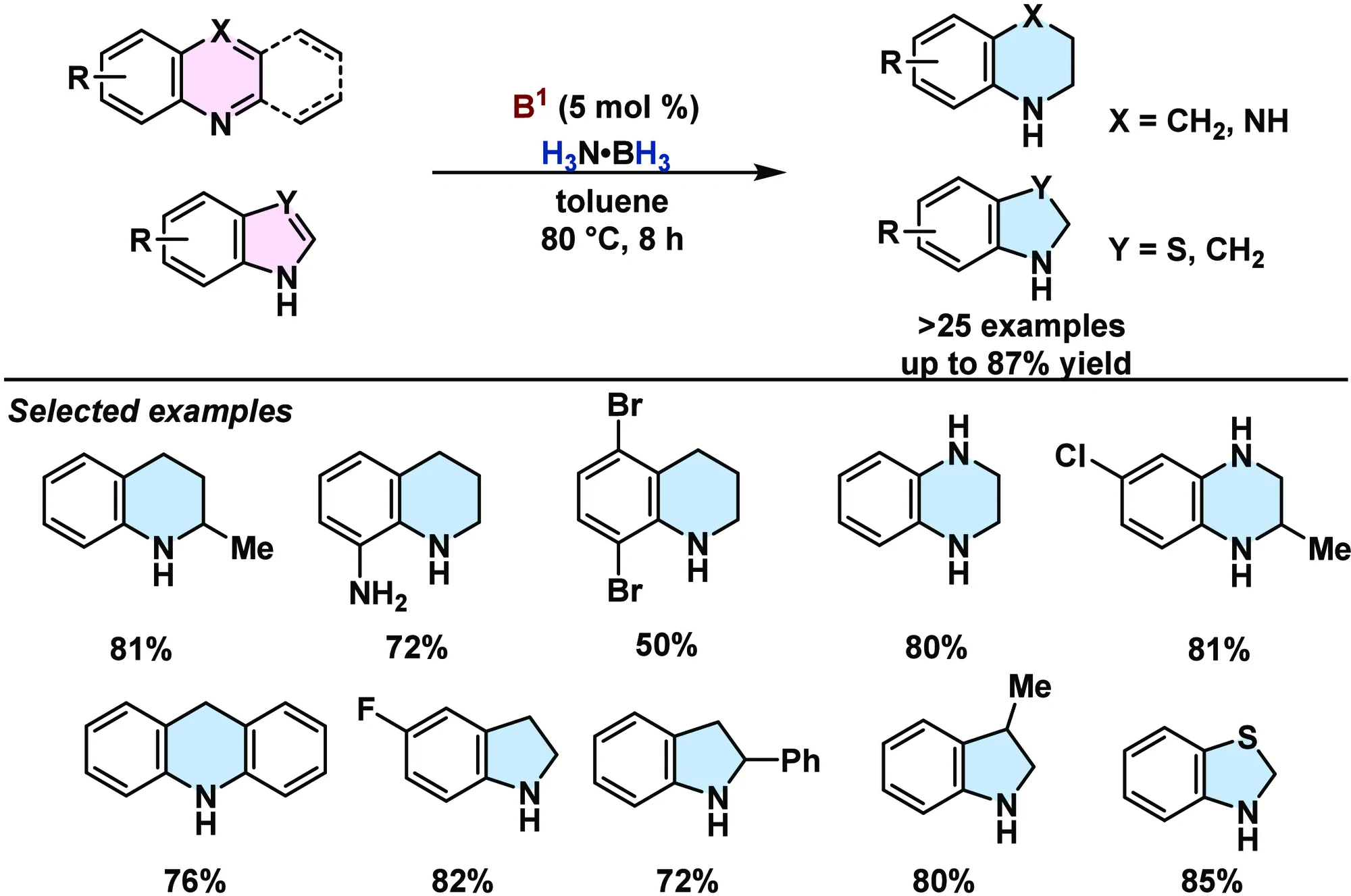
**B** ^**1**^-Catalyzed Hydrogenations of N-Heterocycles with H_3_N·BH_3_

In 2017, Du and co-workers reported an efficient protocol for the asymmetric transfer hydrogenation of 2,3-disubstituted quinoxalines using H_3_N·BH_3_ (2.0 equiv) as the hydrogen source in toluene at 30 °C, employing **B** ^**60**^ (10 mol %) in combination with (*R*)-*tert*-butylsulfinamide (20 mol %) as a chiral catalyst.^235^ The catalytic process proceeded via a synergistic activation pathway, in which the borane coordinates to and activates the heteroaromatic substrate, whereas the chiral sulfinamide governs enantioselectivity and facilitates proton transfer from H_3_N·BH_3_. The study revealed distinct stereochemical outcomes depending on the substitution pattern of the quinoxaline substrates. 2-Alkyl-3-arylquinoxalines predominantly afforded *cis*-tetrahydroquinoxalines in good yields with up to 86% ee, whereas 2,3-dialkylquinoxalines mainly produced *trans* products with excellent stereocontrol (>99% ee). Optimization studies further showed that increased catalyst loading and mixed solvent systems improved both conversion and enantioselectivity. Collectively, these results emphasize the important role of H_3_N·BH_3_ as a practical hydrogen donor in borane-catalyzed metal-free reduction chemistry (Scheme 54).

**Scheme 54:**
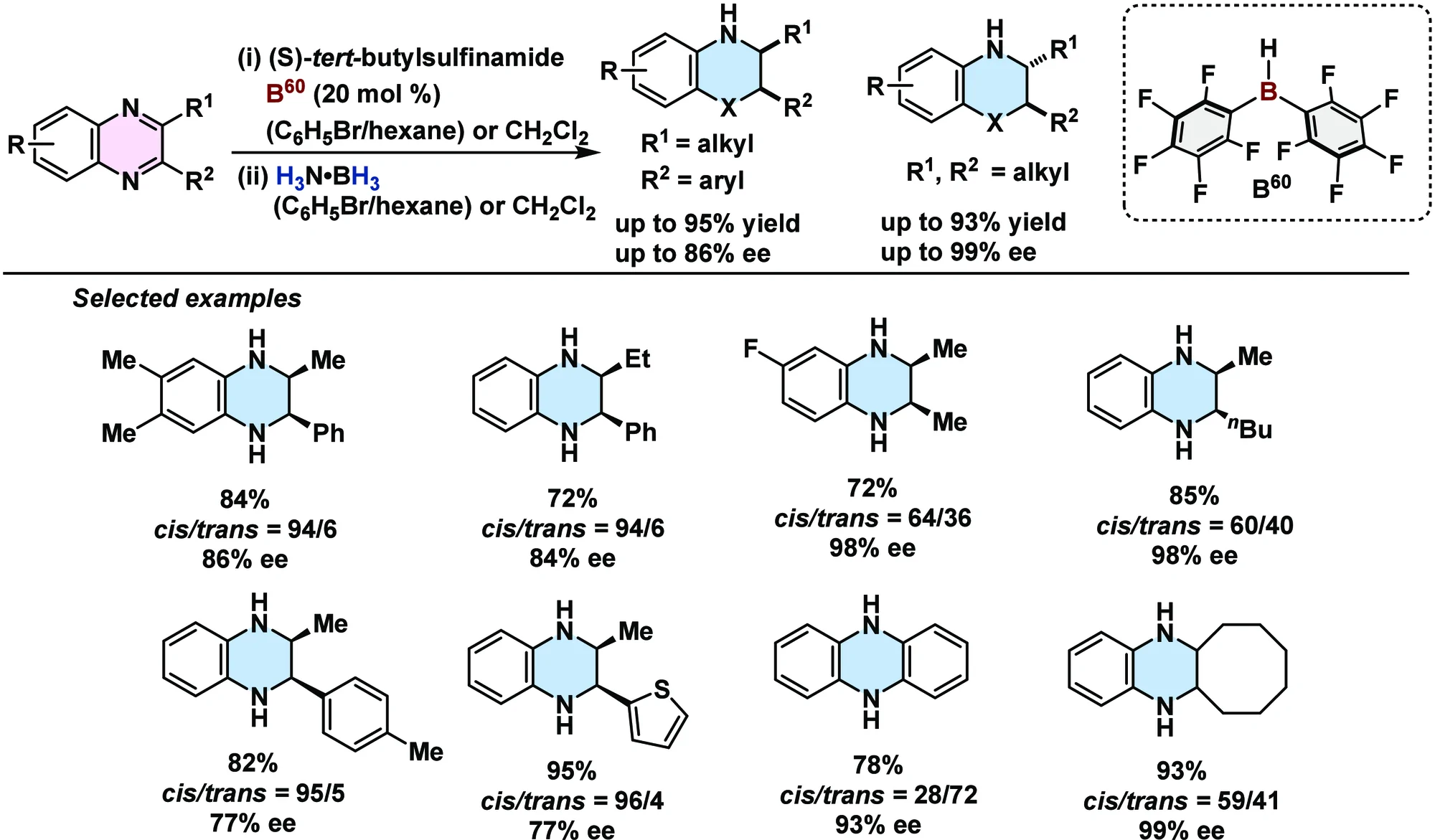
Hydrogenation of 2,3-Disubstituted Quinoxalines with H_3_N·BH_3_

Zhong and co-workers reported **B** ^**1**^-catalyzed asymmetric reductive amination of ketones, employing α-methylbenzylamine (α-MBA) as a chiral auxiliary and H_3_N·BH_3_ as the reducing agent.^236^ The method exhibited excellent functional-group tolerance, efficiently converting a broad range of substrates, including aryl ketones bearing electron-donating or electron-withdrawing substituents, heteroaryl ketones such as pyridine- and benzofuran-derived systems, as well as sterically congested ketones, into the corresponding amines in excellent yields with high stereoselectivity. Control experiments revealed that moisture significantly inhibits the reaction. The addition of 10 mol % water decreased the yield to 58%, whereas conducting the reaction in the presence of 4 Å molecular sieves afforded the product in 95% yield with 91% de, underscoring the critical importance of maintaining anhydrous conditions (Scheme 55). Mechanistically, the transformation proceeded via a one-pot **B** ^**1**^-catalyzed asymmetric reductive amination pathway. In the presence of water generated during imine formation, **B** ^**1**^ was initially sequestered as the inactive species [**B** ^**1**^–OH]­[H–L], wherein L includes imines and/or amines. The removal of water by 4 Å molecular sieves restored a critical concentration of free **B** ^**1**^, which facilitated ketone–amine condensation through B···O coordination, leading to the formation of a *trans*-imine intermediate **B**. Concurrently, **B** ^**1**^ activated H_3_N·BH_3_ to generate a reactive ion pair, followed by protonation of the imine to form an iminium species. At low reaction temperatures, *trans*-to-*cis* imine isomerization was suppressed, thereby enabling high diastereocontrol. Subsequent hydride transfer to the less hindered *Re* face of the iminium intermediate furnished the amine product with high diastereoselectivity (Scheme 55).

**Scheme 55:**
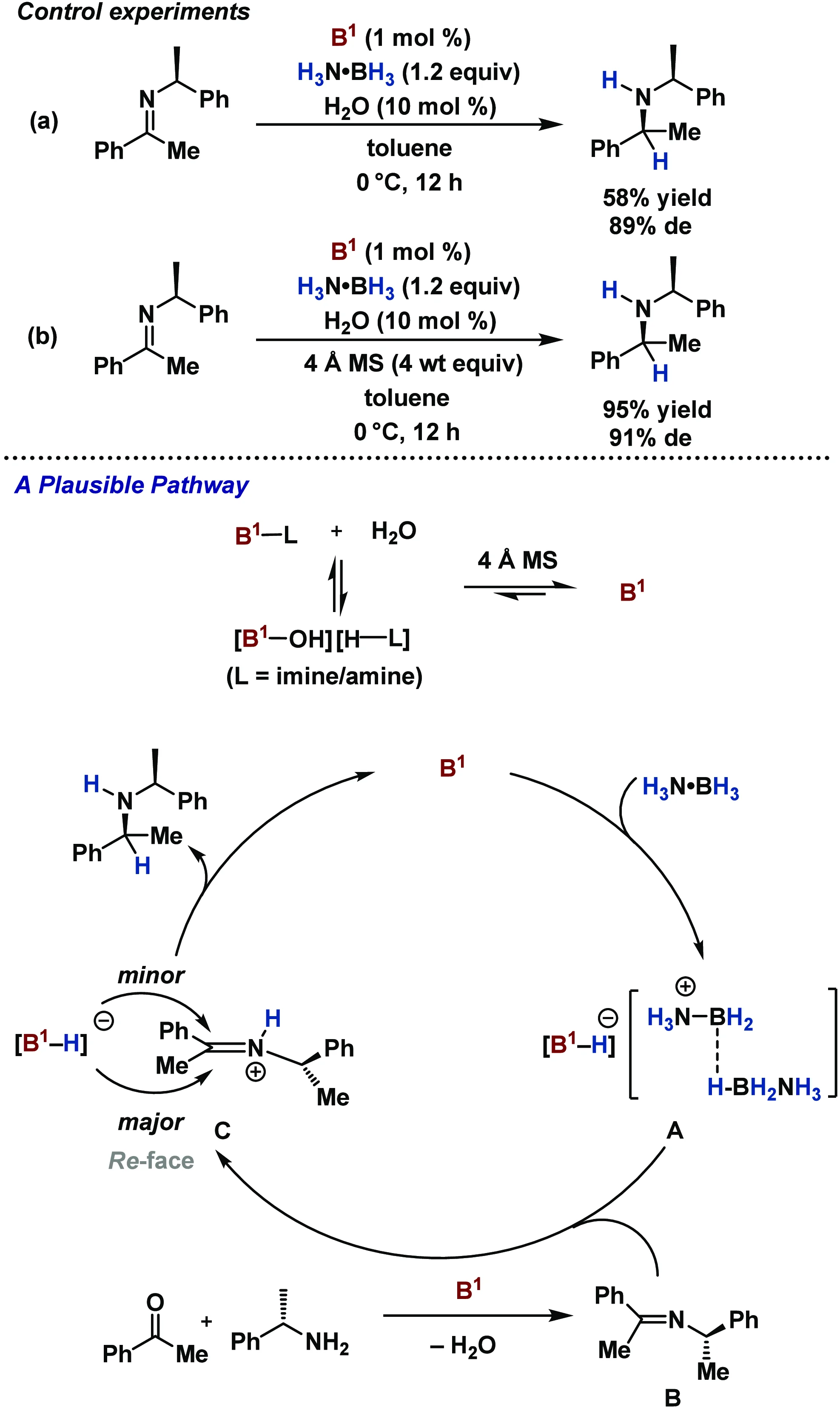
**B** ^**1**^-Catalyzed Reductive Amination of Ketones with H_3_N·BH_3_

Du and co-workers reported an asymmetric transfer hydrogenation of β-N-substituted enamino esters employing H_3_N·BH_3_ as the hydrogen donor, catalyzed by **B** ^**60**^ in combination with (*S*)-*tert*-butylsulfinamide.^237^ The protocol afforded a diverse array of optically active β-amino acid derivatives in very good yields, with enantioselectivities up to 91% ee. The reaction exhibited broad substrate tolerance, accommodating both electron-donating and electron-withdrawing substituents on the aromatic ring. Notably, β-enamino ethyl esters bearing *ortho*-substituted phenyl groups were also compatible substrates, although they afforded the corresponding products with comparatively lower yields and enantioselectivity. The effect of the ester functionality was also investigated, showing that increasing steric bulk on the alkyl substituent markedly influenced enantioselectivity, yielding ee values up to 91%. The *tert*-butyl ester, being the most sterically demanding, afforded the highest level of enantioselectivity (Scheme 56).

**Scheme 56:**
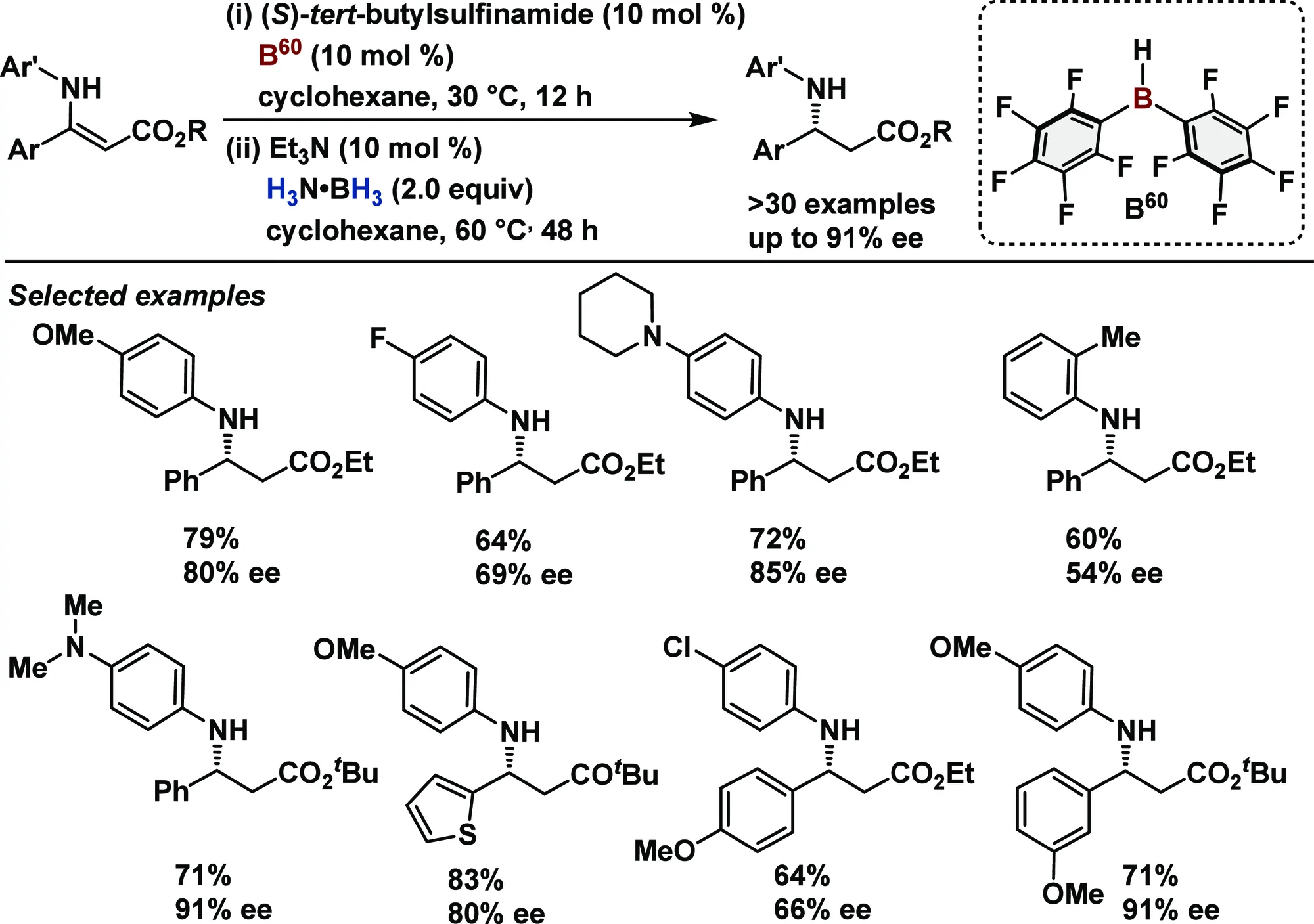
Hydrogenation of N-Substituted Enamino Esters with H_3_N·BH_3_

The same authors subsequently reported an asymmetric transfer hydrogenation protocol for N-unprotected indoles using a chiral catalyst system composed of (*S*)-*tert*-butylsulfinamide (30 mol %) and **B** ^**60**^ (10 mol %), in conjunction with H_3_N·BH_3_ (2.0 equiv) as the hydrogen donor.^238^ The transformation, conducted in cyclohexane at 60 °C for 48 h, efficiently converted a broad range of substituted indoles into the corresponding chiral indolines, affording the products in good yields with enantioselectivities of up to 90% ee. The methodology displayed good substrate generality, accommodating indoles bearing either electron-donating or electron-withdrawing substituents at different positions. However, *N*-methyl-protected indole exhibited markedly reduced reactivity and enantioselectivity, whereas sterically demanding or electronically deactivated substrates generally led to lower conversions.

In 2019, Xiao and co-workers reported a **B** ^**1**^-catalyzed protocol for the deoxygenative reduction of structurally diverse amides to the corresponding amines, employing bench-stable and readily accessible H_3_N·BH_3_ as the hydrogen source.^239^ The transformation proceeds under mild conditions at 60 °C in 1,2-dichloroethane and exhibits a broad substrate scope, affording a wide range of synthetically valuable amines in high yields with good functional group tolerance. The catalytic system comprises **B** ^**1**^ as the primary catalyst and BF_3_·OEt_2_ as a cocatalyst. BF_3_·OEt_2_ promotes amide activation via *in situ* formation of a Lewis acid–base adduct with the carbonyl oxygen, thereby enhancing electrophilicity and facilitating reduction. Mechanistically, **B** ^**1**^ activates the amide carbonyl via coordination, thereby increasing its electrophilicity. Subsequent hydride transfer from H_3_N·BH_3_ generates a hemiaminal intermediate, which undergoes further activation and sequential reduction steps, culminating in C–O bond cleavage and formation of the amine product. The catalytic cycle is facilitated by transient boron–hydride species that mediate efficient hydrogen transfer processes (Scheme 57).

**Scheme 57:**
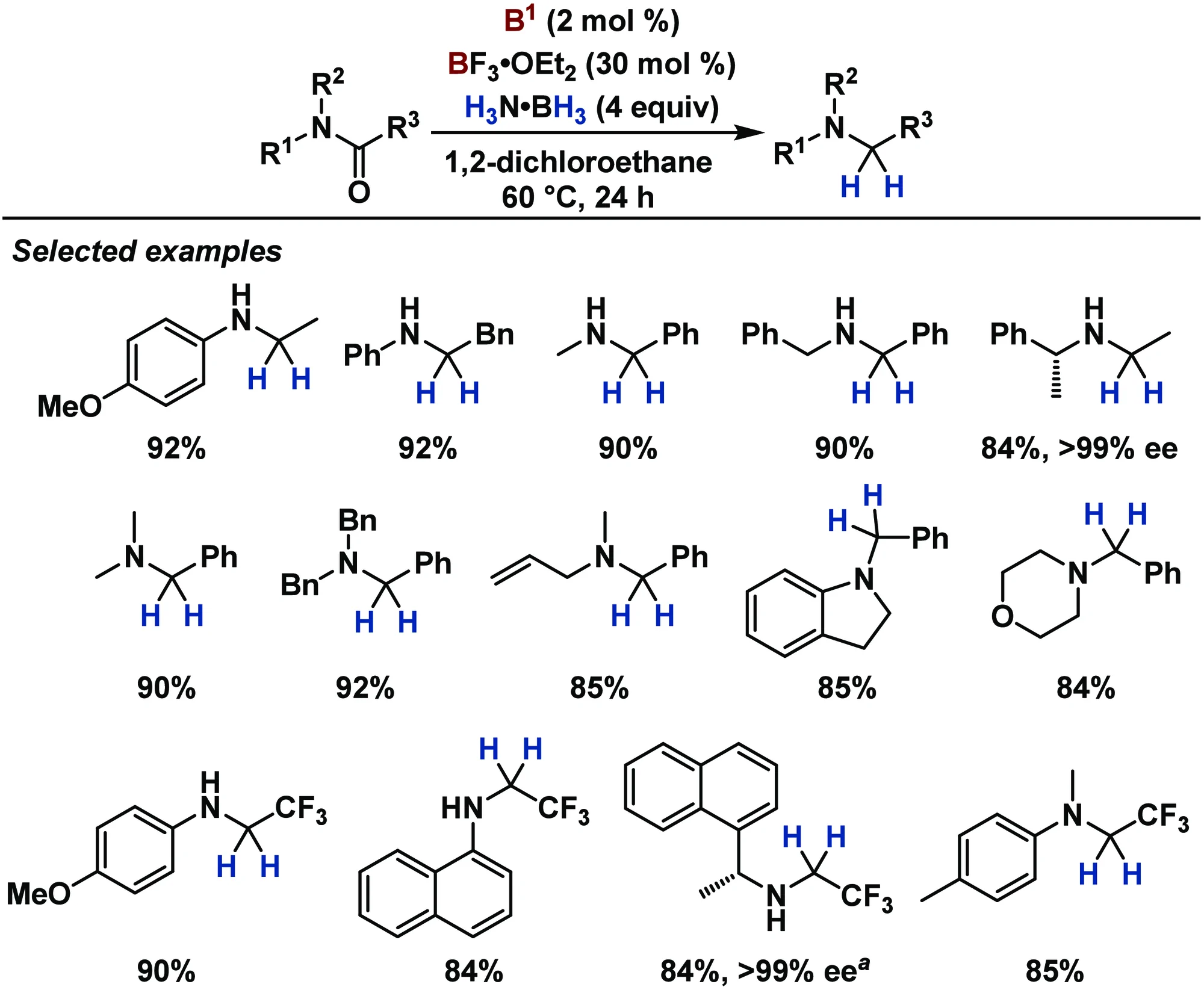
**B** ^**1**^-Catalyzed Deoxygenative Reduction of Amides to Amines with H_3_N·BH_3_

a Starting material (>99% ee) was used.

In 2022, Mejía and co-workers reported that **B** ^**1**^, in combination with BF_3_·OEt_2_ as a cocatalyst, efficiently promoted the catalytic transfer hydrogenation of organic carbonates to the corresponding alcohols.^240^ The reaction proceeded efficiently in 1,2-dichloroethane at 55 °C and exhibited a broad substrate scope, including aromatic, aliphatic, and heteroaromatic carbonates. Under these mild conditions, a variety of carbonate derivatives were selectively reduced to the corresponding primary alcohols in high yields. Mechanistic investigations indicated that **B** ^**1**^ activated both the carbonate and H_3_N·BH_3_ through Lewis acid–base interactions, promoting hydride transfer via a borane-catalyzed reduction pathway. The reaction likely proceeded via sequential hydride transfer, yielding a tetrahedral intermediate, which subsequently underwent C–O bond cleavage to afford the corresponding alcohol along with a boron-containing byproduct (Scheme 58).

**Scheme 58:**
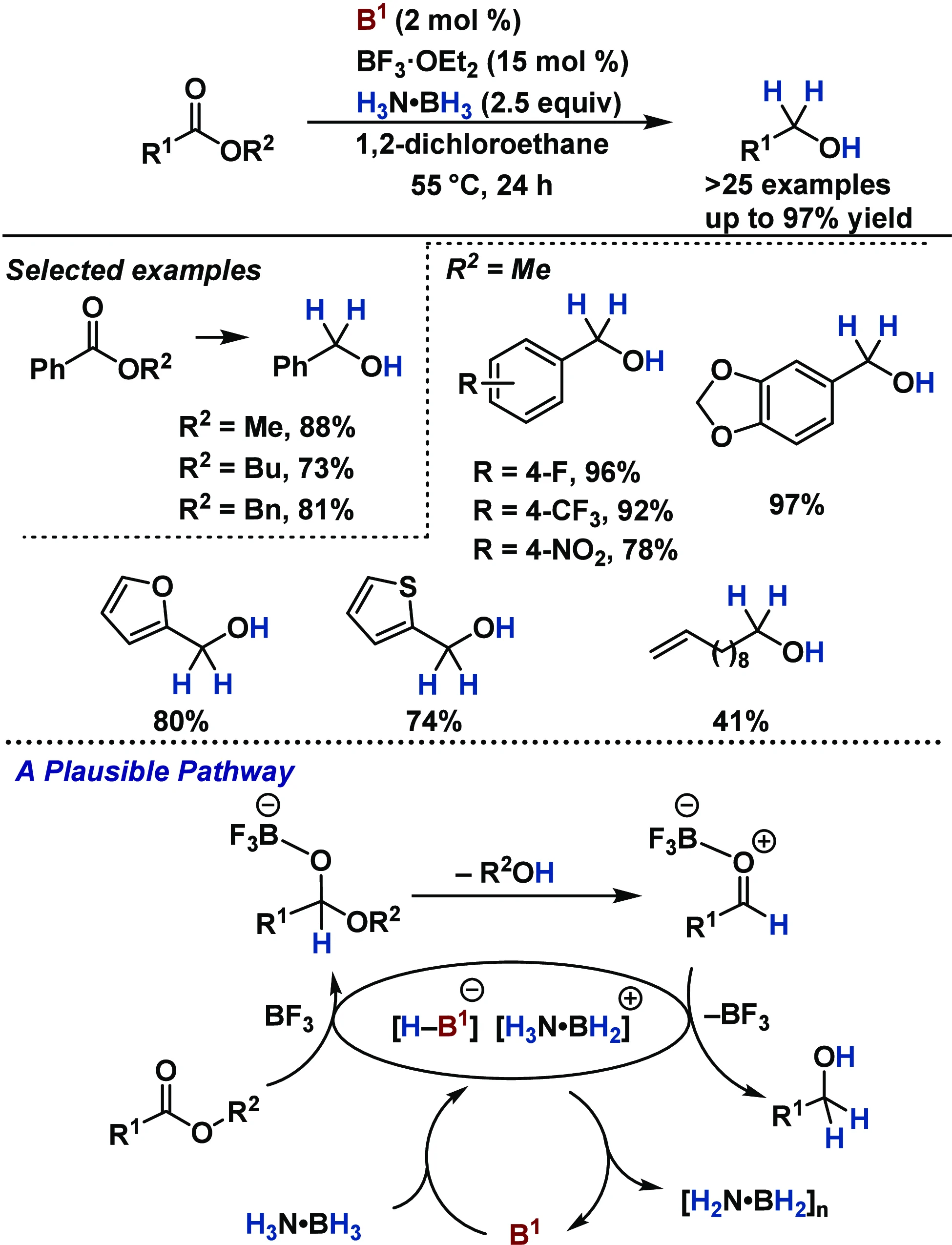
**B** ^**1**^-Catalyzed Hydrogenation of Esters Towards Alcohols with H_3_N·BH_3_

## 7 Triarylborane-Catalyzed Transformations Using Cyclohexadienes

Cyclohexadiene has emerged as an effective organic reductant in modern synthetic chemistry, owing to its ability to function as a surrogate for gaseous H_2_. Consequently, cyclohexadiene-mediated transfer hydrogenation has gained considerable attention as a practical and atom-economical alternative to conventional reducing agents. These features underscore the growing significance of cyclohexadiene as a versatile reductant in organic synthesis, particularly in borane-catalyzed reductive transformations.^241,242^

Cyclohexadienes, particularly parent 1,4- and 1,3-cyclohexadienes, offer several practical advantages over molecular hydrogen and hydrosilanes as hydrogen donors. Unlike H_2_ gas, both are stable liquids that can be handled under ambient conditions without specialized high-pressure equipment, providing improved operational safety and convenience, particularly on a laboratory scale.^5^ Of note, this is especially valuable when the cyclohexadiene platform is used in surrogates for low-molecular-weight hydrosilanes such as Me_3_SiH^244^ and SiH_4_,^256^ which are delicate gases that are difficult to handle in the laboratory. In addition, hydrogen transfer from cyclohexadienes occurs under catalytic conditions in a controlled manner, thereby enhancing chemoselectivity and suppressing the overreduction of sensitive functional groups. Compared with hydrosilanes, cyclohexadienes also avoid generating silicon-containing waste, producing benzene as the sole stoichiometric byproduct, which can be readily removed from the reaction mixture.

In 2015, Sakata and Fujimoto carried out a quantum-chemical study on the reactivity of 3-(trimethylsilyl)­cyclohexa-1,4-diene in the presence of **B** ^**1**^, suggesting that **B** ^**1**^ could abstract a hydride from C6 of 3-(trimethylsilyl)­cyclohexa-1,4-diene to generate a highly polarized ion pair, [SiMe_3_(C_6_H_6_)]^+^/[H**B** ^**1**^]^−^.^243^ In dichloromethane, this species would exist as either dissociated ions or a weakly associated ion pair. Subsequent structural reorganization with the concomitant formation of benzene affords the key reactive complex Me_3_Si–H–**B** ^**1**^, which mediates the hydrosilylation reaction. Computational studies indicated that formation of the key reactive complex was less favorable in toluene and benzene, and became highly unfavorable in *n*-pentane. The observed anti-Markovnikov selectivity was rationalized by the relative nucleophilicity of the alkene carbons and the enhanced Lewis acidity of the silicon center. The proposed mechanism is consistent with the experimental findings reported by Simonneau and Oestreich,^244^ although additional minor pathways may also operate.

In the same year, Oestreich and co-workers systematically investigated **B** ^**1**^-catalyzed hydrosilylation, demonstrating its effectiveness in both direct and transfer processes.^245^ Electron-deficient boranes activate Si–H bonds via borohydride–silylium ion pairs, enabling hydride transfer to unsaturated substrates. In transfer hydrosilylation, cyclohexadiene derivatives act as hydride sources, generating silylium species through aromatization. Hydrosilane release proceeds via hydride abstraction to form a silicon-stabilized Wheland intermediate, followed by borohydride capture. The resulting hydrosilanes react with π- and σ-donating substrates. Structure–reactivity studies revealed that both steric and electronic factors, beyond Lewis acidity, critically influence catalytic efficiency and selectivity.

Oestreich and co-workers further reported a **B** ^**1**^-catalyzed transfer hydrogenation of imines and related heteroarenes using substituted cyclohexa-1,4-dienes as surrogates for molecular dihydrogen.^246^ The reduction of imines efficiently proceeded at 125 °C with catalyst loadings of 10–15 mol % in toluene. The catalytic cycle was initiated by the rate-limiting Lewis acid–mediated hydride abstraction from cyclohexadiene, generating the counteranion [H–**B** ^**1**^]^−^ in low concentration. The substrate scope was limited to specific N-protecting groups that provided an optimal balance of steric hindrance, adequate Lewis basicity, and thermodynamic stability, thereby facilitating efficient hydride transfer while suppressing undesired side reactions. The protocol exhibited broad functional-group tolerance, accommodating a variety of functionalized ketimines and aldimines. Substrates bearing electron-withdrawing substituents, as well as sterically demanding *ortho*-substituted aryl groups, were well-tolerated. Furthermore, nitrogen-containing heterocycles also participated efficiently in the **B** ^**1**^-catalyzed transfer hydrogenation, furnishing the reduced products in high yields (Scheme 59). Later, in 2016, the same group reported a Brønsted acid-catalyzed transfer hydrogenation of imines using Tf_2_NH (15 mol %) and cyclohexa-1,4-dienes as dihydrogen surrogates.^247^

**Scheme 59:**
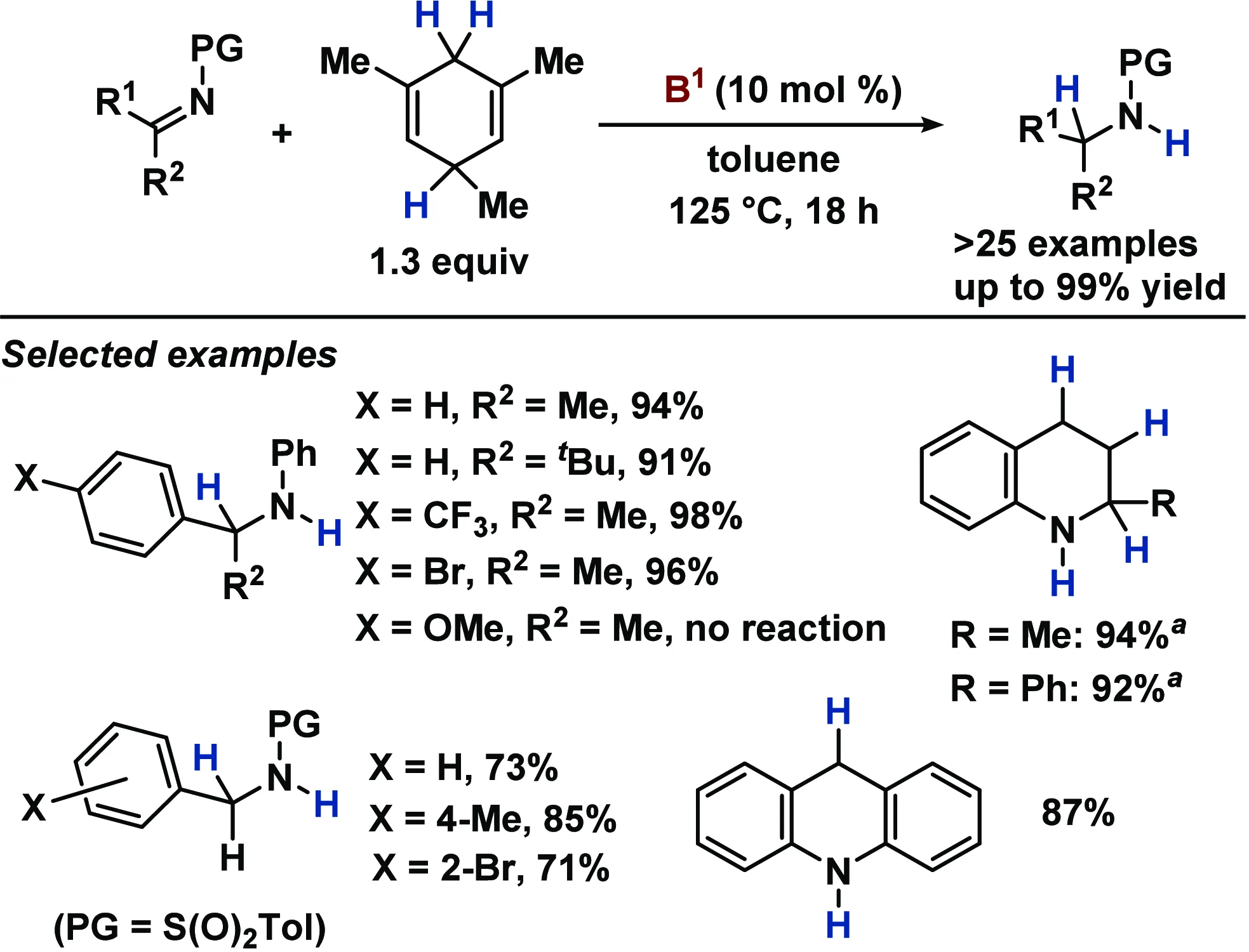
**B** ^**1**^-Catalyzed Reduction of Imine Substrate with Cyclohexadiene

a 2.6 equiv of cyclohexadiene.

The same group developed a method for a **B** ^**1**^-catalyzed transfer hydrogenation, enabling the formal transfer of dihydrogen between unsaturated hydrocarbons under mild conditions at room temperature.^248^ The process required an additional methyl group at the bisallylic position of the cyclohexadienyl moiety to facilitate selective hydride abstraction and suppress competing pathways, including dihydrogen evolution and heterodimerization of the cationic intermediates. The reaction exhibited broad substrate tolerance toward a variety of 1,1-disubstituted alkenes and also extended to trisubstituted derivatives. In particular, 1,1-diarylalkenes afforded the corresponding alkanes in high yields. Moreover, alkyl-substituted styrenes as well as 1,1-dialkylalkenes were well-tolerated, including sterically demanding substituents such as isopropyl and cyclohexyl groups. The process was initiated by **B** ^**1**^-catalyzed hydride abstraction from the dihydrogen surrogate, forming a Brønsted acidic Wheland complex and [H–**B** ^**1**^]^−^. The resulting [H–**B** ^**1**^]^−^ then served as a hydride donor to reduce a second unsaturated hydrocarbon acceptor, thereby completing a catalytic hydrogen-transfer cycle. Mechanistic studies, supported by DFT calculations, indicated that the reaction proceeded via ion-pair intermediates characteristic of FLP reactivity (Scheme 60).

**Scheme 60:**
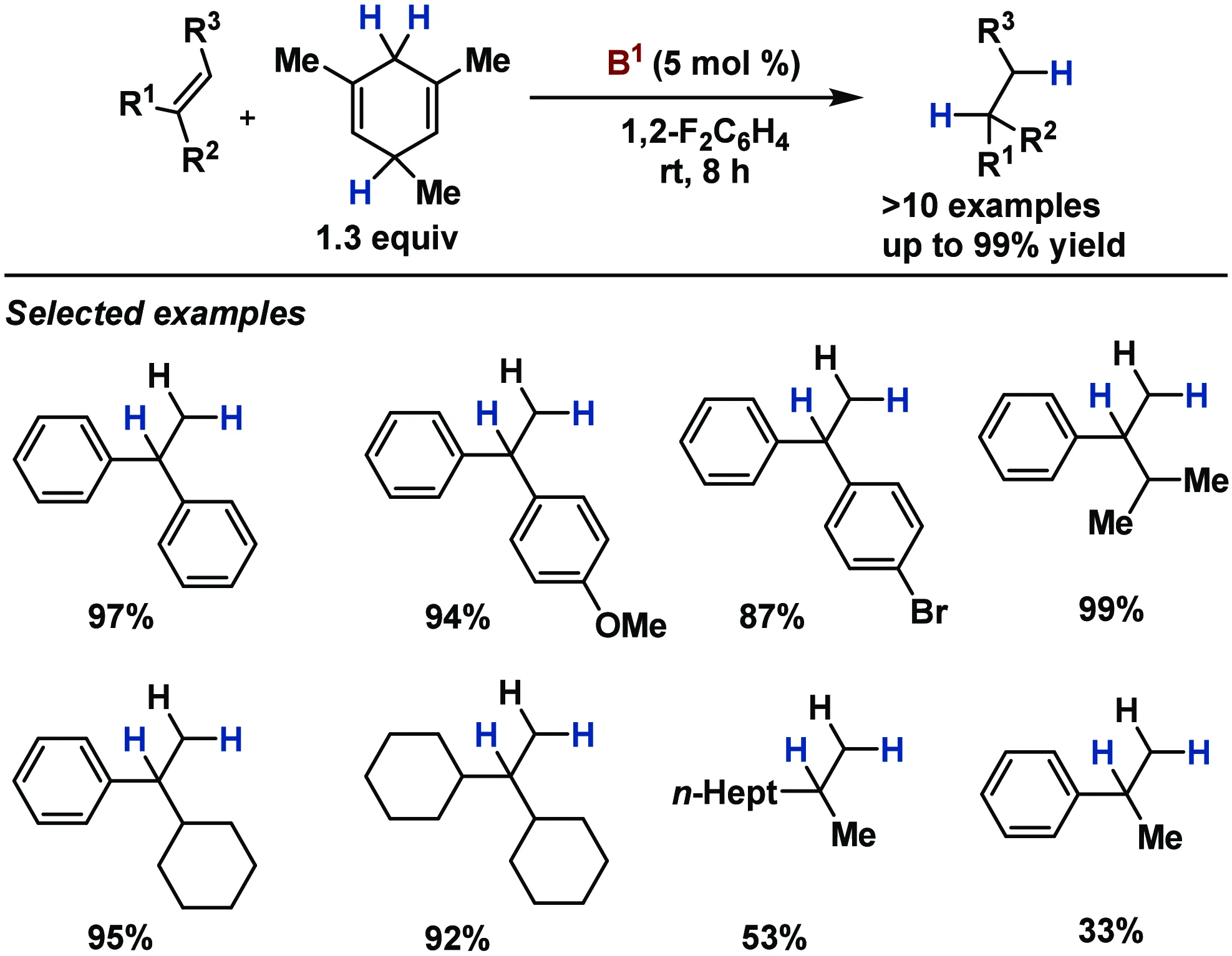
**B** ^**1**^-Catalyzed Hydrogen Transfer with Cyclohexadiene

In 2017, Oestreich and co-workers reported a **B** ^**1**^-catalyzed transfer reduction strategy employing cyclohexa-1,3-diene derivatives as surrogates for H_2_ and hydrosilanes, thereby enabling efficient transfer hydrogenation and transfer hydrosilylation of alkenes under mild reaction conditions.^249^ The catalytic process proceeded via Lewis acid-mediated hydride abstraction from the diene surrogate, generating a borohydride species that subsequently delivered a hydride to the activated alkene substrate. The substrate scope encompassed 1,2-diphenylethylene derivatives, which reacted in good yields substrates bearing electron-donating and -withdrawing substituents were well-tolerated, although halogenated substrates exhibited slightly reduced reactivity. In addition to 1,1-diarylalkenes, 1-alkyl-1-arylalkenes also participated efficiently, and even a trisubstituted alkene was successfully converted, albeit in a moderate yield. Overall, the work highlighted the versatility of cyclohexa-1,3-diene frameworks as transfer reagents in borane-catalyzed reductions (Scheme 61).

**Scheme 61:**
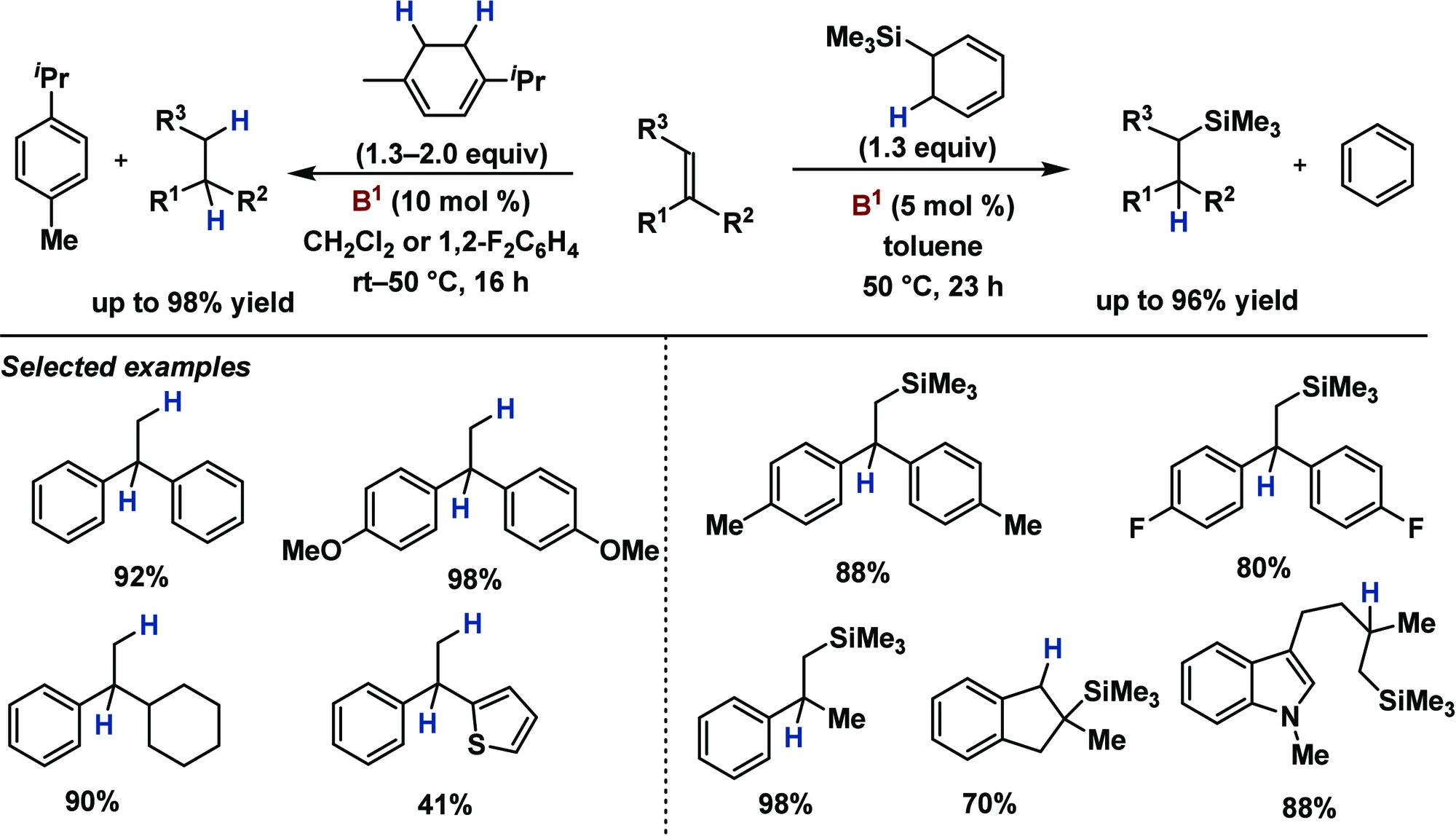
**B** ^**1**^-Catalyzed Hydrogenation and Silyl Transfer Processes with Cyclohexadiene

In the same year, the same group reported a **B** ^**1**^-catalyzed hydro-*tert*-butylation of alkenes employing 3-*tert*-butyl-substituted cyclohexa-1,4-dienes as isobutane equivalents. The catalytic cycle is initiated by borane-mediated hydride abstraction, which generates a borohydride species and a *tert*-butyl-stabilized carbocation. Subsequent hydride transfer to the alkene, followed by protonation, furnishes the hydro-*tert*-butylated product. The transformation is thermodynamically driven by aromatization to benzene, enabling efficient C–C bond formation.^250^ Later, the same group reported a **B** ^**1**^-catalyzed transfer hydromethallylation of electron-rich styrene derivatives using methallyl-substituted cyclohexa-1,4-dienes as isobutene surrogates.^251^ This protocol enabled the catalytic construction of sterically congested quaternary carbon centers and thus represented a rare example of C­(sp^3^)–C­(sp^3^) bond formation via carbenium ions generated by alkene protonation. Mechanistically, borane-induced hydride abstraction generates a borohydride intermediate along with a resonance-stabilized methallyl cation. The borohydride then transfers hydride to the alkene, forming a nucleophilic carbanion equivalent that is subsequently captured by the methallyl cation to establish the C–C bond. The transformation is driven by aromatization to benzene and proceeds efficiently under mild, metal-free conditions with broad substrate generality (Scheme 62).

**Scheme 62:**
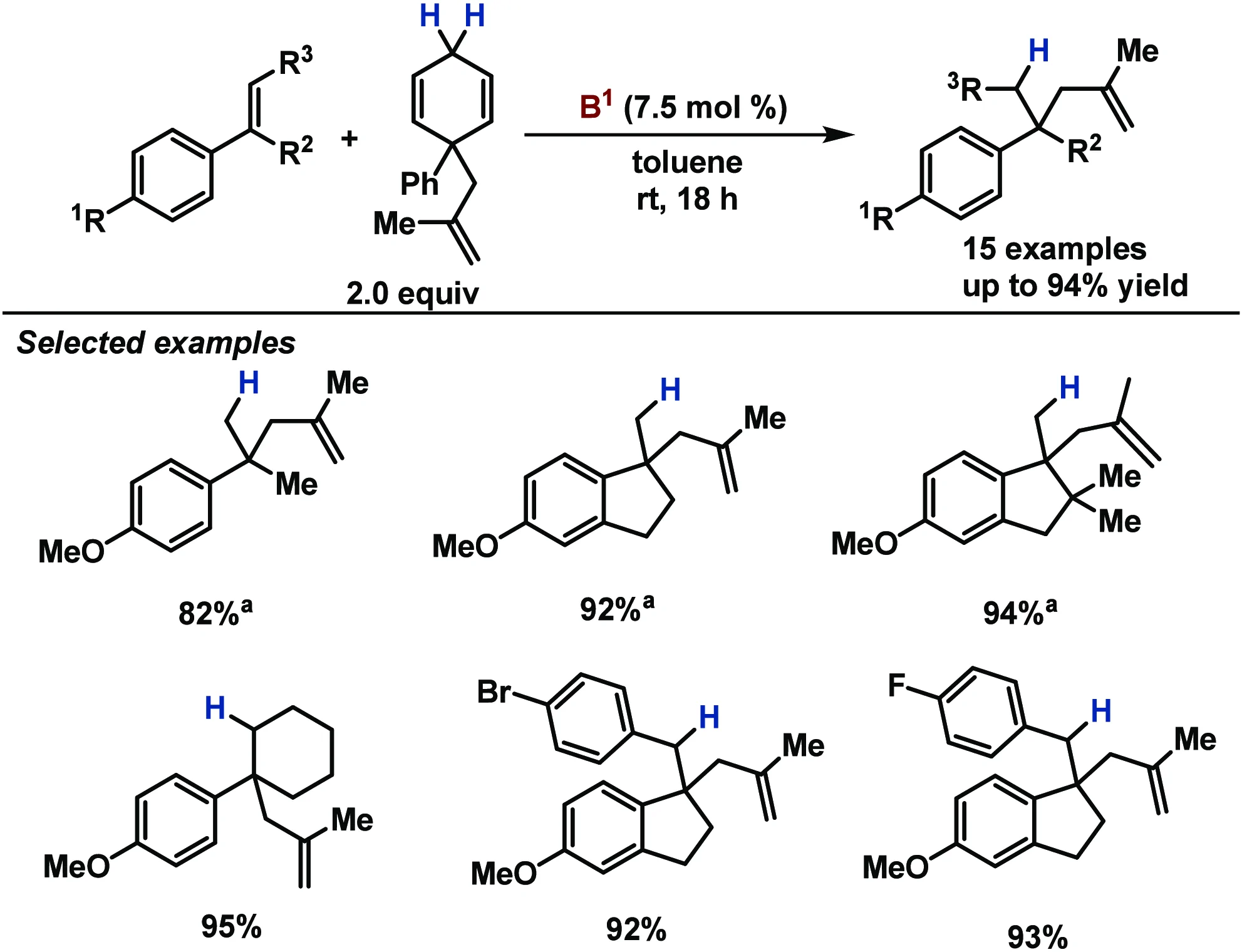
**B** ^**1**^-Catalyzed Transfer Hydromethallylation Using Cyclohexadiene

a 1.3 equiv of cyclohexadiene and 5 mol % **B** ^**1**^.

In 2017, the same group reported a **B** ^**1**^-catalyzed transfer hydrogermylation of alkenes, providing efficient access to fully alkylated germanes at room temperature.^252^ The transformation employed germane surrogates that, upon activation by the Lewis acidic **B** ^**1**^, generated reactive hydrogermane species *in situ* via hydride abstraction from a bisallylic methylene unit, forming an ion pair comprising a germylium/Wheland-type intermediate and [H**B** ^**1**^]^−^. The collapse of this intermediate releases hydrogermane with concomitant benzene formation. Subsequent borane activation of the hydrogermane enabled electrophilic germyl transfer to the π system, while hydride delivery from the borohydride furnished the C–Ge bond and regenerates the catalyst. The transformation is often driven by a favorable thermodynamic process, such as aromatization or formation of stable byproducts (Scheme 63).

**Scheme 63:**
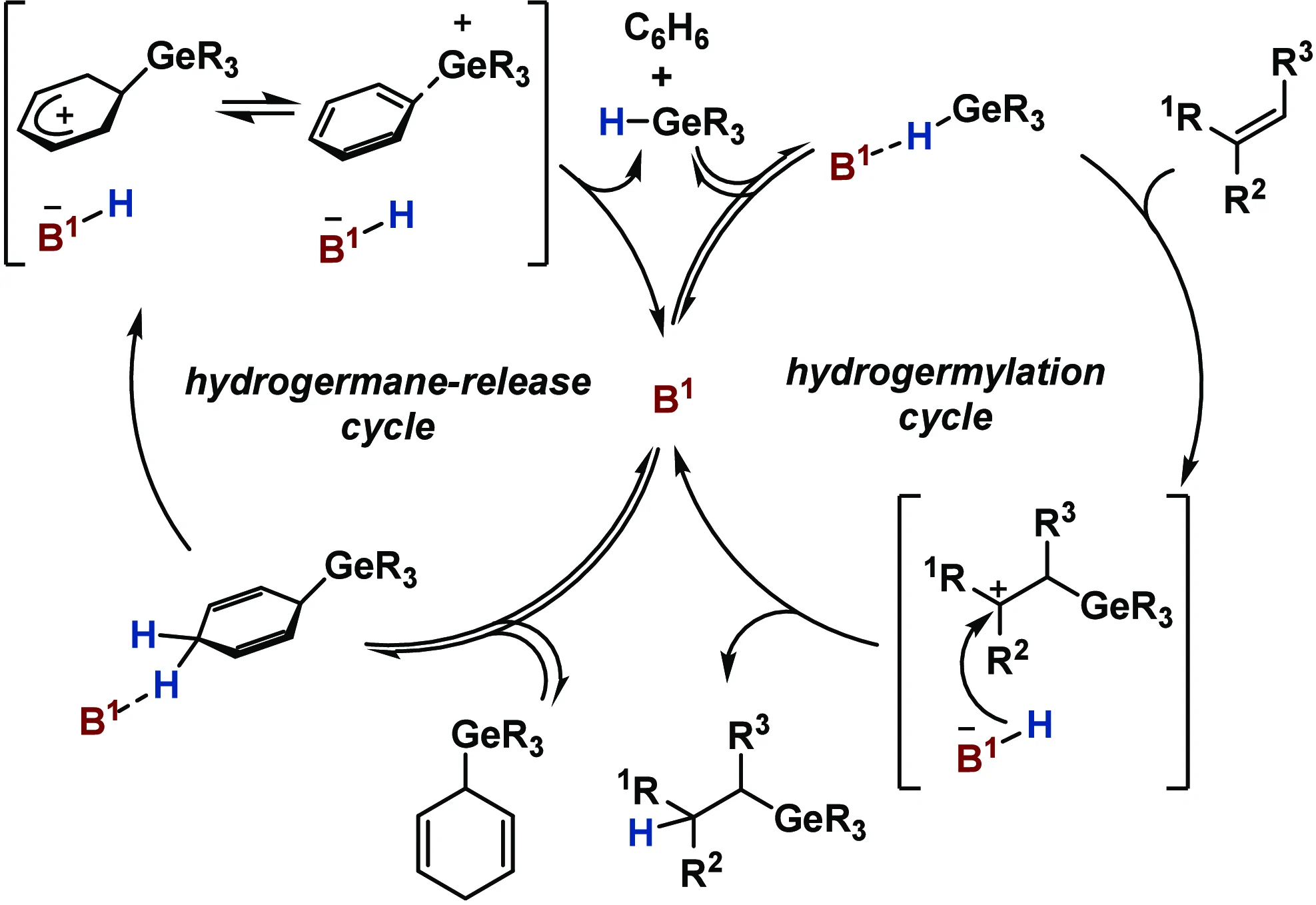
**B** ^**1**^-Catalyzed Transfer Hydrogermylation of Alkenes Using Cyclohexadiene

Oestreich and co-workers reported a selective transfer hydrocyanation of α- and α,β-substituted styrenes using boron Lewis acids, including BCl_3_ and ClB­(C_6_F_5_)_2_ and, in combination with a cyclohexa-1,4-diene-based hydrogen cyanide surrogate. Mechanistically, Lewis acid activation of the cyanide donor generates a reactive ion pair, enabling sequential hydride and cyanide transfer to furnish nitrile products with high selectivity.^253^ Later, in 2022, the same group developed a metal-free decarbonylative transfer hydrochlorination of alkenes and alkynes with a catalytic amount of **B** ^**1**^.^254^ The protocol employed specifically designed acyl chloride surrogates that undergo borane-initiated Grob-type fragmentation to release HCl equivalents and carbon monoxide. Mechanistically, activation of the surrogate by **B** ^**1**^ triggers C–C bond cleavage to generate an ion pair, which subsequently delivers an HCl equivalent across unsaturated C–C bonds. The reaction proceeded with good regioselectivity and accommodated a broad range of alkenes and alkynes. This strategy highlighted the utility of main-group Lewis acids in enabling unconventional transfer hydrofunctionalization pathways driven by fragmentation processes (Scheme 64).

**Scheme 64:**
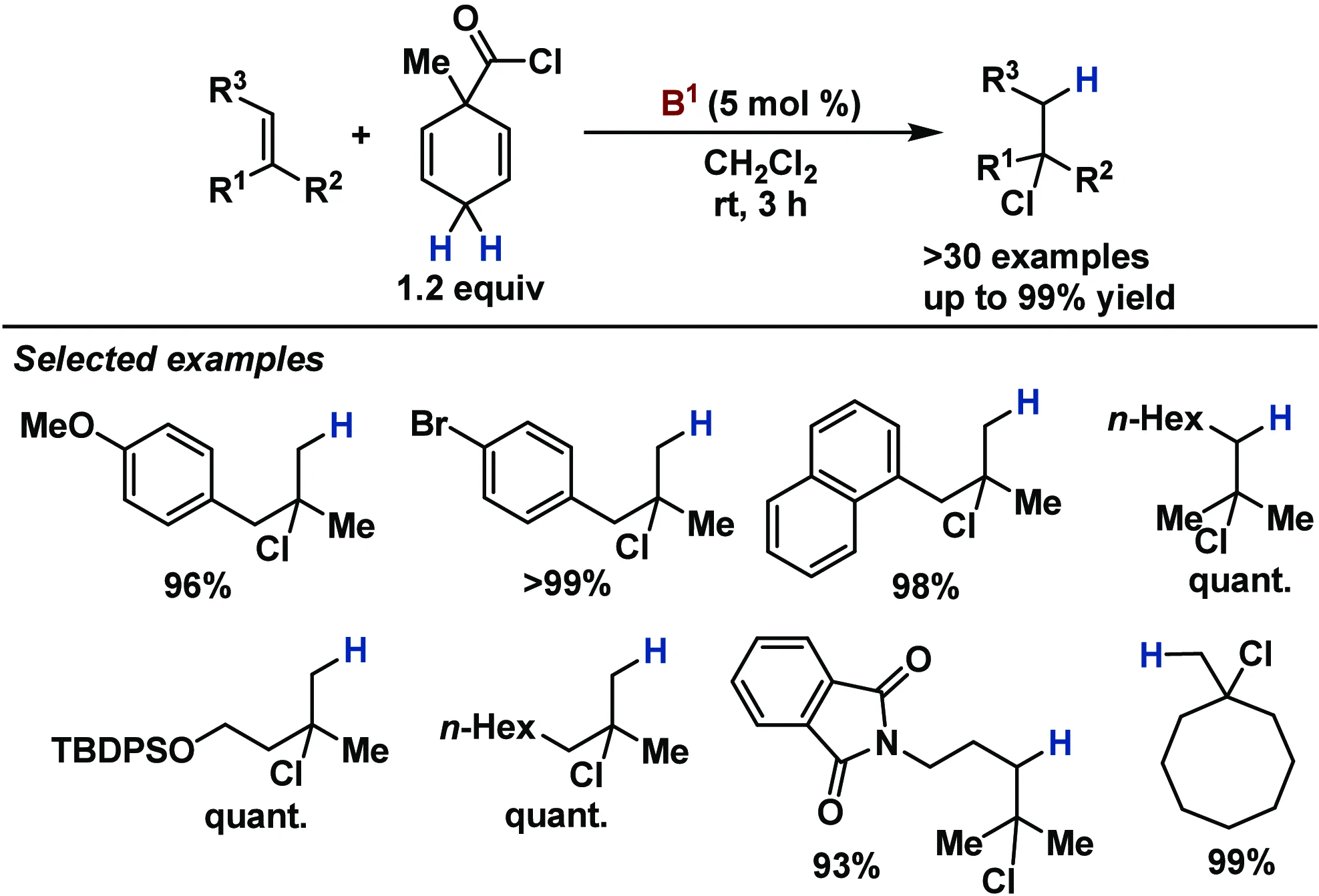
**B** ^**1**^-Catalyzed Transfer Hydrochlorination of Alkenes Using Cyclohexadiene

In 2019, Banerjee and Vanka reported a detailed DFT investigation of cyclohexadiene-based surrogate systems, examining the mechanistic features of the process.^255^ Their study elucidated the significance of the *in situ* generation of SiH_4_, mediated by **B** ^**1**^, from inexpensive and bench-stable precursors, as originally proposed by Simonneau and Oestreich.^256,257^ The entire catalytic cycle, including the previously proposed mechanism, was comprehensively evaluated. Computational results indicated that the active proton source originates from a silicon-based intermediate, consistent with experimental observations in related systems where substituted cyclohexa-1,4-dienes serve as hydride donors.^247,258^ In this study, the authors further examined whether an easily separated ion pair proceeds via an autocatalytic pathway or a **B** ^**1**^-catalyzed mechanism. Using a representative system previously examined experimentally by Chatterjee and Oestreich,^246^ they investigated the hydrogenation of imines, which had been proposed to proceed via **B** ^**1**^-catalysis. Notably, Chatterjee and Oestreich also suggested, in a separate study, the possibility of Brønsted acid-mediated transfer hydrogenation.^247^ In this context, the generation of the iminium cation [(PhMeC)­NH­(Ph)]^+^ could potentially enable an autocatalytic pathway independent of **B** ^**1**^. Thus, a key mechanistic distinction emerges: if the ion pair [(PhMeC)­NH­(Ph)]^+^[H**B** ^**1**^]^−^ readily dissociates, the free iminium cation may promote alternative, nonboron-catalyzed pathways. Conversely, tight ion pairing would favor a **B** ^**1**^-mediated catalytic cycle (Scheme 65).

**Scheme 65:**
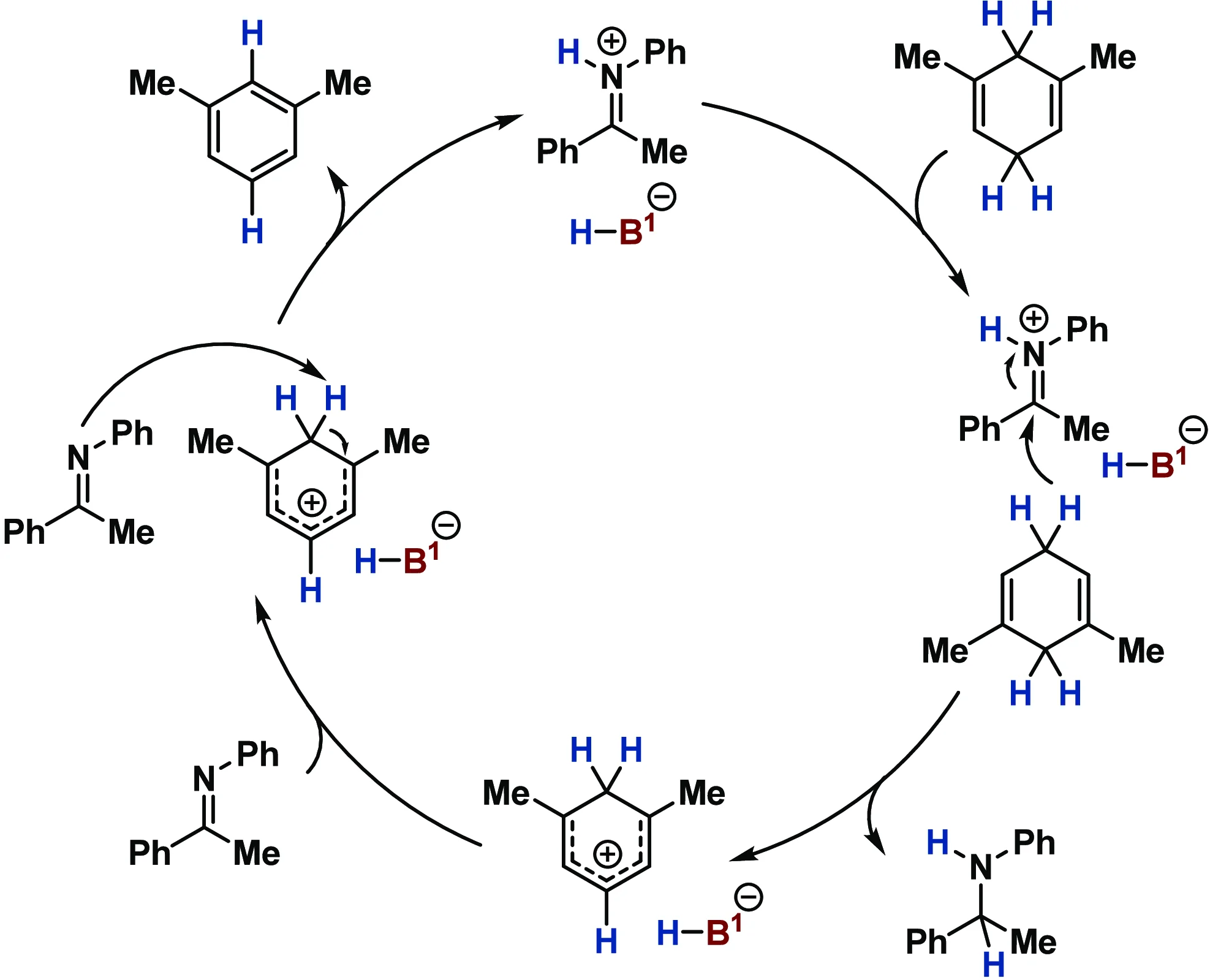
Reaction Mechanism: **B** ^**1**^-Catalyzed Hydrogenation of Imine, Based on the Facile Separation of the Cation from the Ion Pair

In 2018, Walker and Oestreich reported a metal-free **B** ^**1**^-catalyzed regioselective transfer hydrodeuteration of alkenes, employing monodeuterated cyclohexa-1,4-dienes as hydrogen–deuteride (HD) surrogates.^259^ The reaction proceeded efficiently at ambient temperature with low catalyst loading (2.5 mol %), affording hydrodeuterated alkanes in high yields with excellent regioselectivity via a stepwise mechanism initiated by **B** ^**1**^. Initially, deuteride abstraction from the cyclohexadiene surrogate generates an ion pair, I^+^[DB^1^]^−^, consisting of a borodeuteride anion and a highly Brønsted acidic Wheland intermediate. The Wheland intermediate then protonates the alkene, furnishing a carbocationic species, II^+^[D**B** ^**1**^]^−^. Subsequent nucleophilic deuteride transfer from the borodeuteride anion to this carbocation affords the hydrodeuterated product, concomitantly regenerating the **B** ^**1**^ catalyst (pathway 1, Scheme 66).

**Scheme 66:**
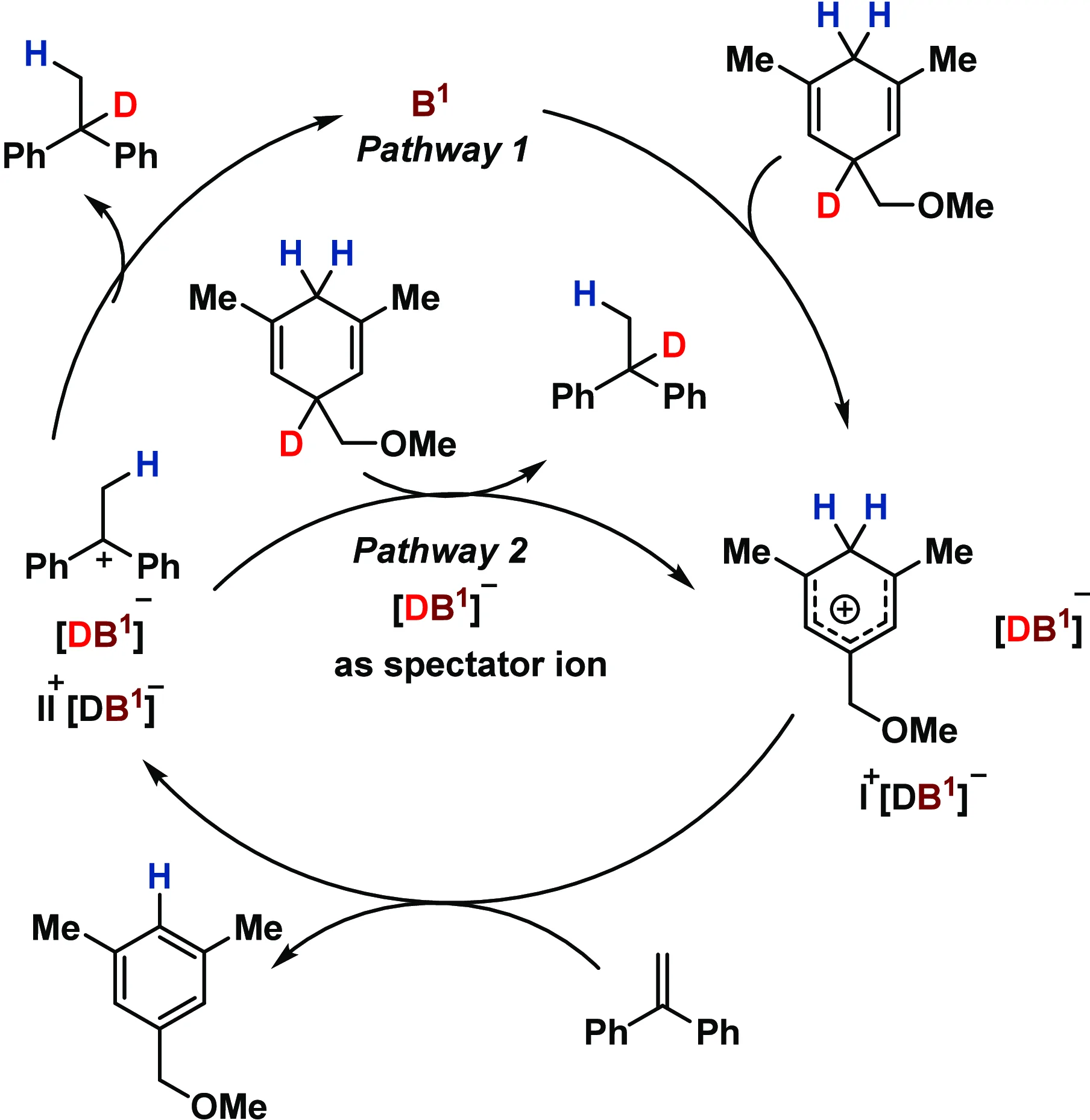
Reaction Mechanism: **B** ^**1**^-Catalyzed Regioselective Hydrodeuteration of Alkenes

Later, in 2020, Li and Hilt reported a regiodivergent protocol for the selective addition of hydrogen/deuterium (H/D) across 1,1-diarylalkenes, employing selectively deuterated dihydroaromatic compounds as transfer reagents. By fine-tuning the reaction conditions, the system enabled either deuterohydrogenation (D–H addition) or hydrodeuterogenation (H–D addition), affording complementary regioisomeric products with high selectivity.^260^ Mechanistic investigations indicated that the observed regioselectivity originated from controlled hydride/deuteride transfer pathways. Notably, the authors demonstrated that the less Lewis acidic BF_3_·Et_2_O can be used in place of **B** ^**1**^, offering comparable efficiency while providing advantages in terms of cost, operational simplicity, and the avoidance of glovebox techniques for regiodivergent HD/DH addition to alkenes.

## 8 Triarylborane-Catalyzed Transformations Using Hydroboranes

Pinacolborane (HBpin) has been known to act as a mild, air-stable, and chemoselective hydride donor, while the BAr_3_ catalyst facilitates activation either of the B–H bond or the substrate via Lewis acid–base interactions. In recent years, the combination of BAr_3_ and HBpin has emerged as a versatile platform for reductive transformations and hydrofunctionalization reactions, owing to the unique Lewis acidic activation of boranes and efficient hydride-transfer processes.^6^ From a mechanistic standpoint, BAr_3_ can promote σ-bond activation of HBpin or operate through FLP-type pathways, thereby enabling efficient hydride transfer processes. Such cooperative activation modes achieved the hydroboration of unsaturated functionalities (e.g., C=C, C=O, and C=N bonds) and reductive transformations.^261–270^ Overall, BAr_3_/HBpin catalytic systems represent a rapidly expanding area of main-group catalysis, offering transition-metal-free alternatives to conventional metal-catalyzed hydroboration and reduction methodologies, often with high efficiency, selectivity, and functional-group tolerance.

In 2020, Chang and co-workers reported a **B** ^**1**^-catalyzed double hydroboration of quinolines, enabling regioselective installation of an sp^3^ C–B bond at C3. The resulting 1,3-diborylated tetrahydroquinoline intermediates were subsequently transformed, via one-pot oxidative conversion, into 3-hydroxytetrahydroquinolines with high diastereocontrol.^271^ In 2021, Zhang and co-workers reported a one-pot, **B** ^**1**^-catalyzed protocol that enabled simultaneous synthesis of C3-regioselective functionalization of indoles and complete reduction of quinolines, demonstrating high regioselectivity.^272^ In the same year, Wang and co-workers employed HBpin as the hydroborating agent in this cascade.^87^ Hydroboration of the pyridine ring generates N-boryl dihydropyridine intermediates, and isotopic labeling experiments supported B–H bond activation with stepwise hydride transfer, delivering 2,3-disubstituted piperidines with pronounced cis diastereoselectivity (Scheme 22). Recently, Zhang and co-workers disclosed a **B** ^**1**^-catalyzed reductive protocol that provides an efficient route to C3-stannylated tetrahydroquinolines via cooperative hydroboration/hydrostannation of quinolines. Utilizing boranes (catecholborane or HBpin) and hydrostannanes ^*n*^Bu_3_SnH as coreductants, the method exhibited a broad substrate scope.^274^

The study by Wu and Gao reported a **B** ^**1**^-catalyzed tandem protonation/deuteration–reduction strategy for the transformation of *in situ*-generated enamines in the presence of H_2_O (or D_2_O) and HBpin.^275^ In this protocol, secondary amines and aldehydes underwent condensation to form enamines, which were subsequently converted into structurally diverse tertiary amines. Notably, the method enabled β-selective deuteration with high regioselectivity and efficient deuterium incorporation when D_2_O is employed, providing a practical route to β-deuterated amines. Mechanistically, the strongly Lewis acidic borane **B** ^**1**^ coordinates to the enamine to form a polarized boron–enamine complex. This intermediate engages in an FLP-type activation of H_2_O or D_2_O, facilitating heterolytic O–H (or O–D) bond cleavage and delivering regioselective protonation/deuteration at the β position. Concurrently, interaction of **B** ^**1**^ with HBpin generates a borohydride species that mediates hydride transfer, affording the reduced amine product. Overall, this tandem process enabled site-selective hydrogen/deuterium incorporation with broad functional group tolerance (Scheme 67). In 2022, Li and co-workers combined DFT calculations with experimental studies to elucidate the mechanism of imine hydroboration with HBpin. In **B** ^**1**^-catalyzed systems, the classical Lewis acid activation pathway was excluded due to a high-energy transition state, favoring a boron-mediated hydride transfer mechanism instead. Catalyst- and solvent-free conditions were found to be kinetically unfavorable. Furthermore, BH_3_·SMe_2_ was identified as an effective alternative catalyst, likely operating via a hydroboration pathway involving borane activation and subsequent hydride transfer.^276^

**Scheme 67:**
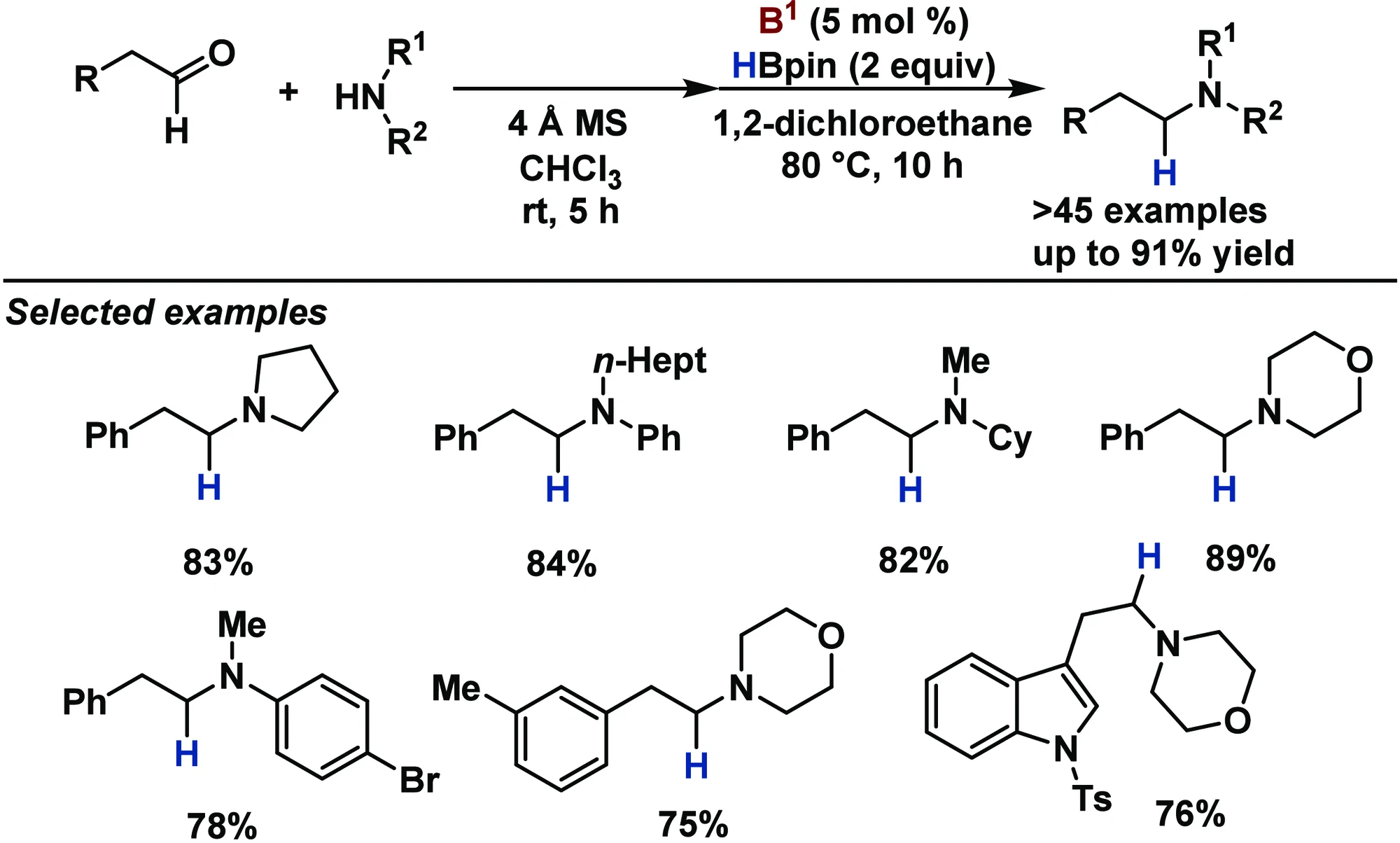
**B** ^**1**^-Catalyzed Reduction of *In Situ*-Formed Enamines Using HBpin

The study by Zhang and co-workers established a disproportionation-based pathway for **B** ^**1**^-catalyzed C–H borylation of indoles, enabling C3-selective formation of borylated indoles alongside transfer-hydrogenated indoline derivatives under ambient conditions.^277^ In 2023, Nad and Mukherjee reported a metal-free, operationally simple, and efficient catalytic protocol for the hydroboration of unprotected indoles to indolines.^278^ The reaction proceeded under mild conditions in THF using **B** ^**1**^ in combination with HBpin. This Lewis acid–base pair system facilitated heterolytic activation of the B–H bond of HBpin, generating a borohydride–borenium ion pair that enabled selective hydride delivery to the C2–C3 π system of indoles, effecting dearomatization to indoline derivatives with high chemo- and regioselectivity. Control experiments demonstrated the cooperative role of both components, as omission of either **B** ^**1**^ or HBpin suppresses reactivity. Isotopic labeling studies suggested that the hydride originates from the B–H bond. Mechanistic investigations further revealed the initial formation of a N–Bpin intermediate accompanied by H_2_ evolution (Scheme 68). The reduction of indoles and quinolines was reported by the Zhang group via a lanthanide/**B** ^**1**^-promoted hydroborative protocol employing HBpin. The transformation proceeds via the initial formation of indole–borane adducts, followed by sequential hydroboration mediated by HBpin and *in situ*-generated BH_3_ species, and concluded with protodeborylation to furnish the reduced heterocycles.^279^ The same group also reported that a LuCl_3_/**B** ^**1**^-cocatalyzed protocol enabled chemoselective deoxygenation of ketones, aldehydes, alcohols, and ethers using HBpin at room temperature. Mechanistic studies indicated that an *in situ*-generated cationic intermediate governed the reductive deoxygenation pathway.^280^

**Scheme 68:**
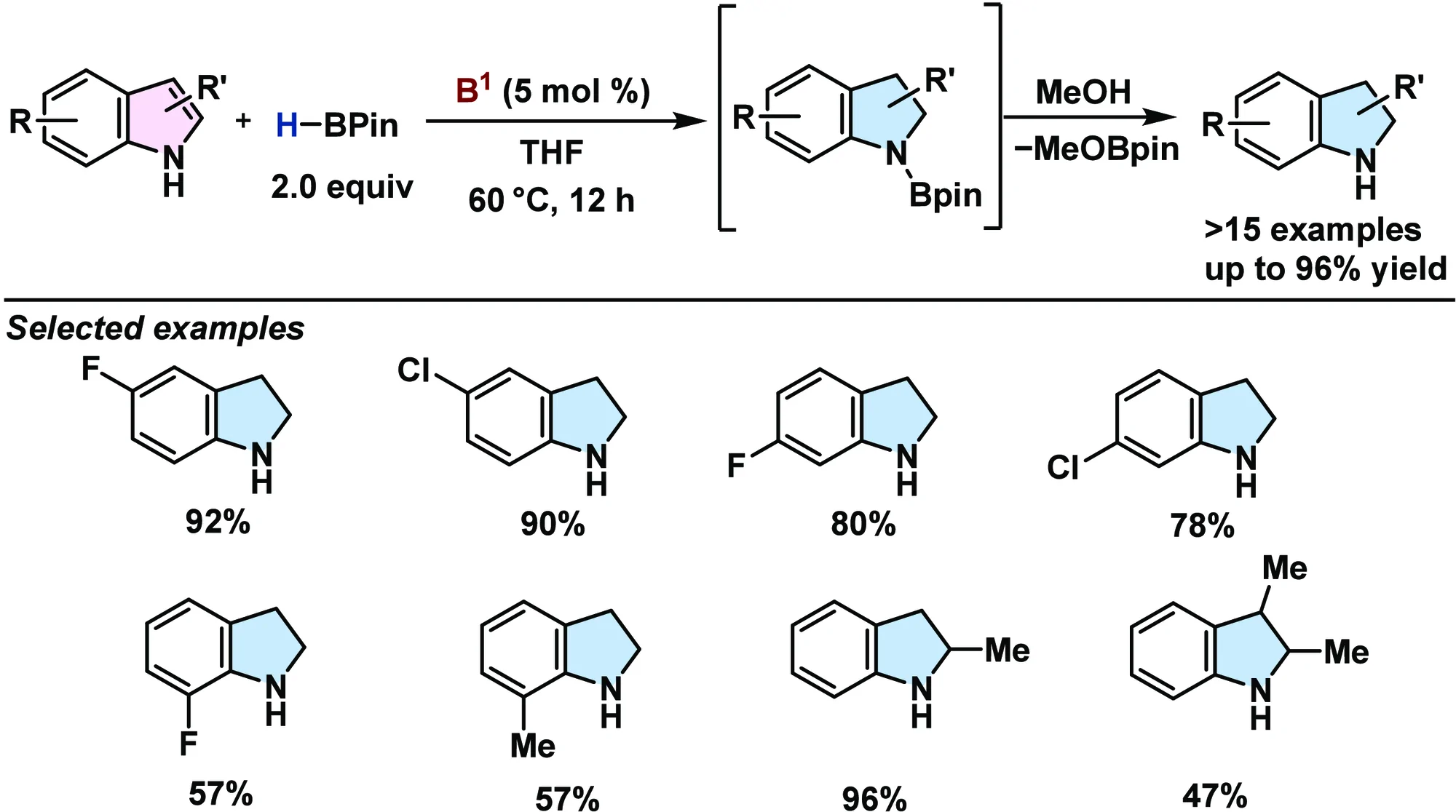
**B** ^**1**^-Catalyzed Hydrogenation of Unprotected Indoles Using HBpin

Recently, Zhang and co-workers reported an efficient **B** ^**1**^-catalyzed deoxygenative alkenylation of ketones for the synthesis of aryl alkenes, employing HBpin as a mild reducing agent.^281^ The protocol features operational simplicity and exhibits broad substrate scope, tolerating diverse functional groups including hydroxyl, amino, alkynyl, vinyl, and ester moieties. Notably, the method enabled late-stage functionalization of complex, pharmaceutically relevant molecules and exhibited excellent scalability, underscoring its strong potential for practical and large-scale synthetic applications. Mechanistic investigations revealed that the transformation proceeded via a concerted carbonyl hydroboration–deboration–deprotonation cascade. The catalytic cycle began with **B** ^**1**^-mediated activation of HBpin via a B···H interaction, facilitating efficient hydroboration of the carbonyl substrate. Subsequent Lewis acid coordination promoted the formation of a diboryl oxonium ion and a borohydride species. The key C–O bond cleavage generated a carbocationic intermediate, which was deprotonated to furnish the alkene product. Simultaneously, proton capture by the borohydride led to H_2_ evolution and regeneration of the active borane catalyst (Scheme 69). In 2025, Ong and co-workers investigated the reactivity of BAr_3_ with carbenes, and their application in the reduction of ketones using HBpin and PhSiH_3_.^282^ This study systematically explored FLP behavior of carbodicarbenes with boron Lewis acids, including **B** ^**1**^ and **B** ^**59**^. The systems spanned strongly bound adducts, reversibly dissociating pairs, and classical FLPs. A highly reactive, weakly bound adduct activated a broad range of bonds and catalyzed the hydroboration and hydrosilylation. NMR and DFT studies attributed reactivity to reversible dissociation and transient encounter complexes, enabling tunable main-group catalysis.

**Scheme 69:**
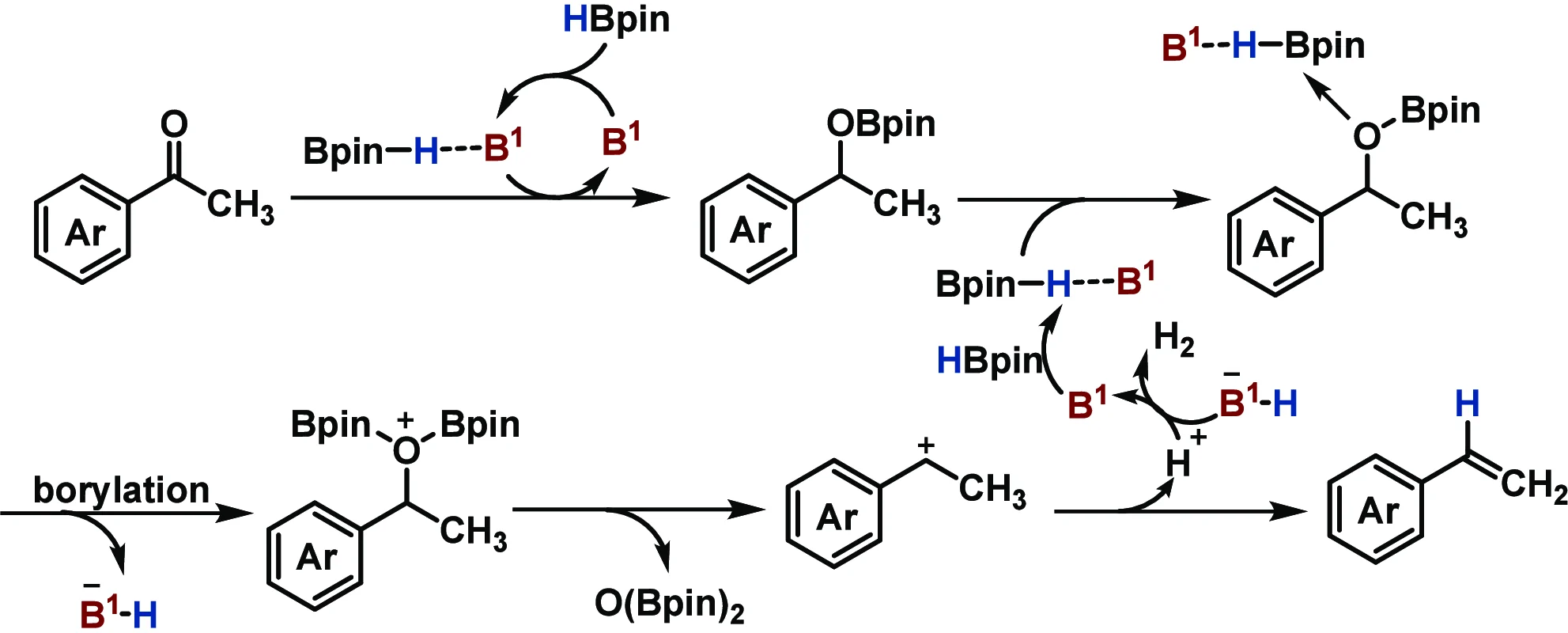
**B** ^**1**^-Catalyzed Deoxygenative Alkenylation of Ketones Using HBpin

## 9 Conclusions and Future Perspectives

Over the past decade, triarylborane catalysis has evolved into a versatile platform for metal-free reduction chemistry, undoubtedly expanding its utility in synthesizing diverse organic substrates by activating a broad range of reductants through a cooperative mechanism with Lewis-basic partners. Developments in strategies to design custom-tailored triarylboranes—particularly through electronic modulation, steric engineering, and heteroleptic architectures—have significantly expanded the catalytic scope and robustness of these systems. In particular, front-strain (i.e., intermolecular repulsion between boranes and Lewis bases) and proximal/remote back-strain (i.e., intramolecular repulsion in tetracoordinated Lewis base/borane adducts) manipulations have been recognized as critical strategies for finely tuning effective/global Lewis acidity and catalytic performance. Importantly, such strain-modulation strategies may work independently of the classical strategy for modulating intrinsic electronic parameters (e.g., the energy levels of the empty p orbitals on boron atoms). At the same time, it should be kept in mind that these boranes largely rely on polyfluorinated arenes, as their environmental persistence, and hence the associated safety risks, have not yet been fully evaluated or characterized.

Advances in custom-tailored triarylboranes have indeed enabled hitherto challenging transformations, including the hydrogenation of unsaturated molecules with crude H_2_ (a gaseous mixture of several components, including, but not limited to H_2_, CO, CO_2_, and CH_4_), and the selective hydrosilylation reactions of unsaturated and saturated bonds with high functional-group tolerance and operational simplicity. These systems demonstrate that rational molecular design can overcome catalyst deactivation and enable main-group catalysts to operate under industrially relevant conditions. This new direction, using industrially relevant, unpurified materials, offers an obvious benefit for applying main-group elements beyond the well-developed and reliable transition-metal-based systems established through extensive material purification. In hydrosilylation, moreover, transition-metal catalysts often require relatively reactive alkoxy- or halosilanes, or primary and secondary silanes, whereas triarylborane catalysis readily employs tertiary silanes through a mechanism that is largely independent of the hydrosilane used, a practical advantage in academic laboratories. These complementary features, rather than the metal-free aspect alone, define the distinctive value of triarylborane catalysis.

The applicable reductants in borane-catalyzed reduction reactions have also been largely diversified beyond conventional H_2_ and hydrosilanes. The use of Hantzsch esters, ammonia borane, and hydroboranes has provided complementary mechanistic pathways and improved compatibility with reactive polarized functional groups. Exploration of novel hydrogen donors, including stannanes and other emerging hydride sources, is expected to further broaden the scope of this chemistry.^283,284^

Despite these remarkable advances, several challenges still remain. A balanced comparison with transition-metal catalysis is nonetheless warranted. For benchmark reductions such as the reductive amination of carbonyl compounds,^285^ chiral transition-metal complexes still set the standard for enantioselectivity and often operate under milder conditions (for instance, the best earth-abundant transition-metal catalysts proceed at room temperature under atmospheric H_2_ pressure), whereas most of the triarylborane systems still require elevated H_2_ pressures and temperatures; enantioselective variants remain limited. Achieving higher turnover numbers (TONs) under mild conditions, improving catalyst stability toward protodeboronation and strong Lewis bases, and expanding substrate classes to more complex and multifunctional molecules also remain active areas of investigation. Additionally, inspiration from state-of-the-art techniques widely applied in transition-metal-catalyzed systems, such as photo- and electro-driven excited-state chemistry, mechanochemistry, and related approaches, may open new opportunities for tailored triarylborane catalysts by enabling alternative reaction pathways and expanding the scope of reductive transformations under mild and sustainable conditions. Looking ahead, the integration of computational design, data-driven catalyst optimization, and mechanistic analysis is expected to accelerate the discovery of next-generation tailored triarylboranes with bespoke reactivity and improved robustness. The continued exploration of unconventional reductants, cooperative catalytic systems, and impurity-tolerant hydrogen activation will further broaden the applicability of borane catalysis. Ultimately, these developments position triarylboranes as key components of sustainable main-group catalysis, complementing—rather than simply replacing—both precious- and earth-abundant-metal systems, while enabling efficient reductive transformations.

## References

[1] Revunova K., Nikonov G. I. (2015). Main Group Catalysed
Reduction of Unsaturated Bonds. Dalton Trans..

[2] Wilkins L. C., Melen R. L. (2016). Enantioselective
Main Group Catalysis: Modern Catalysts
for Organic Transformations. Coord. Chem. Rev..

[3] Lam J., Szkop K. M., Mosaferi E., Stephan D. W. (2019). FLP Catalysis: Main
Group Hydrogenations of Organic Unsaturated Substrates. Chem. Soc. Rev..

[4] Sivaev I. B., Bregadze V. I. (2014). Lewis Acidity of Boron Compounds. Coord. Chem. Rev..

[5] Oestreich M., Hermeke J., Mohr J. (2015). A Unified Survey of
Si-H and H-H
Bond Activation Catalyzed by Electron-Deficient Boranes. Chem. Soc. Rev..

[6] Lawson J. R., Melen R. L. (2017). Tris­(pentafluorophenyl)­borane and Beyond: Modern Advances
in Borylation Chemistry. Inorg. Chem..

[7] Hoshimoto Y., Ogoshi S. (2019). Triarylborane-Catalyzed
Reductive N-Alkylation of Amines:
A Perspective. ACS Catal..

[8] Paradies J. (2023). Structure-Reactivity
Relationships in Borane-Based FLP-Catalyzed Hydrogenations, Dehydrogenations,
and Cycloisomerizations. Acc. Chem. Res..

[9] Carden J. L., Dasgupta A., Melen R. L. (2020). Halogenated
Triarylboranes: Synthesis,
Properties and Applications in Catalysis. Chem.
Soc. Rev..

[10] Ferger M., Berger S. M., Rauch F., Schönitz M., Rühe J., Krebs J., Friedrich A., Marder T. B. (2021). Synthesis of Highly Functionalizable Symmetrically
and Unsymmetrically Substituted Triarylboranes from Bench-Stable Boron
Precursors. Chem.—Eur. J..

[11] Berger S. M., Marder T. B. (2022). Applications of
Triarylborane Materials in Cell Imaging
and Sensing of Bio-relevant Molecules such as DNA, RNA, and Proteins. Mater. Horiz..

[12] Nori V., Pesciaioli F., Sinibaldi A., Giorgianni G., Carlone A. (2022). Boron-Based Lewis Acid Catalysis: Challenges and Perspectives. Catalysts.

[13] Sakuraba M., Hoshimoto Y. (2024). Recent Trends
in Triarylborane Chemistry: Diversification
of Structures and Reactivity via meta-Substitution of the Aryl Groups. Synthesis.

[14] Morishita T., Hisata Y., Hashimoto T., Ogoshi S., Hoshimoto Y. (2024). Triarylborane
Catalysis: From Hydrogenation of Unsaturated Molecules to H_2_ Purification. J. Synth. Org. Chem., Jpn..

[15] Jupp A. R., Stephan D. W. (2019). New Directions for
Frustrated Lewis Pair Chemistry. Trends Chem..

[16] Stephan D. W. (2015). Frustrated
Lewis Pairs: From Concept to Catalysis. Acc.
Chem. Res..

[17] Stephan D.
W., Erker G. (2015). Frustrated
Lewis Pair Chemistry: Development and Perspectives. Angew. Chem., Int. Ed..

[18] Scott D. J., Fuchter M. J., Ashley A. E. (2017). Designing Effective “Frustrated
Lewis Pair” Hydrogenation Catalysts. Chem. Soc. Rev..

[19] Fasano V., Ingleson M. J. (2018). Recent Advances in Water-Tolerance in Frustrated Lewis
Pair Chemistry. Synthesis.

[20] Stephan D. W. (2021). Diverse
Uses of the Reaction of Frustrated Lewis Pair (FLP) with Hydrogen. J. Am. Chem. Soc..

[21] Zhou R., Tavandashti Z. P., Paradies J. (2023). Frustrated Lewis Pair Catalyzed Reactions. SynOpen.

[22] Stephan D. W. (2016). The Broadening
Reach of Frustrated Lewis Pair Chemistry. Science.

[23] Wech F., Gellrich U. (2022). Hydrogenation of Olefins,
Alkynes, Allenes, and Arenes
by Borane-Based Frustrated Lewis Pairs. Synthesis.

[24] Wilkins L. C., Howard J. L., Burger S., Frentzel-Beyme L., Browne D. L., Melen R. L. (2017). Exploring Multistep
Continuous-Flow
Hydrosilylation Reactions Catalyzed by Tris­(pentafluorophenyl)­borane. Adv. Synth. Catal..

[25] Liu L., Lukose B., Jaque P., Ensing B. (2019). Reaction Mechanism
of Hydrogen Activation by Frustrated Lewis Pairs. Green Energy Environ..

[26] Guerzoni M. G., Dasgupta A., Richards E., Melen R. L. (2022). Enantioselective
Applications of Frustrated Lewis Pairs in Organic Synthesis. Chem. Catal..

[27] Feng X., Meng W., Du H. (2023). Asymmetric Catalysis
with FLPs. Chem. Soc. Rev..

[28] Zhan Z., Yan J., Yu Z., Shi L. (2023). Recent Advances in Asymmetric Catalysis
Associated with B­(C_6_F_5_)_3_. Molecules.

[29] Pramanik M., Guerzoni M. G., Richards E., Melen R. L. (2024). Recent Advances
in Asymmetric Catalysis Using *p*-Block Elements. Angew. Chem., Int. Ed..

[30] Meng W., Feng X., Du H. (2018). Frustrated Lewis Pairs Catalyzed
Asymmetric Metal-Free Hydrogenations and Hydrosilylations. Acc. Chem. Res..

[31] Massey A. G., Park A. J., Stone F. G. A. (1963). Tris­(pentafluorophenyl)­borane. Proc. Chem. Soc..

[32] Massey A. G., Park A. J. (1964). Perfluorophenyl
Derivatives of the Elements: I. Tris­(pentafluorophenyl)­boron. J. Organomet. Chem..

[33] Ikeda, Y. ; Yamane, T. ; Kaji, E. ; Ishimaru, K. Method of Producing Pentafluorophenyl Alkali Metal Salt Using Pentafluorobenzene in an Open Chain Ether Type Solvent. EP 0604960, 1993.

[34] Berger S. M., Ferger M., Marder T. B. (2021). Synthetic Approaches to Triarylboranes
from 1885 to 2020. Chem.—Eur. J..

[35] Wang Z., Buser A. M., Cousins I. T., Demattio S., Drost W., Johansson O., Ohno K., Patlewicz G., Richard A. M., Walker G. W. (2021). A New OECD Definition
for Per- and Polyfluoroalkyl Substances. Environ.
Sci. Technol..

[36] Tiedt O., Mergelsberg M., Boll K., Müller M., Adrian L., Jehmlich N., von Bergen M., Boll M. (2016). ATP-Dependent C-F Bond Cleavage Allows the Complete Degradation of
4-Fluoroaromatics without Oxygen. mBio.

[37] Blagg R. J., Lawrence E. J., Resner K., Oganesyan V. S., Herrington T. J., Ashley A. E., Wildgoose G. G. (2016). Exploring
Structural and Electronic Effects in Three Isomers of Tris­{bis­(trifluoromethyl)­phenyl}­borane:
Towards the Combined Electrochemical-Frustrated Lewis Pair Activation
of H_2_. Dalton Trans..

[38] Yin Q., Soltani Y., Melen R. L., Oestreich M. (2017). BAr^F^
_3_-Catalyzed Imine Hydroboration
with Pinacolborane Not
Requiring the Assistance of an Additional Lewis Base. Organometallics.

[39] Lin S., Miller J. M. (1977). Decomposition and Byproducts from Reactions Involving
Pentafluorophenyl-Grignard and Lithium Reagents. A GC/MS Study. J. Fluorine Chem..

[40] Sun Y., Piers W. E., Parvez M. (1998). The Solid-State Structure of Bis­(pentafluorophenyl)­zinc. Can. J. Chem..

[41] Adonin N. Yu., Bardin V. V. (2010). Polyfluorinated Organic Compounds of Boron. Russ. Chem. Rev..

[42] Köring L., Sitte N. A., Bursch M., Grimme S., Paradies J. (2021). Hydrogenation
of Secondary Amides Using Phosphane Oxide and Frustrated Lewis Pair
Catalysis. Chem.—Eur. J..

[43] Korte L. A., Schwabedissen J., Soffner M., Blomeyer S., Reuter C. G., Vishnevskiy Y. V., Neumann B., Stammler H.-G., Mitzel N. W. (2017). Tris­(perfluorotolyl)­borane—A
Boron Lewis Superacid. Angew. Chem., Int. Ed..

[44] Chase P. A., Henderson L. D., Piers W. E., Parvez M., Clegg W., Elsegood M. R. (2006). Bifunctional
Perfluoroaryl Boranes: Synthesis and Coordination
Chemistry with Neutral Lewis Base Donors. Organometallics.

[45] Mohr J., Durmaz M., Irran E., Oestreich M. (2014). Tris­(5,6,7,8-Tetrafluoronaphthalen-2-yl)­borane,
a Partially Fluorinated Boron Lewis Acid with Fluorination Distal
to the Boron Atom. Organometallics.

[46] Kovács B., Földes T., Szabó M., Dorkó É., Kótai B., Laczkó G., Holczbauer T., Domján A., Pápai I., Soós T. (2024). Illuminating
the Multiple Lewis Acidity of Triarylboranes via Atropisomeric Dative
Adducts. Chem. Sci..

[47] Sakuraba M., Morishita T., Hashimoto T., Ogoshi S., Hoshimoto Y. (2023). Remote Back
Strain: A Strategy for Modulating the Reactivity of Triarylboranes. Synlett.

[48] Ben
Saida A., Chardon A., Osi A., Tumanov N., Wouters J., Adjieufack A. I., Champagne B., Berionni G. (2019). Pushing the Lewis Acidity Boundaries of Boron Compounds
with Non-Planar Triarylboranes Derived from Triptycenes. Angew. Chem., Int. Ed..

[49] Chardon A., Osi A., Mahaut D., Ben Saida A., Berionni G. (2020). Non-Planar Boron Lewis
Acids Taking the Next Step: Development of Tunable Lewis Acids, Lewis
Superacids and Bifunctional Catalysts. Synlett.

[50] Chardon A., Osi A., Mahaut D., Doan T.-H., Tumanov N., Wouters J., Fusaro L., Champagne B., Berionni G. (2020). Controlled Generation
of 9-Boratriptycene by Lewis Adduct Dissociation: Accessing a Non-Planar
Triarylborane. Angew. Chem., Int. Ed..

[51] Osi A., Mahaut D., Tumanov N., Fusaro L., Wouters J., Champagne B., Chardon A., Berionni G. (2022). Taming the Lewis Superacidity
of Non-Planar Boranes: C-H Bond Activation and Non-Classical Binding
Modes at Boron. Angew. Chem., Int. Ed..

[52] Dorkó É., Szabó M., Kótai B., Pápai I., Domján A., Soós T. (2017). Expanding the Boundaries of Water-Tolerant
Frustrated Lewis Pair Hydrogenation: Enhanced Back Strain in the Lewis
Acid Enables the Reductive Amination of Carbonyls. Angew. Chem., Int. Ed..

[53] Sakuraba M., Ogoshi S., Hoshimoto Y. (2024). Strategic Use of Crude H_2_ for the Catalytic Reduction of Carbonyl Compounds. Tetrahedron Chem..

[54] Heshmat M., Ensing B. (2020). Optimizing the Energetics of FLP-Type H2 Activation
by Modulating the Electronic and Structural Properties of the Lewis
Acids: A DFT Study. J. Phys. Chem. A.

[55] Hirose K., Maeda S., Matsuoka W. (2025). Conceptual
Expansion of Virtual Ligand
Strategy Toward the Design of Triarylborane Catalysts. J. Comput. Chem..

[56] Blagg R. J., Simmons T. R., Hatton G. R., Courtney J. M., Bennett E. L., Lawrence E. J., Wildgoose G. G. (2016). Novel B­(Ar′)_2_(Ar″)
Hetero-tri­(aryl)­boranes: A Systematic Study of Lewis Acidity. Dalton Trans..

[57] Blagg R. J., Wildgoose G. G. (2016). H_2_ Activation Using the First 1:1:1 Hetero-tri­(aryl)­borane. RSC Adv..

[58] Dorkó É., Kótai B., Földes T., Gyömöre Á., Pápai I., Soós T. (2017). Correlating Electronic and Catalytic
Properties of Frustrated Lewis Pairs for Imine Hydrogenation. J. Organomet. Chem..

[59] Bakos M., Dobi Z., Fegyverneki D., Gyömöre Á., Fernández I., Soós T. (2018). Janus Face
of the Steric Effect in a Lewis Acid Catalyst with Size-Exclusion
Design: Steric Repulsion and Steric Attraction in the Catalytic Exo-Selective
Diels-Alder Reaction. ACS Sustainable Chem.
Eng..

[60] Hashimoto T., Asada T., Ogoshi S., Hoshimoto Y. (2022). Main Group
Catalysis for H_2_ Purification Based on Liquid Organic Hydrogen
Carriers. Sci. Adv..

[61] Hisata Y., Washio T., Takizawa S., Ogoshi S., Hoshimoto Y. (2024). *In-Silico*-Assisted Derivatization of Triarylboranes for the Catalytic Reductive
Functionalization of Aniline-Derived Amino Acids and Peptides with
H_2_. Nat. Commun..

[62] Dudás A., Gyömöre Á., Mészáros B. B., Gondár S., Adamik R., Fegyverneki D., Papp D., Otte K. B., Ayala S., Daru J., Repási J., Soós T. (2025). Selective
Reduction of Esters to Access Aldehydes Using Fiddler Crab-Type Boranes. J. Am. Chem. Soc..

[63] Hisata Y., Ikeda R., Chauhan A., Hoshimoto Y. (2026). Triarylboranes
Bearing (*ortho*-Carboran-1-yl)­aryl Groups for Catalytic
Hydrogenation with Pure or Crude H_2_. Org. Lett..

[64] Hisata Y., Morishita D., Hoshimoto Y. (2025). An Isolated Lithium ortho-Carboranyl
Cuprate Complex for the Synthesis of Multiple-Carborane-Substituted
Arenes from (Hetero)­Aryl Bromides and Chlorides. J. Am. Chem. Soc..

[65] Qin Y., Cheng G., Sundararaman A., Jäkle F. (2002). Well-Defined
Boron-Containing Polymeric Lewis Acids. J. Am.
Chem. Soc..

[66] Wang M., Nudelman F., Matthes R. R., Shaver M. P. (2017). Frustrated Lewis
Pair Polymers as Responsive Self-Healing Gels. J. Am. Chem. Soc..

[67] Yolsal U., Wang M., Royer J. R., Shaver M. P. (2019). Rheological Characterization
of Polymeric Frustrated Lewis Pair Networks. Macromolecules.

[68] Chen L., Liu R., Yan Q. (2018). Polymer Meets Frustrated
Lewis Pair: Second-Generation
CO_2_-Responsive Nanosystem for Sustainable CO_2_ Conversion. Angew. Chem., Int. Ed..

[69] Lin H., Patel S., Jäkle F. (2020). Tailored Triarylborane
Polymers as
Supported Catalysts and Luminescent Materials. Macromolecules.

[70] Vidal F., McQuade J., Lalancette R., Jäkle F. (2020). ROMP-Boranes
as Moisture-Tolerant and Recyclable Lewis Acid Organocatalysts. J. Am. Chem. Soc..

[71] Welch G. C., San Juan R. R., Masuda J. D., Stephan D. W. (2006). Reversible, Metal-Free
Hydrogen Activation. Science.

[72] Chase P. A., Welch G. C., Jurca T., Stephan D. W. (2007). Metal-Free Catalytic
Hydrogenation. Angew. Chem., Int. Ed..

[73] Welch G. C., Stephan D. W. (2007). Facile Heterolytic
Cleavage of Dihydrogen by Phosphines
and Boranes. J. Am. Chem. Soc..

[74] Spies P., Erker G., Kehr G., Bergander K., Fröhlich R., Grimme S., Stephan D. W. (2007). Rapid Intramolecular
Heterolytic Dihydrogen Activation by a Four-Membered Heterocyclic
Phosphane-Borane Adduct. Chem. Commun..

[75] Erős G., Mehdi H., Pápai I., Rokob T. A., Király P., Tárkányi G., Soós T. (2010). Expanding
the Scope of Metal-Free Catalytic Hydrogenation through Frustrated
Lewis Pair Design. Angew. Chem., Int. Ed..

[76] Paradies J. (2014). Metal-Free
Hydrogenation of Unsaturated Hydrocarbons Employing Molecular Hydrogen. Angew. Chem., Int. Ed..

[77] Hounjet L. J., Stephan D. W. (2014). Hydrogenation by frustrated Lewis pairs: main group
alternatives to transition metal catalysts?. Org. Process Res. Dev..

[78] Mahdi T., Stephan D. W. (2015). Facile Protocol for Catalytic Frustrated Lewis Pair
Hydrogenation and Reductive Deoxygenation of Ketones and Aldehydes. Angew. Chem., Int. Ed..

[79] Scott D. J., Simmons T. R., Lawrence E. J., Wildgoose G. G., Fuchter M. J., Ashley A. E. (2015). Facile Protocol for Water-Tolerant
“Frustrated Lewis Pair”-Catalyzed Hydrogenation. ACS Catal..

[80] Gyömöre A., Bakos M., Földes T., Pápai I., Domján A., Soós T. (2015). Moisture-Tolerant
Frustrated Lewis
Pair Catalyst for Hydrogenation of Aldehydes and Ketones. ACS Catal..

[81] Bakos M., Gyömöre Á., Domján A., Soós T. (2017). Auto-Tandem Catalysis with Frustrated Lewis Pairs for
Reductive Etherification of Aldehydes and Ketones. Angew. Chem., Int. Ed..

[82] Hoshimoto Y., Kinoshita T., Hazra S., Ohashi M., Ogoshi S. (2018). Main-Group-Catalyzed
Reductive Alkylation of Multiply Substituted Amines with Aldehydes
Using H_2_. J. Am. Chem. Soc..

[83] Khan I., Manzotti M., Tizzard G. J., Coles S. J., Melen R. L., Morrill L. C. (2017). Frustrated Lewis
Pair (FLP)-Catalyzed Hydrogenation
of Aza-Morita-Baylis-Hillman Adducts and Sequential Organo-FLP Catalysis. ACS Catal..

[84] Tussing S., Greb L., Tamke S., Schirmer B., Muhle-Goll C., Luy B., Paradies J. (2015). Autoinduced Catalysis
and Inverse Equilibrium Isotope
Effect in the Frustrated Lewis Pair Catalyzed Hydrogenation of Imines. Chem.—Eur. J..

[85] Tussing S., Kaupmees K., Paradies J. (2016). Structure-Reactivity Relationship
in the Frustrated Lewis Pair (FLP)-Catalyzed Hydrogenation of Imines. Chem.—Eur. J..

[86] Sitte N. A., Bursch M., Grimme S., Paradies J. (2019). Frustrated Lewis Pair
Catalyzed Hydrogenation of Amides: Halides as Active Lewis Base in
the Metal-Free Hydrogen Activation. J. Am. Chem.
Soc..

[87] Yang Z. Y., Luo H., Zhang M., Wang X. C. (2021). Borane-Catalyzed Reduction of Pyridines
via a Hydroboration/Hydrogenation Cascade. ACS
Catal..

[88] Jian Z., Krupski S., Škoch K., Kehr G., Daniliuc C. G., Císařová I., Štěpnička P., Erker G. (2017). Phosphine-Borane Frustrated Lewis Pairs Derived from a 1,1′-Disubstituted
Ferrocene Scaffold: Synthesis and Hydrogenation Catalysis. Organometallics.

[89] Thomson J., Courtemanche M. A., Neu R. C., Stephan D. W. (2025). FLP-CO_2_ Adducts: Convenient Sources of Frustrated Lewis Pairs for
Stoichiometric
and Catalytic Chemistry. Dalton Trans..

[90] Houghton A. Y., Autrey T. (2017). Calorimetric Study
of the Activation of Hydrogen by
Tris­(pentafluorophenyl)­borane and Trimesitylphosphine. J. Phys. Chem. A.

[91] Hashimoto T., Tanigawa M., Kambe K., Ogoshi S., Hoshimoto Y. (2025). Boosting Turnover
in the Triarylborane-Catalyzed Hydrogenation of N-Substituted Indoles
via Olefin-to-Nitrogen Lewis Base Switching in H_2_-Cleavage
Steps. Precis. Chem..

[92] Wang W., Meng W., Du H. (2016). B­(C_6_F_5_)_3_-Catalyzed Metal-Free Hydrogenation of
3,6-Diarylpyridazines. Dalton Trans..

[93] Han C., Zhang E., Feng X., Wang S., Du H. (2018). B­(C_6_F_5_)_3_-Catalyzed Metal-Free Hydrogenations of
2-Quinolinecarboxylates. Tetrahedron Lett..

[94] Liu X., Liu T., Meng W., Du H. (2018). Highly Stereoselective Metal-Free
Hydrogenations of Pyrrolo­[1,2-*a*]­quinoxalines. Org. Lett..

[95] Dai Y., Feng X., Du H. (2019). B­(C_6_F_5_)_3_-Catalyzed Highly Stereoselective Hydrogenation of Unfunctionalized
Tetrasubstituted Olefins. Org. Lett..

[96] Gao B., Feng X., Meng W., Du H. (2020). Asymmetric Hydrogenation
of Ketones and Enones with Chiral Lewis Base Derived Frustrated Lewis
Pairs. Angew. Chem..

[97] Dai Y., Meng W., Feng X., Du H. (2022). Chiral FLP-Catalyzed
Asymmetric Hydrogenation of 3-Fluorinated Chromones. Chem. Commun..

[98] Han Z., Feng X., Du H. (2023). Asymmetric Hydrogenations of Acyclic
α,β-Unsaturated Ketones with Chiral Frustrated Lewis Pairs
(FLPs). J. Org. Chem..

[99] Guo J., Jin L., Xiao L., Yan M., Xiong J., Qu Z.-W., Grimme S., Stephan D. W. (2024). Readily
Accessible Chiral Frustrated
Lewis Pair Catalysts: Asymmetric Hydrosilylation of Imines and Quinolines. Chem. Catal..

[100] Chen J., Gao B., Feng X., Meng W., Du H. (2021). Relay Catalysis by
Achiral Borane and Chiral Phosphoric Acid in the
Metal-Free Asymmetric Hydrogenation of Chromones. Org. Lett..

[101] Daru J., Bakó I., Stirling A., Pápai I. (2019). Mechanism
of Heterolytic Hydrogen Splitting by Frustrated Lewis Pairs: Comparison
of Static and Dynamic Models. ACS Catal..

[102] Hamza A., Sorochkina K., Kótai B., Chernichenko K., Berta D., Bolte M., Nieger M., Repo T., Pápai I. (2020). Origin of
Stereoselectivity in FLP-Catalyzed
Asymmetric Hydrogenation of Imines. ACS Catal..

[103] Kótai B., Laczkó G., Hamza A., Pápai I. (2024). Stereocontrol
via Propeller Chirality in FLP-Catalyzed Asymmetric Hydrogenation. Chem.—Eur. J..

[104] Zhao T., Hu X., Wu Y., Zhang Z. (2019). Hydrogenation
of CO_2_ to Formate with H_2_: Transition Metal-Free
Catalyst Based on a Lewis Pair. Angew. Chem..

[105] Durin G., Fontaine A., Berthet J. C., Nicolas E., Thuéry P., Cantat T. (2022). Metal-Free Catalytic
Hydrogenolysis
of Silyl Triflates and Halides into Hydrosilanes. Angew. Chem., Int. Ed..

[106] Shcherbakov N. V., Potin N., Mas-Roselló J. (2026). Diastereoselective
Synthesis of *cis*-α,α′-Disubstituted
Cyclic Ethers via Borane-Catalyzed Reductive Cyclization of Diketones. ACS Catal..

[107] Hoshimoto, Y. ; Ogoshi, S. ; Tanaka, T. ; Kawamoto, N. Method for Hydrogenating Unsaturated Compound. Patent JP2017206474A, 2017; https://patents.google.com/patent/JP2017206474A/en.

[108] Wang, T. ; Daniliuc, C. G. ; Kehr, G. ; Erker, G. FLP Reduction of Carbon Monoxide and Related Reactions. In Frustrated Lewis Pairs; Slootweg, J. C. , Jupp, A. R. , Eds.; Molecular Catalysis, Vol. 2; Springer: Cham, Switzerland, 2021; pp 87–112.

[109] Stephan D. W. (2023). Frustrated
Lewis Pair Chemistry of CO. Chem. Soc. Rev..

[110] Hashimoto T., Harabuchi Y., Ogoshi S., Maeda S., Hoshimoto Y. (2025). Elucidating
Multicomponent Mechanisms in the Catalytic
Hydrogenation of 2-Methylquinoline under Crude-H_2_ Conditions:
A Key H_2_-Cleavage Process by a Boron-Olefin Lewis Pair. Bull. Chem. Soc. Jpn..

[111] Piers W. E., Chivers T. (1997). Pentafluorophenylboranes: From Obscurity
to Applications. Chem. Soc. Rev..

[112] Parks D. J., Piers W. E. (1996). Tris­(pentafluorophenyl)­boron-Catalyzed
Hydrosilation of Aromatic Aldehydes, Ketones, and Esters. J. Am. Chem. Soc..

[113] Parks D. J., Blackwell J. M., Piers W. E. (2000). Studies on the Mechanism
of B­(C_6_F_5_)_3_-Catalyzed Hydrosilation
of Carbonyl Functions. J. Org. Chem..

[114] Houghton A. Y., Hurmalainen J., Mansikkamaki A., Piers W. E., Tuononen H. M. (2014). Direct Observation
of a Borane-Silane
Complex Involved in Frustrated Lewis-Pair-Mediated Hydrosilylations. Nat. Chem..

[115] Rendler S., Oestreich M. (2008). Conclusive Evidence for an S_N2_-Si Mechanism
in the B­(C_6_F_5_)_3_-Catalyzed Hydrosilylation
of Carbonyl Compounds: Implications for
the Related Hydrogenation. Angew. Chem., Int.
Ed..

[116] Zhang Q., Fu M. C., Yu H. Z., Fu Y. (2016). Mechanism
of Boron-Catalyzed N-Alkylation of Amines with Carboxylic Acids. J. Org. Chem..

[117] Del Rio N., Lopez-Reyes M., Baceiredo A., Saffon-Merceron N., Lutters D., Müller T., Kato T. (2017). N, P-Heterocyclic Germylene/B­(C_6_F_5_)_3_ Adducts: A Lewis Pair with Multi-Reactive Sites. Angew. Chem., Int. Ed..

[118] Du P., Zhao J. (2018). Density Functional Theory Mechanistic Study of Boron-Catalyzed
N-Alkylation of Amines with Formic Acid: Formic Acid Activation by
Silylation Reaction. Chem.—Asian J..

[119] Rao B., Wang L., Kinjo R. (2019). Borane-Catalyzed
Cross-Metathesis
Strategy for Facile Transformation of Cyclic (Alkyl)­(Amino) Germylenes. Angew. Chem..

[120] Zhou M., Wang T., Cheng G. J. (2022). Mechanistic Insights
into Reductive Deamination with Hydrosilanes Catalyzed by B­(C_6_F_5_)_3_: A DFT Study. Front. Chem..

[121] Guzmán J., Urriolabeitia A., Padilla M., García-Orduña P., Polo V., Fernández-Alvarez F. J. (2022). Mechanism Insights
into the Iridium (III)- and B­(C_6_F_5_)_3_-Catalyzed Reduction of CO_2_ to the Formaldehyde Level
with Tertiary Silanes. Inorg. Chem..

[122] He Y., Wen Z., Nie W., Yang L. (2024). Mechanistic Study of
B­(C_6_F_5_)_3_-Catalyzed Transfer Hydrogenation
of Aldehydes/Ketones with PhSiH_3_ and Stoichiometric Water. ACS Omega.

[123] Hackel T., McGrath N. A. (2019). Tris­(pentafluorophenyl)­borane-Catalyzed
Reactions Using Silanes. Molecules.

[124] Park S. (2019). B­(C_6_F_5_)_3_-Catalyzed sp^3^ C-Si Bond Forming Consecutive Reactions. Chin.
J. Chem..

[125] Fang H., Oestreich M. (2020). Defunctionalisation Catalysed by
Boron Lewis Acids. Chem. Sci..

[126] Sawamura M., Shimizu Y. (2023). Boron Catalysis in
the Transformation
of Carboxylic Acids and Carboxylic Acid Derivatives. Eur. J. Org. Chem..

[127] Moitra N., Ichii S., Kamei T., Kanamori K., Zhu Y., Takeda K., Nakanishi K., Shimada T. (2014). Surface Functionalization
of Silica by Si-H Activation of Hydrosilanes. J. Am. Chem. Soc..

[128] Lee P. T., Rosenberg L. (2017). Borane-Catalysed Postpolymerisation
Modification of the Si-H Bonds in Poly­(phenylsilane). Dalton Trans..

[129] Laengert S. E., Schneider A. F., Lovinger E., Chen Y., Brook M. A. (2017). Sequential
Functionalization of a Natural Crosslinker
Leads to Designer Silicone Networks. Chem.—Asian
J..

[130] Escorihuela J., Pujari S. P., Zuilhof H. (2017). Organic Monolayers
by B­(C_6_F_5_)_3_-Catalyzed Siloxanation
of Oxidized Silicon Surfaces. Langmuir.

[131] Matsumoto K., Sajna K. V., Satoh Y., Sato K., Shimada S. (2017). By-Product-Free Siloxane-Bond Formation
and Programmed
One-Pot Oligosiloxane Synthesis. Angew. Chem.,
Int. Ed..

[132] Zhang J., Chen Y., Sewell P., Brook M. A. (2015). Utilization
of Softwood Lignin as Both Crosslinker and Reinforcing Agent in Silicone
Elastomers. Green Chem..

[133] Matsumoto K., Oba Y., Nakajima Y., Shimada S., Sato K. (2018). One-Pot Sequence-Controlled Synthesis
of Oligosiloxanes. Angew. Chem..

[134] Hein N. M., Seo Y., Lee S. J., Gagné M. R. (2019). Harnessing
the Reactivity of Poly­(methylhydrosiloxane) for the Reduction and
Cyclization of Biomass to High-Value Products. Green Chem..

[135] Matsumoto K., Shimada S., Sato K. (2019). Sequence-Controlled
Catalytic One-Pot Synthesis of Siloxane Oligomers. Chem.—Eur. J..

[136] Ai L., Chen Y., He L., Luo Y., Li S., Xu C. (2019). Synthesis of Structured Polysiloxazanes via a Piers-Rubinsztajn Reaction. Chem. Commun..

[137] Kaźmierczak J., Lewandowski D., Hreczycho G. (2020). B­(C_6_F_5_)_3_-Catalyzed Dehydrocoupling of POSS Silanols
with Hydrosilanes: A Metal-Free Strategy for Effecting Functionalization
of Silsesquioxanes. Inorg. Chem..

[138] Kawatsu T., Choi J. C., Sato K., Matsumoto K. (2021). Facile Synthesis
of Sequence-Defined Oligo­(dimethylsiloxane-*co*-diphenylsiloxane)­s. Macromol. Rapid Commun..

[139] Liu T., He J., Zhang Y. (2023). Application of HSAB Theory in the
Selective Reduction of Quinoline. Chin. J. Chem..

[140] Rubinsztajn S., Chojnowski J., Mizerska U. (2023). Tris­(pentafluorophenyl)­borane-Catalyzed
Hydride Transfer Reactions in Polysiloxane Chemistry—Piers-Rubinsztajn
Reaction and Related Processes. Molecules.

[141] Zhang J., Chen Y., Brook M. A. (2014). Reductive
Degradation
of Lignin and Model Compounds by Hydrosilanes. ACS Sustainable Chem. Eng..

[142] Feghali E., Cantat T. (2015). Room Temperature Organocatalyzed
Reductive Depolymerization of Waste Polyethers, Polyesters, and Polycarbonates. ChemSusChem.

[143] Li X. Y., Shang R., Fu M. C., Fu Y. (2015). Conversion
of Biomass-Derived Fatty Acids and Derivatives into Hydrocarbons Using
a Metal-Free Hydrodeoxygenation Process. Green
Chem..

[144] Zheng S., Liao M., Chen Y., Brook M. A. (2020). Dissolving
Used Rubber Tires. Green Chem..

[145] Garg N. K., Tan M., Johnson M. T., Wendt O. F. (2023). Selective
Valorization of Bio-derived Levulinic Acid and Its Derivatives into
Hydrocarbons and Biochemicals by Using Hydrosilylation. Chem.—Eur. J..

[146] Su S., Cao F. S., Wang S., Shen Q., Luo G., Lu Q., Song G. (2023). Organoborane-Catalysed Reductive Depolymerisation of
Catechyl Lignin under Ambient Conditions. Green
Chem..

[147] Su S., Wang L. (2026). Room-Temperature
Degradation of Lignin from Wasted
Seed Coats for the Production of Pyrocatechol/Gallol Derivatives. ChemSusChem.

[148] Porwal D., Oestreich M. (2016). B­(C_6_F_5_)_3_-Catalyzed Reduction of Aromatic and Aliphatic
Nitro Groups
with Hydrosilanes. Eur. J. Org. Chem..

[149] Süsse L., Hermeke J., Oestreich M. (2016). The Asymmetric
Piers Hydrosilylation. J. Am. Chem. Soc..

[150] Kim Y., Chang S. (2016). Borane-Catalyzed Reductive
α-Silylation of Conjugated
Esters and Amides Leaving Carbonyl Groups Intact. Angew. Chem., Int. Ed..

[151] Zhan X.-Y., Zhang H., Dong Y., Yang J., He S., Shi Z.-C., Tang L., Wang J.-Y. (2020). Chemoselective Hydrosilylation
of the α, β-Site Double Bond in α,β- and α,β,γ,δ-Unsaturated
Ketones Catalyzed by Macrosteric Borane Promoted by Hexafluoro-2-propanol. J. Org. Chem..

[152] Zhang Y., Chen Y., Zhang Z., Liu S., Shen X. (2020). Synthesis of Stereodefined Trisubstituted Alkenyl Silanes Enabled
by Borane Catalysis and Nickel Catalysis. Org.
Lett..

[153] Wang G., Su X., Gao L., Liu X., Li G., Li S. (2021). Borane-Catalyzed
Selective Dihydrosilylation of Terminal
Alkynes: Reaction Development and Mechanistic Insight. Chem. Sci..

[154] Fasano V., Radcliffe J. E., Ingleson M. J. (2016). B­(C_6_F_5_)_3_-Catalyzed Reductive Amination Using Hydrosilanes. ACS Catal..

[155] Fasano V., Ingleson M. J. (2017). Expanding Water/Base Tolerant Frustrated
Lewis Pair Chemistry to Alkylamines Enables Broad Scope Reductive
Aminations. Chem.—Eur. J..

[156] Liu Z. Y., Wen Z. H., Wang X. C. (2017). B­(C_6_F_5_)_3_-Catalyzed Cascade Reduction of
Pyridines. Angew. Chem., Int. Ed..

[157] Pan Y., Chen C., Xu X., Zhao H., Han J., Li H., Xu L., Fan Q., Xiao J. (2018). Metal-free Tandem Cyclization/Hydrosilylation
to Construct Tetrahydroquinoxalines. Green Chem..

[158] Cao V. D., Mun S. H., Kim S. H., Kim G. U., Kim H. G., Joung S. (2020). Synthesis of Cyclic
Amidines from
Quinolines by a Borane-Catalyzed Dearomatization Strategy. Org. Lett..

[159] Liu T., Wang H. (2025). Synthesis of Tetrahydroquinoline
via Hydrosilylation-Transfer
Hydrogenation of Quinoline with Silane and Water Catalyzed by a Switchable
Lewis and Brønsted Acid System. Tetrahedron.

[160] Zou F., Feng X., Du H. (2025). Chiral Borane-Catalyzed
Asymmetric
Hydrosilylation of 2,3,3-Trisubstituted 3*H*-Indoles. J. Org. Chem..

[161] Petrushko W. D., Nikonov G. I. (2020). Mono­(hydrosilylation) of N-Heterocycles
Catalyzed by B­(C_6_F_5_)_3_ and Silylium
Ion. Organometallics.

[162] Xie H., Lu J., Gui Y., Gao L., Song Z. (2017). (HMe_2_SiCH_2_)_2_: A Useful
Reagent for B­(C_6_F_5_)_3_-Catalyzed Reduction-Lactonization
of Keto
Acids: Concise Syntheses of (−)-*cis*-Whisky
and (−)-*cis*-Cognac Lactones. Synlett.

[163] Guo J., Liu S., Jing J., Fan Y., Fu Y., Liu S., Wang W., Gao L., Song Z. (2023). Controllable Si–C
Bond Formation from Trihydrosilanes En Route to Synthesis of 1,4-Azasilinanes
with Diverse Silyl Functionalities. Org. Lett..

[164] He W., Li B., Li Y., Liu X., Cui D. (2025). Reduction
Polymerization of CO_2_ with Phenylene Silanes Catalyzed
by Single-Component B­(C_6_F_5_)_3_. Angew. Chem., Int. Ed..

[165] Chen J., Falivene L., Caporaso L., Cavallo L., Chen E. Y. X. (2016). Selective Reduction of CO_2_ to CH_4_ by Tandem Hydrosilylation with Mixed Al/B Catalysts. J. Am. Chem. Soc..

[166] Wang T., Xu M., Jupp A. R., Qu Z. W., Grimme S., Stephan D. W. (2021). Selective Catalytic Frustrated Lewis
Pair Hydrogenation of CO_2_ in the Presence of Silylhalides. Angew. Chem..

[167] Jeong H., Han N., Hwang D. W., Ko H. M. (2020). B­(C_6_F_5_)_3_-Catalyzed Highly Chemoselective
Reduction of Isatins: Synthesis of Indolin-3-ones and Indolines. Org. Lett..

[168] Son S. D., Choi H. Y., Ko H. M. (2025). B­(C_6_F_5_)_3_-Catalyzed Reductive Deoxygenation of Isatins
for Indole Synthesis. J. Org. Chem..

[169] Kim Y., Dateer R. B., Chang S. (2017). Borane-Catalyzed
Selective Hydrosilylation
of Internal Ynamides Leading to β-Silyl (*Z*)-Enamides. Org. Lett..

[170] Hazra C. K., Gandhamsetty N., Park S., Chang S. (2016). Borane Catalysed
Ring Opening and Closing Cascades of Furans Leading to Silicon Functionalized
Synthetic Intermediates. Nat. Commun..

[171] Zhang J., Park S., Chang S. (2018). Piers’
Borane-Mediated
Hydrosilylation of Epoxides and Cyclic Ethers. Chem. Commun..

[172] Fegyverneki D., Kolozsvári N., Molnár D., Egyed O., Holczbauer T., Soós T. (2019). Size-Exclusion
Borane-Catalyzed Domino 1,3-Allylic/Reductive Ireland-Claisen Rearrangements:
Impact of the Electronic and Structural Parameters on the 1,3-Allylic
Shift Aptitude. Chem.—Eur. J..

[173] Ma Y., Wang B., Zhang L., Hou Z. (2016). Boron-Catalyzed Aromatic
C–H Bond Silylation with Hydrosilanes. J. Am. Chem. Soc..

[174] Long P. W., He T., Oestreich M. (2020). B­(C_6_F_5_)_3_-Catalyzed Hydrosilylation of Vinylcyclopropanes. Org. Lett..

[175] Lin R., Ou M., Ni Q., Liu X., Song Z., Huang X., Yao W., Chen Y., Long J., Song W., Ma Y. (2025). Boron Catalysis-Enabled
Regioselective
Ring-Retentive Hydrosilylation of Methylenecyclopropanes. Org. Lett..

[176] Long P. W., Oestreich M. (2021). B­(C_6_F_5_)_3_-Catalyzed Diastereoselective Formal (4 + 1)-Cycloaddition
of Vinylcyclopropanes and Et_2_SiH_2_. Org. Lett..

[177] Locmele R., Szilvagyi G., Répási J., Smits G. (2025). Selective Reductions of Esters to Aldehydes: Extended Scope and Downstream
Reactivity. J. Org. Chem..

[178] Bender T. A., Dabrowski J. A., Gagné M. R. (2016). Delineating
the Multiple Roles of B­(C_6_F_5_)_3_ in
the Chemoselective Deoxygenation of Unsaturated Polyols. ACS Catal..

[179] Chatterjee I., Porwal D., Oestreich M. (2017). B­(C_6_F_5_)_3_-Catalyzed Chemoselective Defunctionalization
of Ether-Containing Primary Alkyl Tosylates with Hydrosilanes. Angew. Chem., Int. Ed..

[180] Jeon C., Kim D. W., Chang S., Kim J. G., Seo M. (2019). Synthesis of Polypropylene via Catalytic Deoxygenation of Poly­(methyl
acrylate). ACS Macro Lett..

[181] Ni J., Oguro T., Sawazaki T., Sohma Y., Kanai M. (2018). Hydroxy Group
Directed Catalytic Hydrosilylation of Amides. Org. Lett..

[182] Mukherjee D., Shirase S., Mashima K., Okuda J. (2016). Chemoselective
Reduction of Tertiary Amides to Amines Catalyzed by Triphenylborane. Angew. Chem., Int. Ed..

[183] Mukherjee D., Sauer D. F., Zanardi A., Okuda J. (2016). Selective
Metal-Free Hydrosilylation of CO_2_ Catalyzed by Triphenylborane
in Highly Polar, Aprotic Solvents. Chem.—Eur.
J..

[184] Maity R., Das B., Das I. (2019). Transition-Metal-Free
Reduction of α-Keto Thioesters with Hydrosilanes at Room Temperature:
Divergent Synthesis through Reagent-Controlled Chemoselectivities. Adv. Synth. Catal..

[185] Peng Y., Oestreich M. (2022). B­(C_6_F_5_)_3_-Catalyzed Reductive Denitrogenation of Benzonitrile Derivatives. Org. Lett..

[186] Peng Y., Oestreich M. (2023). B­(C_6_F_5_)_3_-Catalyzed Regioselective Ring Opening of Cyclic Amines with
Hydrosilanes. Chem.—Eur. J..

[187] Nad P., Goswami S., Kisan H. K., Mukherjee A. (2025). Unlocking
the Potential of Tris­(pentafluorophenyl)­borane in Reductive Desulfurization
of Thioamides with Silane. Chem.—Eur.
J..

[188] Ding G., Wu X., Lu B., Lu W., Zhang Z., Xie X. (2018). Tris­(pentafluorophenyl)­borane-Catalyzed
Reduction of Cyclic Imides with Hydrosilanes: Synthesis of Pyrrolidines. Tetrahedron.

[189] Luo Z., Yang J., Yao Z., Yang J., Xu L., Shi Q. (2023). B­(C_6_F_5_)_3_-Catalyzed
One-Pot Tandem
Diastereoselective Synthesis of *cis*-2,3-Disubstituted
1,2,3,4-Tetrahydroquinoxalines and *cis*-2,4-Disubstituted
2,3,4,5-Tetrahydro-1*H*-1,5-benzodiazepines. Adv. Synth. Catal..

[190] Luo Z., Yang J., Yang J., Yao Z., He Z., Liu K., Xu L., Shi Q. (2024). Borane-Catalyzed Selective Transformation
of Levulinic Acid and Hydrazines into Hexahydropyridazines and Tetrahydropyridazin-3­(2*H*)-ones Using Hydrosilane. Adv. Synth.
Catal..

[191] Wang H., Yang J., Luo Z., Yao Z., Yang J., Li H., Xu L. (2024). Catalytic Deoxygenative
Reduction of Hydrazides to Hydrazines via B­(C_6_F_5_)_3_-Catalyzed Hydrosilylation. Adv.
Synth. Catal..

[192] Huang W., Luo Z., Yang J., He Z., Yao Z., Meng S., Xu L., Shi Q. (2025). Facile Access to 2-Substituted-1,2,3,4-Tetrahydroquinoxalines
via Borane-Catalyzed One-Pot Tandem Cyclization/Hydrosilylation. Adv. Synth. Catal..

[193] Chulsky K., Dobrovetsky R. (2017). B­(C_6_F_5_)_3_-Catalyzed
Selective Chlorination of Hydrosilanes. Angew.
Chem..

[194] Chulsky K., Dobrovetsky R. (2018). Metal-Free Catalytic Reductive Cleavage
of Enol Ethers. Org. Lett..

[195] Yang W., Gao L., Lu J., Song Z. (2018). Chemoselective
Deoxygenation of Ether-Substituted Alcohols and Carbonyl Compounds
by B­(C_6_F_5_)_3_-Catalyzed Reduction with
(HMe_2_SiCH_2_)_2_. Chem. Commun..

[196] Richter S. C., Oestreich M. (2019). Bioinspired Metal-Free Formal Decarbonylation
of α-Branched Aliphatic Aldehydes at Ambient Temperature. Chem.—Eur. J..

[197] Beh D. W., Piers W. E., Gelfand B. S., Lin J. B. (2020). Tandem
Deoxygenative Hydrosilation of Carbon Dioxide with a Cationic Scandium
Hydridoborate and B­(C_6_F_5_)_3_. Dalton Trans..

[198] Han F., Lu G. S., Wu D. P., Huang P. Q. (2023). Iridium and B­(C_6_F_5_)_3_ Co-catalyzed Chemoselective Deoxygenative
Reduction of Tertiary Amides: Application to the Efficient Synthesis
and Late-Stage Modification of Pharmaceuticals. Sci. China: Chem..

[199] Fang H., Oestreich M. (2020). Reductive Deamination with Hydrosilanes
Catalyzed by B­(C_6_F_5_)_3_. Angew. Chem., Int. Ed..

[200] Ma P., Guo T., Lu H. (2024). Hydro- and Deutero-Deamination of
Primary Amines Using *O*-Diphenylphosphinylhydroxylamine. Nat. Commun..

[201] Tsuji T., Fukumoto I., Hario T., Hayashi M., Osawa A., Ohshima T., Yazaki R. (2025). Catalytic
Diazene Synthesis
from Sterically Hindered Amines for Deaminative Functionalization. Nat. Commun..

[202] Bender T. A., Dabrowski J. A., Zhong H., Gagné M. R. (2016). Diastereoselective
B­(C_6_F_5_)_3_-Catalyzed Reductive Carbocyclization
of Unsaturated Carbohydrates. Org. Lett..

[203] Zhang Z., Sun Q., Xia C., Sun W. (2016). CO_2_ as a C1 Source: B­(C_6_F_5_)_3_-Catalyzed
Cyclization of *o*-Phenylenediamines to Construct Benzimidazoles
in the Presence of Hydrosilane. Org. Lett..

[204] Tiddens M. R., Klein Gebbink R. J. M., Otte M. (2016). The B­(C_6_F_5_)_3_-Catalyzed
Tandem Meinwald Rearrangement-Reductive
Amination. Org. Lett..

[205] Mehta M., Garcia de la Arada I., Perez M., Porwal D., Oestreich M., Stephan D. W. (2016). Metal-Free Phosphine Oxide Reductions
Catalyzed by B­(C_6_F_5_)_3_ and Electrophilic
Fluorophosphonium Cations. Organometallics.

[206] Ding F., Jiang Y., Gan S., Bao R. L.-Y., Lin K., Shi L. (2017). B­(C_6_F_5_)_3_-Catalyzed Deoxygenation of Sulfoxides and Amine
N-Oxides
with Hydrosilanes. Eur. J. Org. Chem..

[207] Porwal D., Oestreich M. (2017). B­(C_6_F_5_)_3_-Catalyzed Reduction of Sulfoxides and
Sulfones to Sulfides
with Hydrosilanes. Synthesis.

[208] Drosos N., Cheng G. J., Ozkal E., Cacherat B., Thiel W., Morandi B. (2017). Catalytic Reductive
Pinacol-Type
Rearrangement of Unactivated 1,2-Diols through a Concerted, Stereoinvertive
Mechanism. Angew. Chem., Int. Ed..

[209] Fang H., Wang G., Oestreich M. (2021). Mild Reductive
Rearrangement of Oximes and Oxime Ethers to Secondary Amines with
Hydrosilanes Catalyzed by B­(C_6_F_5_)_3_. Org. Chem. Front..

[210] Xu K., Zhang Z., Li B., Li J., Loh T. P., Jia Z. (2025). B­(C_6_F_5_)_3_-Catalyzed Reductive C-C
Bond Cleavage of Bisindolylmethanes. Adv. Synth.
Catal..

[211] Qiao C., Liu X. F., Fu H. C., Yang H. P., Zhang Z. B., He L. N. (2018). Directly Bridging
Indoles to 3,3′-Bisindolylmethanes
by Using Carboxylic Acids and Hydrosilanes under Mild Conditions. Chem.—Asian J..

[212] Li M., Wang T., An Z., Yan R. (2020). B­(C_6_F_5_)_3_-Catalyzed Cyclization of Alkynes: Direct Synthesis
of 3-Silyl Heterocyclic Compounds. Chem. Commun..

[213] Tang L., Zang Y., Guo W., Han Z., Huang H., Sun J. (2022). Reductive Opening of Oxetanes Catalyzed
by Frustrated Lewis Pairs: Unexpected Aryl Migration via Neighboring
Group Participation. Org. Lett..

[214] Peng Y., Wang G., Klare H. F. T., Oestreich M. (2024). Ring Contraction
of Saturated Cyclic Amines and Rearrangement of Acyclic Amines Through
Their Corresponding Hydroxylamines. Angew. Chem.,
Int. Ed..

[215] Zotov V. V., Zupancic C., Bailey Z. J., Du G. (2024). Ring Opening
Reduction of Cyclic Anhydrides Catalyzed by Tris­(pentafluorophenyl)­borane
Using Hydrosilanes as a Hydride Source. J. Org.
Chem..

[216] Amber C., Petitjean T. M., Sirvinskaite G., Steele R. T., Sprague B., Domack J., Small D. W., Sarpong R. (2025). Two-Step Constitutional
Isomerization of Saturated
Cyclic Amines Using Borane Catalysis. JACS Au.

[217] Fan Y., Jing J., Li X., Jiang X., Wang W., Gao L., Song Z. (2026). B­(C_6_F_5_)_3_-Catalyzed
(5 + 1)-Annulation of Enynamides with Trihydrosilanes en Route to
the Synthesis of 1,4-Azasilines. Org. Lett..

[218] Hantzsch A. (1881). Condensationsprodukte aus Aldehydammoniak
und ketonartigen
Verbindungen. Ber. Dtsch. Chem. Ges..

[219] Zheng C., You S.-L. (2012). Transfer Hydrogenation
with Hantzsch
Esters and Related Organic Hydride Donors. Chem.
Soc. Rev..

[220] You S.-L. (2007). Recent
Developments in Asymmetric Transfer Hydrogenation
with Hantzsch Esters: A Biomimetic Approach. Chem.—Asian J..

[221] Ouellet S. G., Walji A. M., MacMillan D. W. C. (2007). Enantioselective
Organocatalytic Transfer Hydrogenation Reactions Using Hantzsch Esters. Acc. Chem. Res..

[222] Rueping M., Dufour J., Schoepke F. R. (2011). Advances in Catalytic
Metal-Free Reductions: From Bio-Inspired Concepts to Applications
in the Organocatalytic Synthesis of Pharmaceuticals and Natural Products. Green Chem..

[223] Webb J. D., Laberge V. S., Geier S. J., Stephan D. W., Crudden C. M. (2010). Borohydrides
from Organic Hydrides: Reactions of Hantzsch’s
Esters with B­(C_6_F_5_)_3_. Chem.—Eur. J..

[224] Hamasaka G., Tsuji H., Uozumi Y. (2015). Organoborane-Catalyzed
Hydrogenation of Unactivated Aldehydes with a Hantzsch Ester as a
Synthetic NAD­(P)H Analogue. Synlett.

[225] Mahesh S., Anand R. V. (2017). B­(C_6_F_5_)_3_ Catalysed Reduction of *para*-Quinone Methides
and Fuchsones to Access Unsymmetrical Diaryl- and Triarylmethanes:
Elaboration to Beclobrate. Org. Biomol. Chem..

[226] Wang Q., Chen J., Feng X., Du H. (2018). B­(C_6_F_5_)_3_-Catalyzed Transfer Hydrogenations
of Imines
with Hantzsch Esters. Org. Biomol. Chem..

[227] Hamasaka G., Tsuji H., Ehara M., Uozumi Y. (2019). Mechanistic
Insight into the Catalytic Hydrogenation of Nonactivated Aldehydes
with a Hantzsch Ester in the Presence of a Series of Organoboranes:
NMR and DFT Studies. RSC Adv..

[228] Faverio C., Boselli M. F., Medici F., Benaglia M. (2020). Ammonia Borane
as a Reducing Agent in Organic Synthesis. Org.
Biomol. Chem..

[229] Lau S., Gasperini D., Webster R. L. (2021). Amine-Boranes as Transfer Hydrogenation
and Hydrogenation Reagents: A Mechanistic Perspective. Angew. Chem., Int. Ed..

[230] Hamann H. J., Ramachandran P. V. (2026). Recent Developments in the Synthesis
and Synthetic Applications of Borane-Amines. Chem. Commun..

[231] Patrick E. A., Piers W. E. (2020). Twenty-Five Years of Bis-Pentafluorophenyl
Borane: A Versatile Reagent for Catalyst and Materials Synthesis. Chem. Commun..

[232] Li S., Li G., Meng W., Du H. (2016). A Frustrated Lewis
Pair Catalyzed Asymmetric Transfer Hydrogenation of Imines Using Ammonia
Borane. J. Am. Chem. Soc..

[233] Zhou Q., Zhang L., Meng W., Feng X., Yang J., Du H. (2016). Borane-Catalyzed Transfer
Hydrogenations
of Pyridines with Ammonia Borane. Org. Lett..

[234] Ding F., Zhang Y., Zhao R., Jiang Y., Bao R. L.-Y., Lin K., Shi B. (2017). B­(C_6_F_5_)_3_-Promoted Hydrogenations of N-Heterocycles
with
Ammonia Borane. Chem. Commun..

[235] Li S., Meng W., Du H. (2017). Asymmetric
Transfer Hydrogenations
of 2,3-Disubstituted Quinoxalines with Ammonia Borane. Org. Lett..

[236] Pan Z., Shen L., Song D., Xie Z., Ling F., Zhong W. (2018). B­(C_6_F_5_)_3_-Catalyzed Asymmetric Reductive
Amination of Ketones with Ammonia Borane. J.
Org. Chem..

[237] Zhao W., Feng X., Yang J., Du H. (2019). Asymmetric
Transfer Hydrogenations of β-N-Substituted Enamino Esters with
Ammonia Borane. Tetrahedron Lett..

[238] Zhao W., Zhang Z., Feng X., Yang J., Du H. (2020). Asymmetric Transfer Hydrogenation
of N-Unprotected Indoles with Ammonia
Borane. Org. Lett..

[239] Pan Y., Luo Z., Han J., Xu X., Chen C., Zhao H., Xu L., Fan Q., Xiao J. (2019). B­(C_6_F_5_)_3_-Catalyzed Deoxygenative
Reduction of Amides
to Amines with Ammonia Borane. Adv. Synth. Catal..

[240] Guo X., Unglaube F., Kragl U., Mejia E. (2022). B­(C_6_F_5_)_3_-Catalyzed Transfer Hydrogenation
of Esters and
Organic Carbonates toward Alcohols with Ammonia Borane. Chem. Commun..

[241] Keess S., Oestreich M. (2017). Cyclohexa-1,4-dienes in Transition-Metal-Free
Ionic Transfer Processes. Chem. Sci..

[242] Walker J. C. L., Oestreich M. (2019). Ionic Transfer
Reactions with Cyclohexadiene-Based
Surrogates. Synlett.

[243] Sakata K., Fujimoto H. (2015). Quantum Chemical Study
of the Reaction
of 3-(Trimethylsilyl)­cyclohexa-1,4-dienes with Tris­(pentafluorophenyl)­borane. Organometallics.

[244] Simonneau A., Oestreich M. (2013). 3-Silylated Cyclohexa-1,4-dienes
as Precursors for Gaseous Hydrosilanes: The B­(C_6_F_5_)_3_-Catalyzed Transfer Hydrosilylation of Alkenes. Angew. Chem., Int. Ed..

[245] Keeß S., Simonneau A., Oestreich M. (2015). Direct and
Transfer Hydrosilylation Reactions Catalyzed by Fully or Partially
Fluorinated Triarylboranes: A Systematic Study. Organometallics.

[246] Chatterjee I., Oestreich M. (2015). B­(C_6_F_5_)_3_-Catalyzed
Transfer Hydrogenation of Imines and Related Heteroarenes
Using Cyclohexa-1,4-dienes as a Dihydrogen Source. Angew. Chem., Int. Ed..

[247] Chatterjee I., Oestreich M. (2016). Brønsted Acid-Catalyzed Transfer
Hydrogenation of Imines and Alkenes Using Cyclohexa-1,4-dienes as
Dihydrogen Surrogates. Org. Lett..

[248] Chatterjee I., Qu Z.-W., Grimme S., Oestreich M. (2015). B­(C_6_F_5_)_3_-Catalyzed
Transfer of Dihydrogen from
One Unsaturated Hydrocarbon to Another. Angew.
Chem., Int. Ed..

[249] Yuan W., Orecchia P., Oestreich M. (2017). Cyclohexa-1,3-diene-Based
Dihydrogen and Hydrosilane Surrogates in B­(C_6_F_5_)_3_-Catalysed Transfer Processes. Chem. Commun..

[250] Keeß S., Oestreich M. (2017). 3-*tert*-Butyl-Substituted
Cyclohexa-1,4-dienes as Isobutane Equivalents in the B­(C_6_F_5_)_3_-Catalyzed Transfer Hydro-*tert*-Butylation of Alkenes. Chem.—Eur. J..

[251] Walker J. C. L., Oestreich M. (2019). Lewis Acid
Catalyzed Transfer Hydromethallylation
for the Construction of Quaternary Carbon Centers. Angew. Chem., Int. Ed..

[252] Keeß S., Oestreich M. (2017). Access to Fully Alkylated Germanes
by B­(C_6_F_5_)_3_-Catalyzed Transfer Hydrogermylation
of Alkenes. Org. Lett..

[253] Orecchia P., Yuan W., Oestreich M. (2019). Transfer Hydrocyanation
of α- and α,β-Substituted Styrenes Catalyzed by
Boron Lewis Acids. Angew. Chem., Int. Ed..

[254] Xie K., Oestreich M. (2022). Decarbonylative
Transfer Hydrochlorination of Alkenes
and Alkynes Based on a B­(C_6_F_5_)_3_-Initiated
Grob Fragmentation. Angew. Chem., Int. Ed..

[255] Banerjee S., Vanka K. (2018). B­(C_6_F_5_)_3_: Catalyst or Initiator? Insights from Computational
Studies
into Surrogate Silicon Chemistry. ACS Catal..

[256] Simonneau A., Oestreich M. (2015). Formal SiH_4_ Chemistry
Using Stable and Easy-to-Handle Surrogates. Nat. Chem..

[257] Simonneau, A. ; Oestreich, M. Use of cyclohexa-2,5-dien-1-yl-silanes as precursors for gaseous hydrosilanes. WO 2015036309 A1, 2015.

[258] Lefranc I., Qu Z.-W., Grimme S., Oestreich M. (2016). Hydrogenation
and Transfer Hydrogenation Promoted by Tethered Ru-S Complexes: From
Cooperative Dihydrogen Activation to Hydride Abstraction/Proton Release
from Dihydrogen Surrogates. Chem.—Eur.
J..

[259] Walker J. C. L., Oestreich M. (2018). Regioselective Transfer Hydrodeuteration
of Alkenes with a Hydrogen Deuteride Surrogate Using B­(C_6_F_5_)_3_ Catalysis. Org.
Lett..

[260] Li L., Hilt G. (2020). Regiodivergent
DH or HD Addition to Alkenes: Deuterohydrogenation
versus Hydrodeuterogenation. Org. Lett..

[261] Liu Z., Shi Z.-J., Liu L., Zhang M., Zhang M.-C., Guo H.-Y., Wang X.-C. (2023). Asymmetric
C3-Allylation of Pyridines. J. Am. Chem. Soc..

[262] Liu Z., He J.-H., Zhang M., Shi Z.-J., Tang H., Zhou X.-Y., Tian J.-J., Wang X.-C. (2022). Borane-Catalyzed
C3-Alkylation of Pyridines with Imines, Aldehydes, or Ketones as Electrophiles. J. Am. Chem. Soc..

[263] Tian J.-J., Li R.-R., Tian G.-X., Wang X.-C. (2023). Enantioselective
C3-Allylation of Pyridines via Tandem Borane and Palladium Catalysis. Angew. Chem., Int. Ed..

[264] Zhou X.-Y., Zhang M., Liu Z., He J.-H., Wang X.-C. (2022). C3-Selective Trifluoromethylthiolation and Difluoromethylthiolation
of Pyridines and Pyridine Drugs via Dihydropyridine Intermediates. J. Am. Chem. Soc..

[265] Lawson J. R., Wilkins L. C., Melen R. L. (2017). Tris­(2,4,6-trifluorophenyl)­borane:
An Efficient Hydroboration Catalyst. Chem.—Eur.
J..

[266] Yin Q., Kemper S., Klare H. F. T., Oestreich M. (2016). Boron Lewis
Acid-Catalyzed Hydroboration of Alkenes with Pinacolborane: BArF_3_ Does What B­(C_6_F_5_)_3_ Cannot
Do!. Chem.—Eur. J..

[267] Bismuto A., Cowley M. J., Thomas S. P. (2021). Zwitterion-Initiated
Hydroboration of Alkynes and Styrene. Adv. Synth.
Catal..

[268] Sieland B., Hoppe A., Stepen A. J., Paradies J. (2022). Frustrated
Lewis Pair-Catalyzed Hydroboration of Nitriles: FLP versus Borenium
Catalysis. Adv. Synth. Catal..

[269] McGough J. S., Butler S. M., Cade I. A., Ingleson M. J. (2016). Highly
Selective Catalytic trans-Hydroboration of Alkynes Mediated by Borenium
Cations and B­(C_6_F_5_)_3_. Chem. Sci..

[270] Lowe J. M., Seo Y., Gagné M. R. (2018). Boron-Catalyzed
Site-Selective Reduction of Carbohydrate Derivatives with Catecholborane. ACS Catal..

[271] Kim E., Jeon H. J., Park S., Chang S. (2020). Double Hydroboration
of Quinolines via Borane Catalysis: Diastereoselective One-Pot Synthesis
of 3-Hydroxytetrahydroquinolines. Adv. Synth.
Catal..

[272] Zhang S., Xu H., He J., Zhang Y. (2021). Application
of Mutualism in Organic Synthetic Chemistry: Mutually Promoted C-H
Functionalization of Indole and Reduction of Quinoline. Adv. Synth. Catal..

[274] Liu T., He J., Zhang Y. (2025). An Efficient Method for Constructing
C3-Stannyl-Tetrahydroquinoline: Cascade Hydroboration and Hydrostannation
of Quinoline. Org. Biomol. Chem..

[275] Wu R., Gao K. (2021). B­(C_6_F_5_)_3_-Catalyzed
Tandem Protonation/Deuteration and Reduction of in Situ-Formed Enamines. Org. Biomol. Chem..

[276] Zou W., Gao L., Cao J., Li Z., Li G., Wang G., Li S. (2022). Mechanistic Insight into Hydroboration
of Imines from Combined Computational and Experimental Studies. Chem.—Eur. J..

[277] Zhang S., Han Y., He J., Zhang Y. (2018). B­(C_6_F_5_)_3_-Catalyzed C3-Selective C-H Borylation
of Indoles: Synthesis, Intermediates, and Reaction Mechanism. J. Org. Chem..

[278] Nad P., Mukherjee A. (2023). A Lewis Acid-Base Pair Catalyzed Dearomative Transformation
of Unprotected Indoles via B-H Bond Activation. Chem.—Asian J..

[279] Zhang J., Chen Z., Chen M., Zhou Q., Zhou R., Wang W., Shao Y., Zhang F. (2024). Lanthanide/B­(C_6_F_5_)_3_-Promoted Hydroboration Reduction
of Indoles and Quinolines with Pinacolborane. J. Org. Chem..

[280] Wang W., Hu Y., Yang J., Zhang S., Zhang Y., Jin Q., Xu M., Wang Q., Shao Y., Zhang F. (2024). LuCl_3_/B­(C_6_F_5_)_3_ Cocatalyzed Reductive Deoxygenation of Ketones,
Aldehydes, Alcohols, and Ethers to Alkanes with Pinacolborane. Org. Lett..

[281] Chen W., Zheng W., Xu M., Jin Q., Yang J., Hu Y., Jiang R., Shao Y., Zhang F. (2025). B­(C_6_F_5_)_3_-Catalyzed Direct Deoxygenative
Alkenylation of Ketones with Pinacolborane. J. Org. Chem..

[282] Huang B.-H., Sontakke G. S., Cheng Y.-H., Shen J.-S., Weng Y.-Z., Xiao H.-X., Yu C.-H., Yap G. P. A., Wang T.-H., Cheng M.-J., Aweke B. S., Ong T.-G. (2025). Frustrated
Lewis Pair (FLP) Reactivity from Carbone-BPh_3_ Lewis Adduct. Chem.—Eur. J..

[283] Fillion E., Kavoosi A., Nguyen K., Ieritano C. (2016). B­(C_6_F_5_)_3_-Catalyzed Transfer 1,4-Hydrostannylation
of α,β-Unsaturated Carbonyl Compounds Using iPr-Tricarbastannatrane. Chem. Commun..

[284] Yan T., Li Y., Huang G., Ni S., Dang L. (2022). Reaction Mechanism
Study on Reactions of Phenylacetylenes with HSnEt_3_ Promoted
by B­(C_6_F_5_)_3_ with and without DABCO. Org. Chem. Front..

[285] Irrgang T., Kempe R. (2020). Transition-Metal-Catalyzed Reductive
Amination Employing Hydrogen. Chem. Rev..

